# Maternal and Pediatric Precision in Therapeutic Knowledge Portal (MPRINT‐KP): Landscape Analysis of Pharmacology Research in Maternal and Pediatric Patient Populations

**DOI:** 10.1002/phar.70096

**Published:** 2026-02-23

**Authors:** Xiaofu Liu, Aditi Shendre, Lei Wang, Aislinn O'Kane, Hanson Ma, Chien‐Wei Chiang, Syed S. Zaidi, Omar A. Aboshady, Gerald So, Emma M. Tillman, Lindsey M. Kirkpatrick, Maged Costantine, Shaun Grannis, Colin M. Rogerson, Christopher Bartlett, Saurabh Rahurkar, Lijun Cheng, Jiayi Ouyang, Ping Wei, Zhimo Zhu, Shangjia Li, Yirui Huang, Lingling Wang, Weidan Cao, Haoran Jiang, Jianing Liu, Samuel‐Richard Oteng, Andrew Goodwin, Jiezel Ann Faith Deypalubos, Shijun Zhang, Robert Bies, Sara K. Quinney, Lang Li

**Affiliations:** ^1^ Department of Biomedical Informatics, College of Medicine The Ohio State University Columbus Ohio USA; ^2^ Division of Clinical Pharmacology, Department of Medicine, School of Medicine Indiana University Indianapolis Indiana USA; ^3^ Department of Pharmacy Practice, College of Pharmacy Purdue University West Lafayette Indiana USA; ^4^ Department of Pediatrics, School of Medicine Indiana University Indianapolis Indiana USA; ^5^ Division of Maternal and Fetal Medicine, Department of Obstetrics and Gynecology, College of Medicine The Ohio State University Columbus Ohio USA; ^6^ Department of Family Medicine, School of Medicine Indiana University Indianapolis Indiana USA; ^7^ Regenstrief Institute Indianapolis Indiana USA; ^8^ Department of Pediatric, College of Medicine The Ohio State University Columbus Ohio USA; ^9^ College of Pharmacy The Ohio State University Columbus Ohio USA; ^10^ Department of Pharmaceutical Science, School of Pharmacy and Pharmaceutical Science University of Buffalo Buffalo New York USA; ^11^ Department of Obstetrics and Gynecology, School of Medicine Indiana University Indianapolis Indiana USA

**Keywords:** artificial intelligence, clinical trials as topic, knowledge bases, maternal‐child nursing, pharmacoepidemiology, pharmacokinetics, therapeutics

## Abstract

Pregnant women and children have been underrepresented in clinical studies due to ethical concerns and perceived vulnerabilities. This resulted in a significant gap in knowledge regarding the safety and efficacy of medications for these populations. Maternal and Pediatric PRecision In Therapeutics Knowledge Portal (MPRINT‐KP) is designed to provide a comprehensive view of pharmacokinetic, pharmaco‐epidemiology, and clinical trial research evidence in maternal and pediatric patient populations. MPRINT‐KP Silver was generated upon BioBERT models, due to their supreme performance in classifying pharmacokinetic, pharmaco‐epidemiology, and clinical trial papers from PubMed, with an F1‐score > 0.91. As of April 1, 2025, MPRINT‐KP Silver contains 758,560 clinical pharmacology research papers in maternal and pediatric populations. The landscape analysis revealed a large number of drugs with pharmacology knowledge gaps, which is calculated as the relative frequency of drugs with no or weak (i.e., less than five publications) evidence in each of pharmacokinetic, pharmaco‐epidemiology, or clinical trial study type. The highest pharmacotherapy knowledge gaps are in postpartum women, pregnant women, and pediatric patients between 0–12 and 12–18 years of age (51.37%, 32.47%, 25.82%, 32.11%, respectively). Pharmacovigilance analyses were conducted on highly prescribed drugs with limited pharmacology evidence in pediatric patients 0–2 year old and pregnant women using United States Food and Drug Administration Adverse Event Reporting System (FAERS) and MarketScan claims data. A number of new drug‐associated adverse drug events (ADEs) were discovered. In pediatric patients, there were crisaborole‐associated hemorrhage and moxifloxacin‐associated cardiac toxicities. In pregnant women, the analysis revealed terconazole‐associated abnormalities of heartbeat; benzonatate‐associated depressive and anxiety disorders; buspirone‐associated abnormalities of heartbeat; cyclobenzaprine‐associated tubulo‐interstitial nephritis; and pantoprazole‐associated hearing loss and voice and resonance disorders. For the first time, a large‐scale landscape analysis of pharmacotherapy knowledge gap in both maternal and pediatric patient populations was conducted and identified new drug associated ADE evidence using real world data.

## 1 Introduction

**FIGURE 1:**
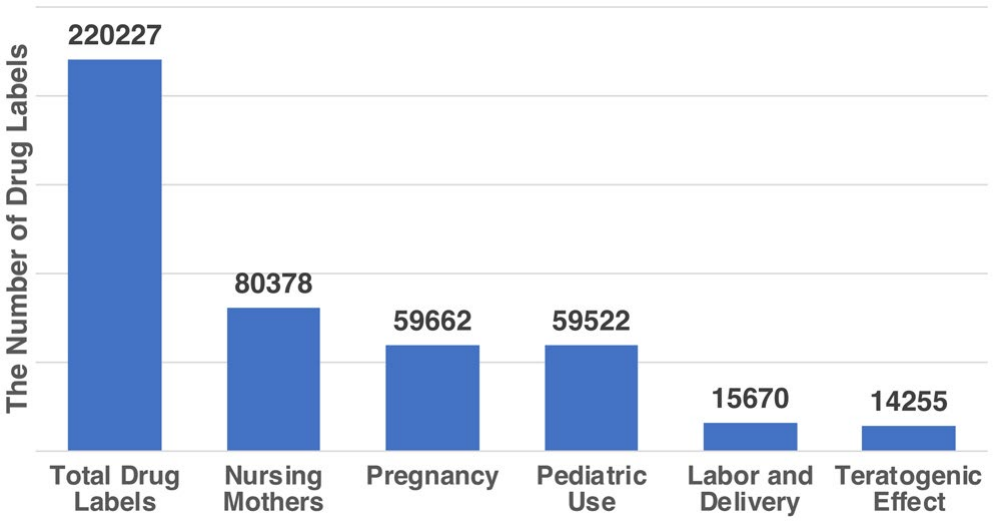
The frequencies of drug labels containing data for the corresponding sections on maternal and pediatric patient populations. Analysis based on FDA drug label data Ref. [3].

Despite the increasing use of pharmacotherapy, pregnant and breastfeeding women and pediatric patients have been underrepresented in clinical trials due to ethical concerns and perceived vulnerabilities [1]. This underrepresentation has led to significant gaps in our understanding of medication safety and efficacy in these complex populations. Drug labels often carry only generic, cautionary statements like “use only if the potential benefit justifies the potential risk to the fetus” or “safety and effectiveness in pediatric patients have not been established” [1, 2] without providing detailed population‐specific guidance. Among 220,227 drug labels from United States Food and Drug Administration (FDA) [3], only about 30% contain data in nursing mothers, pregnancy, or pediatric use; and fewer than 10% address issues such as teratogenic risks or labor and delivery (Figure 1).

Legislative and regulatory initiatives, including the Best Pharmaceuticals for Children Act (BPCA) [2] and the FDA's Pregnancy and Lactation Labeling Rule (PLLR) [1], have endeavored to mitigate these deficiencies. The BPCA promoted pediatric exclusivity programs and expanded funding for pediatric studies, while the PLLR replaced simplistic categorical labels with narrative risk assessments for pregnant and breastfeeding women. Nonetheless, critical gaps remain especially in areas like lactation and postpartum care. The clinical need is underscored by the prevalence of conditions, such as pregnancy‐associated hypertension and gestational diabetes, which affect a significant proportion of pregnant women [4, 5], and by the increasing burden of infectious, respiratory, and neurodevelopmental disorders in children. Moreover, distinct pharmacokinetic (PK) profiles due to physiological and developmental variations further complicate drug dosing. For example, neonates show increased extracellular fluid and immature metabolic enzyme systems, whereas pregnant women experience changes in body water, fat stores, and enzyme activity that influence drug distribution and clearance. In the meantime, there is minimal data in women between delivery and 6 weeks postpartum to assess the rate at which enzyme and transporter activity and other physiologic changes that impact PK will return to the “nonpregnant” state. It is also likely that timing and type of delivery and breastfeeding may lead to variability in these changes.

Despite the growing volume of pharmacological research, significant gaps persist in our knowledge of drug safety and efficacy for maternal and pediatric populations. Critical areas, including PK, pharmacoepidemiologic (PE) studies, and clinical trials (CT), remain underrepresented in the literature. Only 36% of current drug labels have information for nursing mothers and 27% of current drug labels have information for pregnant or pediatric patients, with only 6.5% of labels addressing teratogenicity and 7% including information for use during labor and delivery (Figure 1). This deficiency hinders evidence‐based decision‐making for these vulnerable groups and limits the adoption of precision therapeutic strategies in clinical practice.

Starting in 2022, a collaboration between The Ohio State University and Indiana University worked together within the Maternal and Pediatric PRecision In Therapeutics (MPRINT) Hub to design a knowledge portal, Maternal and Pediatric Precision In Therapeutics Knowledge Portal (MPRINT‐KP), for maternal and pediatric pharmacotherapy data. MPRINT‐KP is tasked to extract and integrate data from published PK studies, PE studies, and CT. Goals of the MPRINT‐KP include identifying knowledge gaps in maternal and pediatric pharmacotherapy research and highlighting drugs that have adequate data with which to support label changes or updates in dosing guidelines. In our first publication using MPRINT‐KP, we performed landscape analysis of opioids pharmacotherapy knowledge gap in maternal and pediatric patient populations [6]. In another publication, using both MPRINT‐KP and real world data, we discovered PE knowledge gap for benzonatate in pediatric patients and discovered benzonatate‐associated serious adverse events, such as seizure and arrhythmia [7]. This article focuses on the landscape and knowledge gap analysis of pharmacotherapy research among maternal and pediatric patients. In MPRINT‐KP, we create two knowledgebases. The silver version knowledgebase (i.e., MPRINT‐KP Silver) is fully generated by a *Natural Language Processing* (NLP) BioBERT [8] model to automatically classify P, PE, and CT studies in various maternal and pediatric subpopulations.

## 2 Materials and Methods

### 2.1 PubMed Queries

Papers included in the MPRINT‐KP Silver originated from a PubMed query. In order to maximize the recall of relevant maternal‐ and pediatric‐related research, the PubMed query was performed through a two‐step process. In step one, the first version of key words, including maternal and pediatric keywords, was composed based on collective expertise among 16 data curators who labeled training data for the MPRINT‐KP Silver. After relevant PK, PE, and CT papers were annotated for the training data (see below), their MeSH [9] terms were collected. In step two, these MeSH terms were used to repeat the PubMed query. We excluded reviews, in vitro studies, and animal studies in the current MPRINT‐KP Silver. More details are shown in Tables S1 and S2.

### 2.2 Annotation Guidelines for Paper Classification PK, PE, and CT

A set of 4845 abstracts was randomly selected from 3,547,720 abstracts identified by the first PubMed query. This data set served as the corpus for PK, PE, and CT abstracts. Each abstract was then annotated to identify whether it fit inclusion and exclusion criteria and was relevant to maternal‐pediatric pharmacology (Yes/No). Relevant abstracts were further annotated by type of study: PK, PE, or CT. The annotation guidelines are shown in Table 1. Annotation required data curators to review information on patient subpopulations, therapeutics, biomarkers, PK, PE, or CT studies from abstracts. PE and CT labels were considered to be mutually exclusive, but a PE or CT abstract could also include PK data. Each abstract was independently annotated by two data curators. The disagreed labels were adjudicated by the third data curator. In total, 16 data curators contributed to labeling abstracts in this corpus. This labeled corpus can be downloaded via https://github.com/langli‐lab/mprint‐kp‐data.

**TABLE 1:** Annotation guideline: PK, PE, and CT papers in PubMed.

*Inclusion criteria*	
Patient subpopulations	Maternal patients (contraception, prenatal, fertility, pregnancy, labor and delivery, lactation, postpartum). Pediatric patients (preterm, neonate, infant, children, adolescents).
Therapeutics	Include drugs, endogenous, exogenous compounds, (e.g., chemicals), and oxygen. Include vaccine and cell therapy. Include a protein/peptide, because it is part of endogenous, exogenous compounds, or similar to biologic drugs. Include smoking. Include food only if a specific ingredient of food is studied. Include studies evaluating breastfeeding (if there is a comparator group, e.g., formula). Include nutrition only if a specific nutrition element is studied. Include pollution only if a pollutant is mentioned by name.
Biomarkers	Include biomarker studies only when they are associated with therapeutic response. The biomarkers can be endogenous, exogenous compounds, (e.g., chemicals), virus, vaccine, cells or cell types, and protein or peptide. The biomarker can be mRNA, miRNA, DNA mutations, DNA methylation or other epigenetics biomarkers, if associated with response to drug. The biomarkers can be image.
Pharmacokinetics studies	Include PK/PD modeling papers. Include prospective or retrospective PK studies. Include assay (e.g., LC/MS) development papers for drug/metabolite measurement if some mention of maternal/pediatric samples.
Pharmaco‐epidemiology studies and clinical trials	Include prospective or retrospective pharmacoepidemiology studies. Include all clinical trials. Drug therapies may be either a primary or secondary objective of the paper. Include epidemiological survey paper on medication prescription or medication use frequencies in either maternal or pediatric populations.
*Exclusion criteria*	
Study types	Exclude in vitro studies. Some in vitro studies are conducted from processed maternal and pediatric tissue samples (i.e., ex vivo, placental perfusion). The outcome of these in vitro studies are NOT direct measurement from maternal and pediatric tissue samples. Exclude animal models. Exclude review papers and meta‐analyses. Exclude health system usability studies and health policy studies on medication usage
Biomarkers	Exclude virus as disease biomarker, e.g., HIV. Exclude biomarkers such as mRNA, miRNA, DNA methylation, DNA mutations, or other epigenetics biomarkers if only used as disease biomarker Exclude image if only used as disease biomarker
*Classification criteria for PK, PE, and CT paper*	
Clinical trials (CT)	Typically, a randomized clinical trial comparing a drug treatment's efficacy and/or toxicity to control and/or placebo. This trial must include randomization or use an external control population without randomization. The external control is typically derived from an existing data set, such as data from control arms of existing randomized clinical trials, a registry database, or a heath record database.
Pharmacokinetics studies (PK)	Studies quantitative aspects of drug exposure, endogenous, exogenous compounds, (e.g., chemicals), or nutrition in patients or healthy volunteers. PK studies may assess either *a drug or a biomarker*. It includes studying drug/biomarker exposure in mothers and/or their babies during pregnancy, labor, delivery, postpartum or lactation. The drug concentration or other PK parameters may be measured from different specimen samples such as blood, umbilical cord, urine, milk, etc. The PK parameters include but are not limited to clearance, half‐life, volume of distribution, absorption rate, eliminate rate, steady state concentration, maximum concentration, drug/metabolite ratio. Papers that study pharmacokinetics modeling are also included, including papers that focus on pharmacokinetics methodology development, or an assay development paper. *A paper can be classified as PK, regardless of whether it is a CT or a PE study*.
Pharmacoepidemiology studies (PE)	Studies drug efficacy or adverse events in an observational study. The observational study can be a prospective study or a retrospective study, in which study population and samples are derived from an existing cohort. This existing cohort, however, can be derived from a clinical trial, a prospective study, a health record database, or other existing databases. PE studies also include case studies. *If a paper is classified as a CT, it cannot be a PE paper. CT and PE are mutually exclusive*.

Abbreviations: CT, clinical trial; DNA, deoxyribonucleic acid; HIV, human immunodeficiency virus; LC/MS, liquid chromatography–mass spectrometry; miRNA, microRNA; mRNA, messenger RNA; PD, pharmacodynamic; PE, pharmaco‐epidemiology; PK, pharmacokinetics.

### 2.3 BioBERT Model Training and Validation

BioBERT, the Bidirectional Encoder Representations from Transformers for Biomedical Text Mining, is a biomedical domain‐specific language representation model [8]. Sharing the same architecture as the standard BERT model, BioBERT consists of a stack of 12 bidirectional Transformer encoder layers with 12 attention heads and a hidden size of 768. The multi‐head self‐attention mechanism within the model allows the model to interpret the relationship between distant words, which is particularly advantageous for processing complex pharmacological texts. In the meantime, BioBERT is pretrained on PubMed abstracts and PubMed Central (PMC) full‐text articles, which extensively exposed it to biomedical literature and then effectively captured complex domain‐specific terminology and semantic relationships, thus addressing the limitations of general‐domain models in processing biomedical text.

Using the labeled data from the corpus, three different BioBERT models were trained to identify relevant papers and classify them as PK, PE, or CT publications. Each BioBERT model was trained to predict one study type versus non‐relevant papers. Each training sample was generated by concatenating the title, abstract, MeSH terms, and keywords of each paper. As the BioBERT model only processes a maximum of 512 words after tokenization, if needed, each sample was split to input text into non‐overlapping pieces which were either 512 words long or shorter. BioBERT had 12 transformer encoder layers.

BioBERT models were trained using three‐fold cross‐validation, and prediction performance was based on their test samples. The prediction performance metrics are accuracy, precision, recall, and F1‐score. The initialized model was fine‐tuned by training with a low learning rate (2e‐5). The epoch with the best performance in the test set for the large‐scale prediction within 10 epochs was saved. Based on our experience, models typically converge within 10 epochs in the model comparison phase.

Because of the cross‐validation, each sample served as a test sample and has a predicted label. Therefore, when comparing the predicted labels to the true labels, all the misclassified samples were identified. These misclassified labels were subjected to manual review and relabeled if needed. This label validation step is called calibration.

All abstracts passing the second PubMed query were predicted by three BioBERT models and generated three predicted labels on PK, PE, and CT. Any abstract predicted as positive in at least one study type was included in MPRINT‐KP Silver. All data processing, model construction, and analysis were finished with Python 3.7 on the Ohio Supercomputer Center (OSC). Large language model construction and prediction utilized the Pitzer 48 core GPU with CUDA 10.1.168.

### 2.4 MPRINT‐KP Silver PubMed Abstract Annotation

Drug names mentioned in an abstract relied on an extensively controlled vocabulary derived from drug‐related terms within the Unified Medical Language System (UMLS) Meta‐thesaurus [10]. Our drug vocabulary capitalizes on UMLS's rich semantic network, including pharmaceuticals, biologics, and nutrients to standardized terminologies. A filtering step was implemented to mitigate the issues of short and ambiguous synonyms and to enhance precision. Short drug synonyms (fewer than five characters) were excluded from the vocabulary used to scan the text. This dictionary was then applied to scan the titles, abstracts, keywords, and Medical Subject Headings (MeSH) heading terms associated with each PubMed abstract.

Disease names were derived from MeSH associated with each PubMed abstract. Specifically, disease‐related terminology was defined utilizing the MeSH Tree Structure—encompassing all terms under C (Disease) and F (Psychiatry and Psychology). Study type was predicted by the BioBERT models, including PK, PE, and CT. Population was annotated with a curated list of specific population groups with synonyms (Table S3). These relevant terms (e.g., “children,” “pregnancy,” and “lactation”) were used to scan the abstract, the title, and MeSH heading terms.

### 2.5 MPRINT‐KP Silver Database

The database is implemented based on MySQL 8.0 in a Microsoft Azure environment to facilitate data management (Figure S1). All knowledge in MPRINT‐KP Silver is organized in a tabular format and associated with a relational model. The MPRINT‐KP Silver database houses a comprehensive collection of relevant publications, each uniquely identified by its PubMed ID (pmid). The publication table captures essential metadata directly from PubMed, including the publication's title, abstract, and publication year. The database also includes several annotation tables, namely *pmid2drug*, *pmid2disease*, *pmid2studyttype*, and *pmid2pop*, which are specific to each PubMed abstract. All these tables are connected in the common identifier, pmid. Notes S1 and S2, Figure S2 show more details in other data sources used in MPRINT‐KP Silver and the MPRINT‐KP Silver user interface, respectively.

### 2.6 Medication Prescription Rate Analysis

The Merative MarketScan [11] commercial claims database was utilized to identify pregnant and pediatric participants between 2016 and 2021. Our analysis included health care utilization and expenditures related to outpatient and a limited number of inpatient claims received by insurance companies. Pregnant participants were included if they had at least one delivery and/or procedure code (Table S4). The date associated with the first delivery code was defined as the index date, and only those participants that were continuously enrolled for 270 days before and 180 days after the index date were included within the pregnant or postpartum subpopulations. Any drug‐related prescriptions within 270 days before the index date were counted in the pregnancy subpopulation. Prescriptions from the index date to 180 days after the index date were counted within the postpartum period. Pediatric sample size and medication prescription rates were calculated separately for three age subgroups: neonates/infants (0–1 years), children (1–12 years), and adolescents (12–18 years). Pediatric sample size in each subgroup was cumulative across 5 years of MarketScan data (2016–2021). Use of a medication by a pediatric patient was counted in his/her proper age subgroup in each year, and the overall prescription rate was the sum up of medication frequency across all 5 years, 2016–2021. In both pregnancy and pediatric subpopulations, the medication prescription rates were calculated as the overall medication prescriptions divided by the corresponding sample size of the respective subpopulation.

Drug names were processed before calculating medication prescription rate. Multi‐ingredient drugs were decomposed into individual ingredients, and redundancy was removed. Then in the single ingredient drug list, allergens, extracts, vaccines, prenatal vitamins, lotion, and kit were removed. The remaining drug names were further filtered by the source names (e.g., human, bovine), manufacturing process/molecular modification (e.g., micronized, injection), and formulation (e.g., hydrochloride, calcium) (Table S5).

### 2.7 Drug Publication Frequency Calculation

Drug publication frequencies were calculated by counting the number of abstracts that contain this drug generic name and all its synonyms. The abstract includes the title, abstract text, and MeSH terms. The synonym list was generated through a search result of the UMLS with the application programming interface (API) [10]. The synonym list was further cleaned by removing noisy patterns, such as dosage and duplicates.

### 2.8 Pharmacovigilance Analysis of Top Prescribed Drugs Using Real World Data

Two different real world data sources were used for pharmacovigilance analysis of top prescribed drugs that have limited pharmacology publications. For pediatric patients between 0 and 2 years old, the United States Food and Drug Administration Adverse Event Reporting System (FAERS) was chosen, as it contains both pediatric patients and their reported adverse drug events (ADEs). Among the top 100 prescribed drugs, 10 drugs with the least pharmacology publication evidence were selected for pharmacovigilance analysis using FAERS between 2004Q1 and 2025Q2. It has 114,694 pediatric case reports in patients who were aged 0–2 years old. Drug‐associated ADEs were analyzed using four well recognized methods: Proportional Reporting Ratio (PRR) [12], Reporting Odds Ratio (ROR) [13], Information Component (IC) [14], and the Empirical Bayes Geometric Mean (EBGM) [15]. Top drug associated ADE pairs were selected based on the threshold of lower bounds of four statistics: IC025 > 0, PRR025 > 1, ROR025 > 1 and EB05 > 1.

In pharmacovigilance analysis for pregnant women, because FAERS does not have annotated data in pregnant women, Merative MarketScan between 2016 and 2021 was chosen as the real world data source. Among the top 100 prescribed drugs, 10 drugs with the least pharmacology publication evidence were selected for pharmacovigilance analysis using MarketScan data. It has claims data for 1,048,726 pregnant women. They were defined as having at least one delivery and/or procedure code (Table S4). Figure S3 illustrates the pharmacoepidemiology nested case control study design. Cases were defined as pregnant women taking the drug, while the controls were not taking the drug. The drug exposure window of 2 months was within the pregnancy period. Only the International Classification of Diseases, 10th Revision (ICD10) codes reported within the drug exposure window were considered. The association between drug exposure and ICD10 codes was tested through a chi‐square test. P‐value threshold was set at 0.00001 for the multiple comparison consideration. As not all of the ICD10 codes are ADEs, we purposefully removed ICD10 codes related to pregnancy‐related diseases and drug indication‐related diseases and symptoms.

## 3 Results

### 3.1 Screening Maternal and Pediatric Pharmacotherapy Relevant Papers

**FIGURE 2:**
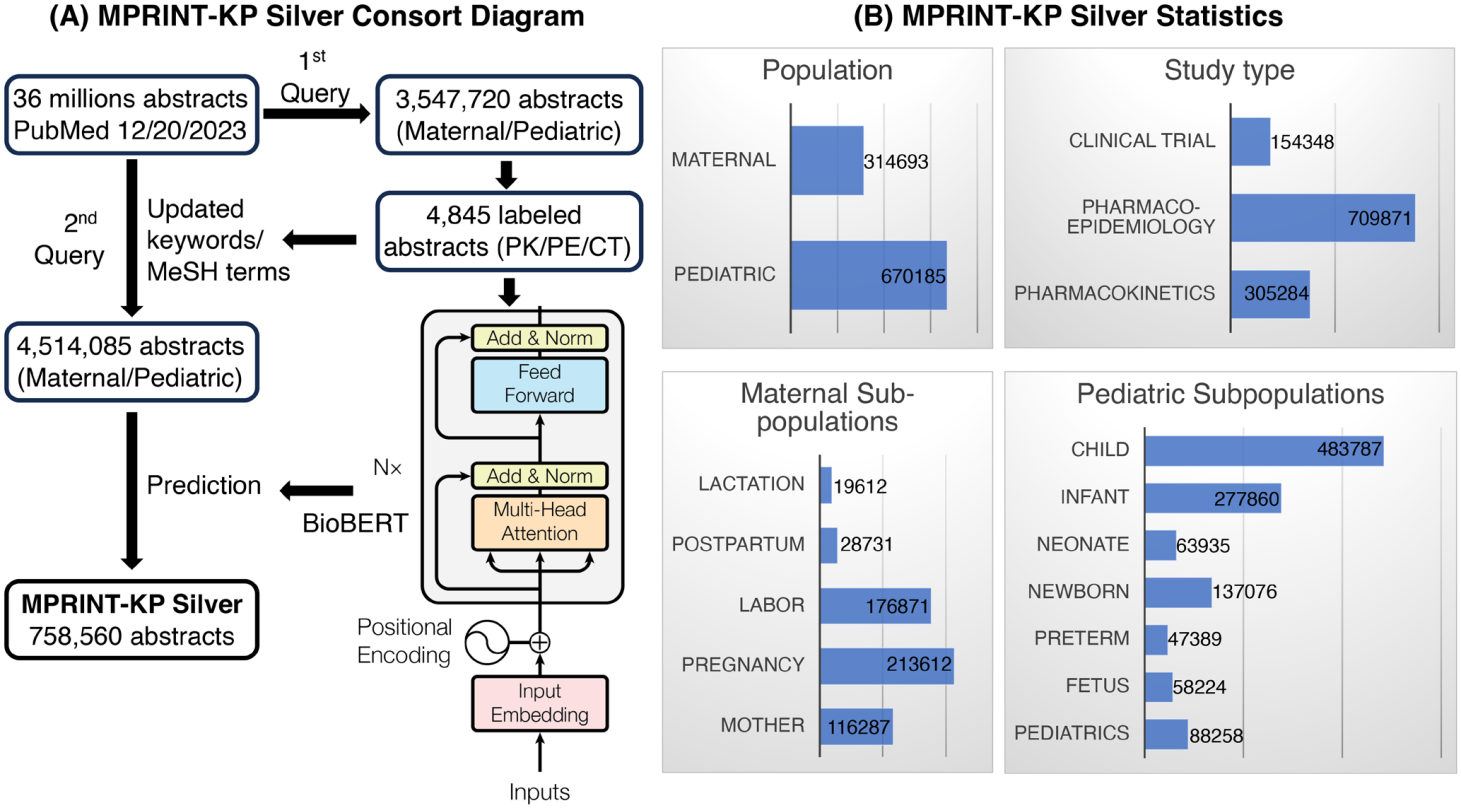
MPRINT‐KP Silver development (A) The consort diagram of MPRINT‐KP Silver development [8]. (B) MPRINT‐KP Silver database statistics in study type and subpopulations in maternal and pediatric patient populations. BioBERT, bidirectional encoder representations from transformers for biomedical text mining; CT, clinical trial; MeSH, medical subject heading; MPRINT‐KP silver, maternal and pediatric precision in therapeutics knowledge portal; Norm: normalization; PE, pharmaco‐epidemiology; PK, pharmacokinetics.

Using the initial keyword lists (Table S1), the first query screened 36,555,430 PubMed citations published by the National Library of Medicine (NLM) on December 20, 2023, resulting in 3,547,720 maternal or pediatric pharmacotherapy‐related abstracts. A random subset of 4845 abstracts was selected for manual annotation. After filtering out 2067 unrelated abstracts, MeSH terms from the related abstracts were collected and integrated into a second query on December 10, 2024. This query identified 4,514,085 abstracts (Figure 2A). The PMID, title, abstract, MeSH term, publication type, keyword, language, and date completed were collected for further analysis.

### 3.2 Performance of BioBERT Models and Other Models

A total of 4845 abstracts were manually labeled for the large language model training and evaluation task. Among them, 2067 abstracts were not relevant to maternal/pediatric pharmacotherapy research and labeled as negative. Of the remaining 2778 abstracts, 1363 were labeled PE only, 512 were CT only, 269 were PK only, 588 were PK and PE, and 46 were PK and CT. During the double labeling process, a 72.16% agreement rate was achieved. Inconsistent labels were reviewed by a third expert.

**FIGURE 3:**
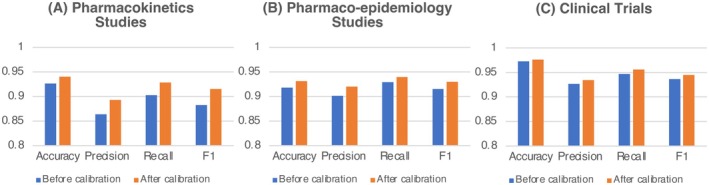
BioBERT model prediction performance in classifying pharmacokinetics studies (A), pharmacoepidemiology studies (B), and clinical trials (C). The prediction performance is evaluated in accuracy, precision, recall, and F1‐score. BioBERT model prediction performance improved after sample calibration. F1, F1‐score.

BioBERT model prediction performance is shown in Figure 3. The overall performance, measured by the F1 score, among three BioBERT models was all above 90%. CT classification by the BioBERT model had the best performance with an F1‐score close to 95%. The BioBERT model prediction F1‐score for PK and PE studies was 91.5% and 92.5%, respectively. These models all had better recall than precision. In other words, BioBERT models were more likely to correctly identify true PK, PE, and CT abstracts than unrelated ones. Sample calibration among misclassified abstracts improved BioBERT model performance by 1% to 3%.

### 3.3 MPTINT‐KP Silver

The optimal BioBERT‐base model was implemented to classify PE, CT and PK abstracts from 4,514,085 papers after the second PubMed query. It generated labeled abstracts for 154,348 CT, 709,871 PE, and 305,284 PK studies; 670,185 abstracts were identified for pediatric patients and 314,693 for maternal patients (Figure 2B). Among maternal abstracts, more studies were identified relating to pregnancy (213,612) and labor/delivery (176,871) than lactation (19,612) or postpartum (28,731). “Mother” is a general category that incorporated combinations of multiple subpopulations. Among pediatric subpopulations, there are more publications on children (483,787) and infants (277,860) than other age groups (i.e., neonate (63,935), newborn (137,076), preterm (47,389) and fetus (58,224)). “Pediatric” is a general category that is typically a combination of several pediatric subpopulations.

### 3.4 Pharmacology Knowledge Landscape and Gap Analysis

**FIGURE 4:**
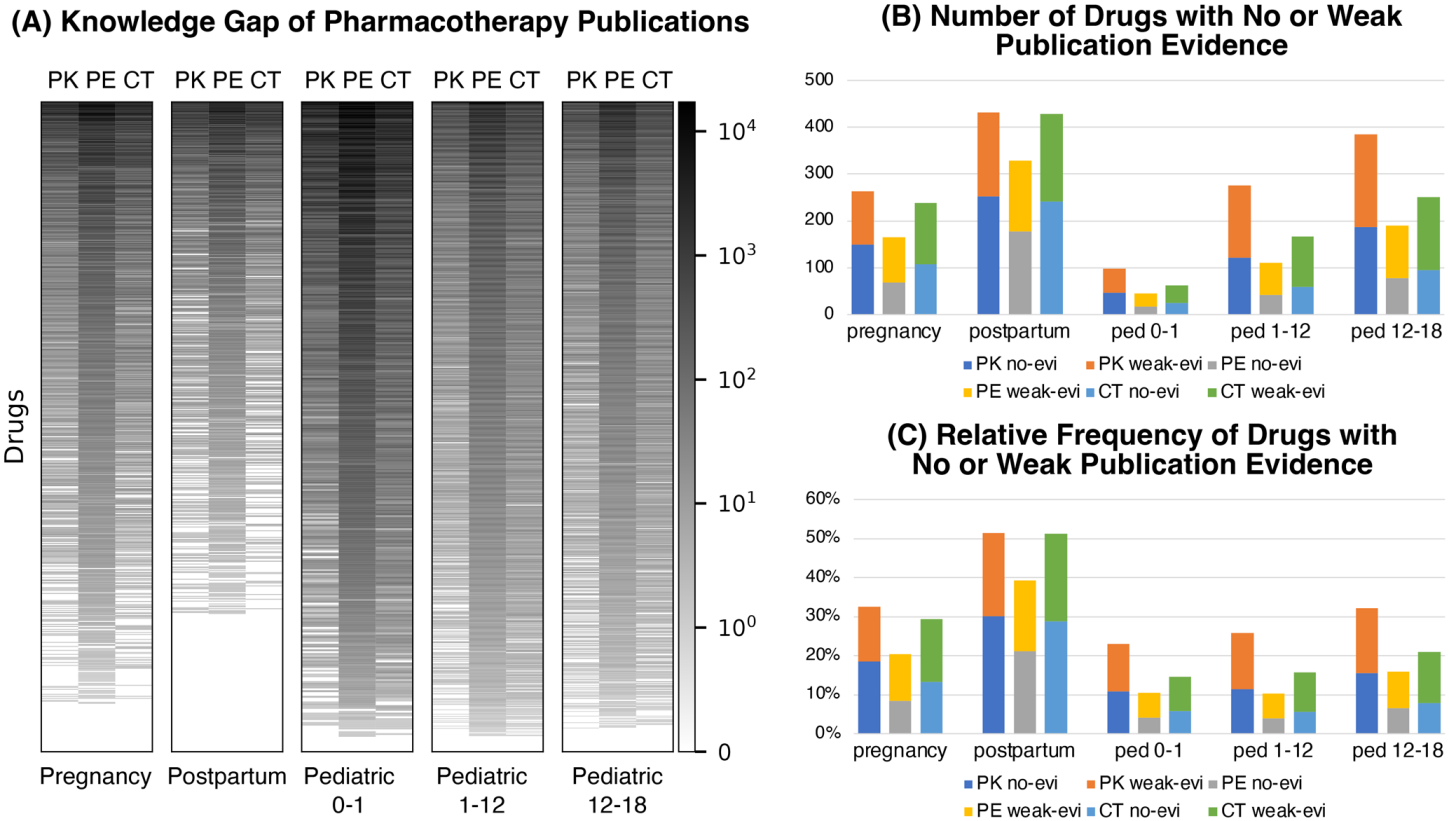
Pharmacology research knowledge gap landscape analysis. (A) Heatmap of publication frequencies in PK, PE, and CT among five patient subpopulations: Pregnant women, postpartum women, and pediatric patients in age groups 0–1, 1–12, and 12–18 years old. (B) The number of drugs with no or weak pharmacology research evidence in five patient subpopulations and each study type. (C) The percentage of drugs with no or weak pharmacology research evidence in five patient subpopulations and each study type. The weak evidence is defined as less than five publications. CT, clinical trial; no‐evi, no evidence; PE, pharmacoepidemiology; ped, pediatric; PK, pharmacokinetics; weak‐evi, weak evidence.

Medication prescription analysis was performed using MarketScan data for five sub‐populations: pregnancy, postpartum, and pediatric age groups (0–1, 1–12, and 12–18 years). Only drugs with more than 10 prescriptions across 5 years of data were included. Figure 4A shows the landscape analysis (i.e., heatmap) of PK, PE, and CT publication frequencies of drugs prescribed to each sub‐population ranked in descending order of overall publication frequency in MPRINT‐KP Silver. The publication frequency data are available in the Table S6. The number of drugs and their relative frequencies that have no publication or weak evidence, defined as less than five papers, are displayed in Figure 4B,C, respectively. Of the 856 unique drugs prescribed to postpartum women, 40% to 50% were lacking either PK, PE, or CT evidence. During pregnancy, 828 unique drugs were prescribed, with 20% to 33% having no or limited PK, PE, or CT publication evidence. All pediatric age groups exhibited a higher percentage of PK, PE, or CT evidence (11%–33%). However, the number of unique drugs prescribed for neonates was much lower than other populations, with only 440 unique medications used. Children 1–12 and > 12 years of age had 1085 and 1224 unique medications prescribed, respectively. As shown in Figure 4B,C, we observe that PK data generally has the highest knowledge gap (i.e., more drugs with no or weak publication evidence) than PE and CT studies, and PE has the least knowledge gap.

**FIGURE 5:**
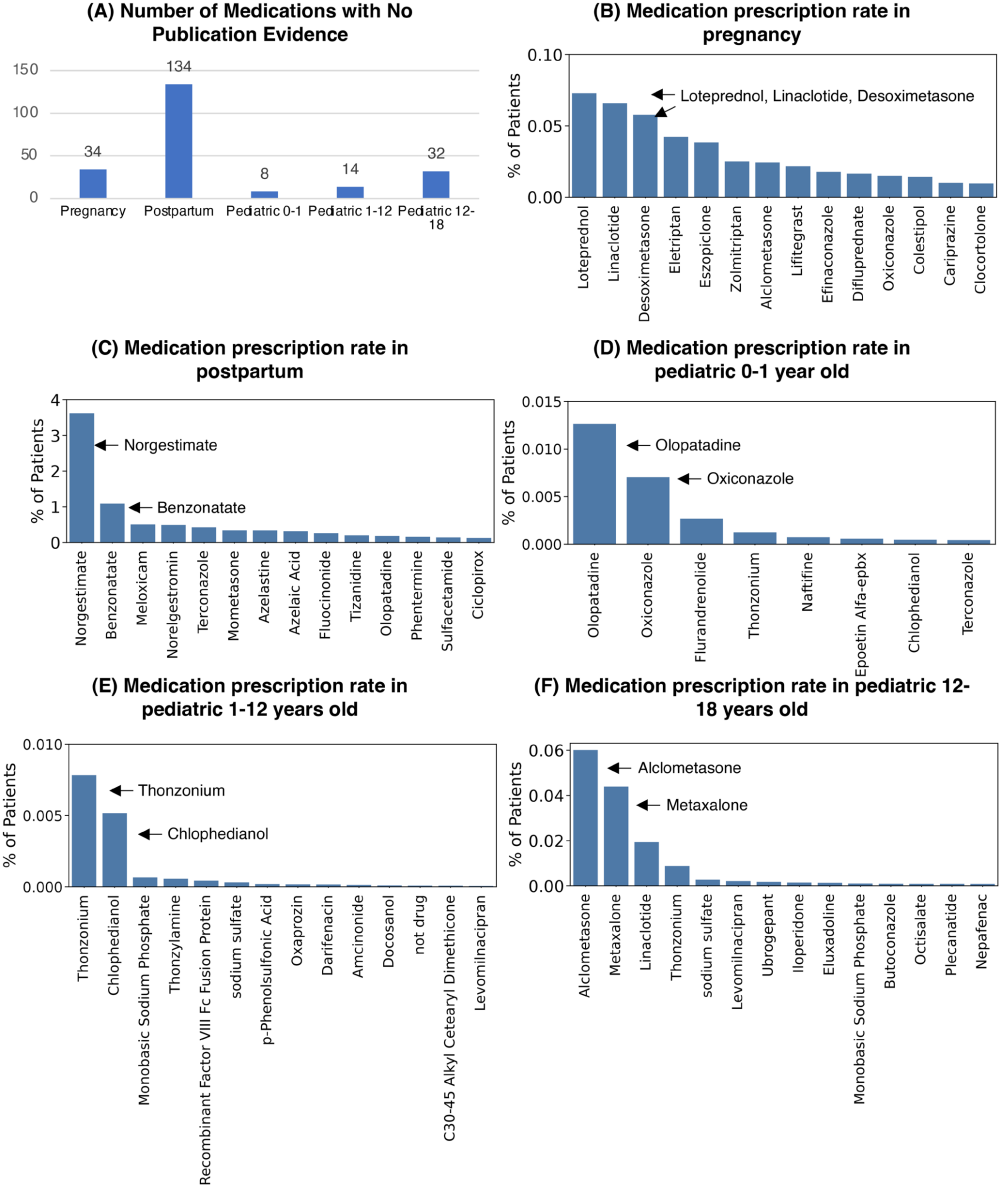
Medication prescription rates for drugs without pharmacology research evidence. (A) The number of drugs without PubMed evidence in all three pharmacology study types. Medication prescription frequencies are ranked for those drugs without pharmacology research evidence in five subpopulations, namely pregnant women (B), postpartum women (C), pediatric 0–1 years (D), pediatric 1–12 years (E), and pediatric 12–18 years (F). Top ranked drugs are highlighted in the figure.

Analysis of the MPRINT‐KP Silver revealed that 34, 134, 8, 14, and 32 prescribed drugs did not have any publications (PK, CT, or PE) in pregnancy, postpartum, 0–1 y, 1–12 y, and 12–18 y, respectively (Figure 5A). Figure 5B–F and Table S7 rank these drugs based on the number of individuals prescribed each drug to demonstrate the knowledge gap. Here we select a few top prescribed drugs in each subpopulation as examples.

In pregnant women, loteprednol [16], linaclotide [17], and desoximetasone [18] are the top three drugs without published pharmacology evidence. Loteprednol is an ophthalmic corticosteroid that is used to treat redness, itching, and watering of the eyes caused by allergies, infection, or eye surgery. It may also be used short‐term to treat dry eye. Linaclotide is a guanylate cyclase‐C (G‐CC) agonist indicated for treatment of irritable bowel syndrome with constipation and chronic idiopathic constipation [19]. Diarrhea occurs in over 10% of individuals taking linaclotide, and it has a black box warning regarding use in children < 6 years of age due to severe dehydration. Although no publications were found on linaclotide's safety in pregnancy, maternal dehydration may lead to lower levels of amniotic fluid and fetal complications. Desoximetasone is a topical corticosteroid that relieves inflammation on pruritic symptoms of dermatoses and treats plaque psoriasis. Although desoximetasone is applied to the skin, significant systemic absorption may occur if used over a large area or with occlusive coverings.

Among the postpartum women, the top two drugs without evidence are norgestimate [20] and benzonatate [21]. Norgestimate is a synthetic progestin marketed as a combined hormonal contraceptive with ethinyl estradiol or estradiol. Although there is a gap of research in the postpartum women, guidelines caution against use of combined hormonal contraceptives in the postpartum period due to increased risk of venous thromboembolism [22]. Benzonatate is an oral antitussive. Central nervous system (CNS) adverse effects including confusion and hallucinations are common.

Among infants, pediatric patients 0–1 year old, olopatadine [23] and oxiconazole [24] are the top two drugs without evidence. Olopatadine is an ophthalmic antihistamine used to treat eye itching caused by allergies. Oxiconazole is a topical antifungal indicated for skin infections such as jock itch and ringworm. Thonzonium [25] and chlophedianol [26, 27] are the top two prescribed drugs in pediatric patients between 1 and 12 years old with limited information. Thonzonium is a cationic surfactant widely used in ear and nasal drops to enhance dispersion and penetration of cellular debris and exudate, thereby promoting tissue contact of co‐administered medications. Often found in combination products, chlophedianol is used to relieve dry and irritating coughs. Its common side effects include hallucinations, skin rash, and blurred vision. Among 12–18 year old pediatric patients, alclometasone [28] and metaxalone [29] are the top two drugs without evidence. Alclometasone is a topical corticosteroid used to relieve inflammation and itching by various skin conditions. It may cause skin reactions such as irritation or rash. Metaxalone is a skeletal muscle relaxant with an unclear mechanism of action. It can cause CNS depression, leading to side effects such as drowsiness or headache, as well as hematologic issues.

### 3.5 Adverse Drug Events Discovery Using Real World Data for Highly Prescribed Drugs With Some or Limited Pharmaco‐Epidemiology Evidence

In the pediatric patient population, after applying four different pharmacovigilance methods in FAERS, PRR, ROR, IC, and EBGM with prespecified lower 2.5% confidence thresholds, two drugs showed statistically significant associated ADEs. Their prescription frequency ranges between 1.4 and 6.2 per 1000 persons. Table 2 reported those new ADEs that are not overlapped with their drug labels, regardless of the general adult population or pediatric population, nor published in the literature. Crisaborole, a nonsteroidal topical ointment used to treat mild‐to‐moderate eczema in children, was associated with hemorrhage. Moxifloxacin is prescribed to treat bacterial infections. Its associated ADEs include abdominal distension, cardiac arrest, respiratory arrest, fetal heart rate deceleration abnormality, and among others.

**TABLE 2:** Drug associated adverse events identified from real world data.

Drugs	Prescription frequency in 1000 persons	Significant ADEs in pharmacovigilance analysis but not reported in the literature or drug labels
*Pediatric population (0–2 years, FAERS)*		
Crisaborole	1.4	Hemorrhage.
Moxifloxacin	6.2	Abdominal distension, cardiac arrest, respiratory arrest, edema peripheral, dyskinesia and fetal heart rate deceleration abnormality.
*Pregnant women population (MarketScan)*		
Terconazole	43.5	Abnormalities of heartbeat, iron deficiency anemia, abnormalities of breathing, headache, dizziness and giddiness, pain in throat and chest, cough, elevated blood glucose level, visual disturbances, asthma, and disorders of white blood cells.
Benzonatate	9.8	Headache, pain unspecified, bipolar disorder, major depressive disorder, other anxiety disorders, and reaction to severe stress and adjustment disorders.
Buspirone	6.2	Deficiency of vitamin B and D, asthma, irritable bowel syndrome, abnormalities of heartbeat, and abnormalities of breathing.
Cyclobenzaprine	18.5	Tubulo‐interstitial nephritis.
Pantoprazole	16.2	Conductive and sensorineural hearing loss, diseases of salivary glands, abnormal involuntary movements, and voice and resonance disorders.

Abbreviations: ADEs, adverse drug events; FAERS: United States Food and Drug Administration Adverse Event Reporting System.

In pregnant women, a nested case control study was implemented to screen the drug‐associated ICD10 codes in MarketScan data (Figure S3). Unlike overlapping four pharmacovigilance analysis methods in FAERS and their 2.5% confidence thresholds, a Chi‐square test of a 2 by 2 table was conducted to screen top drug associated ICD10 codes, in which P‐value threshold was set as 0.00001. After removing ICD10 codes related to drug indications or pregnancy related diseases or symptoms, we further filtered ICD10‐related symptoms that were also reported in the drug labels, including both general adult population and pregnant women. In addition, those statistically significant ICD10 symptoms were filtered if they were reported in the literature. As a result, a number of new ADEs were found associated with five drugs (Table 2). Their prescription frequency ranges from 6.2 to 43.5 per 1000 persons. Terconazole is an antifungal medication used to treat vaginal yeast infections. It had the highest unreported associated ADEs, including abnormalities of heartbeat, iron deficiency anemia, abnormalities of breathing, elevated blood glucose level, visual disturbances, asthma, and among others. Benzonatate is prescribed as a non‐narcotic cough symptomatic relief medicine. Its new associated ADEs included headache, bipolar disorder, major depressive disorder, and some other anxiety disorders. Buspirone is an anti‐anxiety medication, which showed associated deficiency of vitamin B and D, asthma, irritable bowel syndrome, abnormalities of heartbeat, and abnormalities of breathing. Cyclobenzaprine is a prescription skeletal muscle relaxant that is used as an adjunct to physical therapy to relieve muscle spasms. The newly reported ADE was tubulo‐interstitial nephritis. Pantoprazole is a proton pump inhibitor medication that reduces the amount of acid produced in the stomach. In pregnant women, its new ADEs included conductive and sensorineural hearing loss, abnormal involuntary movements, voice and resonance disorders, and among others.

## 4 Discussion

The MPRINT‐KP provides a comprehensive overview of PK, PE, and CT evidence in maternal and pediatric populations. The MPRINT‐KP Silver facilitates identification of all relevant research publications, as well as gaps in knowledge relating to therapeutics in maternal and pediatric populations. The landscape analysis revealed a number of drugs that have no or weak evidence relating to PK, PE, and CT outcomes among pregnancy, postpartum, and three different pediatric patient populations of 0–1, 1–12, and 12–18 years of age (Figure 4). The relative frequencies of drugs with no or weak publication evidence are as high as 40% to 50% in postpartum women. Although the BPCA and other initiatives have improved research in pediatric patients, minimal evidence regarding PK, safety, and efficacy was still found for 10%–25% of drugs used in children.

Several drugs found to be used in individual patient populations had little pharmacology research evidence. Many of these drugs are not systemically administered, such as the topical corticosteroids, desoximetasone and alclometasone; antifungal oxiconazole; ophthalmic agents such as loteprednol and olopatadine; and the thonzonium eardrop. Although many of these agents are considered to be locally active, changes in skin permeability, particularly in young children, may increase the risk of systemic absorption. Overall, there are limited data on safety, efficacy, and optimal dosing of topical agents in these populations, indicating a critical gap in data. Other drugs found to be prescribed in these populations with no supportive literature data may be associated with increased risk of adverse events, such as the increased risk of thromboembolic events with norgestimate in the postpartum period, linaclotide's risk of dehydration in pregnancy, and CNS side effects of benzonatate and metaxalone in pediatric populations.

Pharmacovigilance analyses were performed for top ranked prescribed drugs in pediatric and pregnant women patient populations. Comparing to literature and drug label data, a number of new ADEs were found in some drugs. In pediatric patients 0–2 years old, we found crisaborole‐associated hemorrhage and moxifloxacin‐associated cardiac toxicities. In pregnant women, we identified terconazole‐associated abnormalities of heartbeat and iron deficiency anemia; benzonatate‐associated depressive disorder and anxiety disorders; buspirone‐associated deficiency of vitamin B and D, and abnormalities of heartbeat and breathing; cyclobenzaprine‐associated tubulo‐interstitial nephritis; and pantoprazole‐associated hearing loss and voice and resonance disorders. Using this similar approach (i.e., integrating MPRINT‐KP Silver and real world data), we recently published benzonatate‐associated ADEs, including seizure, death, and arrhythmia [7].

MPRINT‐KP Silver was generated using MPRINT‐KP BioBERT models, and its development significantly benefited from current artificial intelligence (AI) technology. BioBERT models were able to achieve 91% or above F‐scores in classifying PK, PE, and CT abstracts. In addition, their recall rates were 93% or above. In other words, if a drug has a pharmacology research paper in pediatric or maternal patient populations, the BioBERT model has a 93% chance of identifying its abstract. In our research landscape analysis, a drug with less than five papers was defined as weak evidence. The MPRINT‐KP BioBERT model has a 0.07^4^ = 0.0024% probability of missing the weak evidence for a drug. Therefore, we are highly confident that MPRINT‐KP Silver has captured almost all clinical pharmacology research papers in maternal and pediatric populations from PubMed. Thus, the follow‐up knowledge gap analysis on PK, PE, and CT evidence in maternal and pediatric subpopulations is highly accurate.

There are remaining challenges in MPRINT‐KP to enhance its utility in pharmacology research, education, and regulatory communities. The first challenge is the exclusion of other publication databases, such as Web of Science or Scopus. This is due to the fact that these databases have limited accessibility. For instance, Scopus has a limit of 20,000 abstracts per download and Web of Science has a limit of 1000 citations per download. This significantly limits our global analysis of publication trends and gaps in maternal and pediatric pharmacology research.

The second challenge is to find a more in‐depth knowledge gap in pharmacology therapy and conduct corresponding pharmacovigilance and PE studies using real world data. Although MPRINT‐KP Silver classifies PK, PE, and CT papers well, it cannot yet extract reported drug outcome variables in the literature. Hence, MPRINT‐KP Silver cannot automatically and directly compare literature pharmacotherapy efficacy and ADEs to those in drug labels and identify discrepancies or knowledge gaps. This MPRINT‐KP Silver limitation also has a practical constraint on how real‐world data can be used to fill those pharmacotherapy knowledge gaps. In this paper, we manually reviewed drug label data and literature data of the 10 top ranked prescribed drugs in either pediatric patients or pregnant women, conducted pharmacovigilance studies using real world data, and identified a number of drug associated new ADE evidence. This highly labor‐intensive manual work cannot be scaled up to cover all drugs. We anticipate more powerful AI technology can be a game changer and meet this challenge.

The third challenge is annotation of pediatric and maternal pharmacology research. In our inclusion criteria, many biomarker studies are included in our maternal and pediatric pharmacology research papers. These biomarker studies are not annotated in MPRINT‐KP Silver yet because of a lack of ontology scheme. However, they are important in maternal and pediatric therapeutics research. On the other hand, the MPRINT‐KP Silver has a solid disease ontology that integrates ICD codes and MeSH terminology through UMLS in the backend database. However, this disease ontology is not yet developed for maternal or pediatric patients.

The fourth challenge is the medication prescription rate estimate from diverse real world data types. The current analysis heavily relies on MarketScan claims data, which are primarily outpatient. MarketScan mainly represents families with higher socioeconomic status, and therefore may not be fully representative of the overall maternal and pediatric patients, particularly those covered by Medicaid or from lower‐income households.

## 5 Conclusions

Pregnant women and children have been underrepresented in clinical trials due to ethical concerns and perceived vulnerabilities. This resulted in a significant gap in knowledge regarding the safety and efficacy of medications for these populations. MPRINT‐KP is the first pharmacology knowledgebase that is designed to provide a comprehensive view of PK, PE, and CT research evidence in maternal and pediatric patient populations. Using MPRINT‐KP Silver data, the landscape analysis revealed a number of drugs that have PK, PE, and CT knowledge gaps in pregnant, postpartum, or pediatric populations of interest. Using real world data, among a number of highly prescribed drugs with limited PE evidence, pharmacovigilance analyses revealed many new drug‐associated ADEs. Pharmacology research knowledge gaps identified by MPRINT‐KP will motivate translational pharmacology research in PK studies, PE studies, and CT.

## Author Contributions

**Xiaofu Liu:** software, data curation, investigation, formal analysis, validation, visualization, writing – original draft, writing – review and editing. **Aditi Shendre:** data curation, investigation, formal analysis, validation, writing – original draft, writing – review and editing. **Lei Wang:** writing – review and editing, writing – original draft, visualization, software, data curation, investigation, validation, formal analysis. **Aislinn O'Kane:** data curation, investigation, writing – original draft, writing – review and editing, validation. **Hanson Ma:** software, validation, investigation, formal analysis. **Chien‐Wei Chiang:** validation, software, investigation, formal analysis, data curation. **Syed S. Zaidi:** data curation. **Omar A. Aboshady:** data curation. **Gerald So:** data curation. **Emma M. Tillman:** data curation. **Lindsey M. Kirkpatrick:** data curation. **Maged Costantine:** conceptualization, methodology. **Shaun Grannis:** conceptualization, methodology. **Colin M. Rogerson:** conceptualization, methodology. **Christopher Bartlett:** conceptualization, methodology. **Saurabh Rahurkar:** data curation, visualization. **Lijun Cheng:** data curation. **Jiayi Ouyang:** formal analysis. **Ping Wei:** formal analysis. **Zhimo Zhu:** software. **Shangjia Li:** data curation. **Yirui Huang:** data curation. **Lingling Wang:** data curation. **Weidan Cao:** data curation. **Haoran Jiang:** data curation. **Jianing Liu:** data curation, project administration. **Samuel‐Richard Oteng:** data curation. **Andrew Goodwin:** data curation. **Jiezel Ann Faith Deypalubos:** data curation. **Shijun Zhang:** data curation. **Robert Bies:** conceptualization, methodology. **Sara K. Quinney:** conceptualization, methodology, supervision, writing – original draft, writing – review and editing, data curation, validation, formal analysis, project administration, funding acquisition, resources. **Lang Li:** conceptualization, methodology, data curation, investigation, validation, writing – original draft, writing – review and editing, formal analysis, supervision, funding acquisition, resources, project administration.

## Funding

The research was supported by Eunice Kennedy Shriver National Institute of Child Health and Human Development (USA): P30HD106451. National Center for Advancing Translational Sciences (USA): UM1TR004548. National Library of Medicine (USA): R01LM014199. National Institute of General Medical Sciences (USA): T32GM008425. National Cancer Institute (USA): U01CA248240; K12HD111057‐03.

## Conflicts of Interest

The authors declare no conflicts of interest.

## Supporting information


**Table S1:** Maternal and pediatric keywords for PubMed query inclusion.
**Table S2:** Animal and other keywords for PubMed query exclusion.
**Table S3:** Maternal and pediatric population curated list.
**Table S4:** Delivery and/or procedure code.
**Table S5:** Drug name cleaning, including removing terms if they are extraction, vaccine, multi‐ingredient substance, or medical products (not drug), and cleaning terms by removing words represent the manufacturing/formulation descriptors and salt forms.
**Table S6:** MarketScan drug publication frequency workbook, including five sheets for maternal and pediatric subpopulations. Each sheet includes the drugs with medication frequency larger than 10, and corresponding publication frequency.
**Table S7:** MarketScan drugs with no publications in pregnancy, postpartum, 0–1, 1–12, and 12–18 years.
**Figure S1:** MPRINT‐KP silver database. (A) Silver backend database, ADMET, absorption, distribution, metabolism, and transportation; ATC, anatomical therapeutic chemical; MoA, mechanism of action. (B) Web application architecture.
**Figure S2:** Screenshots of MPRINT‐KP silver schemes.
**Figure S3:** MarketScan‐based pharmaco‐epidemiology nested case control study design.
**Note S1:** MPRINT‐KP silver database pharmacological knowledge.
**Note S2:** MPRINT‐KP silver user interface.

## Data Availability

The data supporting the findings of this study are available from multiple sources: Most data generated or analyzed during this study are included in this published article and its Supporting Information files. The labeled corpus is openly available in the GitHub repository at https://github.com/langli‐lab/mprint‐kp‐data. A portion of the data involving healthcare claims was obtained from Merative MarketScan Research Databases and is available from the third party. Restrictions apply to the availability of these data, which were used under license for the current study, and so are not publicly available. Data are available from the authors upon reasonable request and with permission of Merative MarketScan Research Databases.
