# Supplementary material for: Maternal and Pediatric Precision in Therapeutic Knowledge Portal (MPRINT‐KP): Landscape Analysis of Pharmacology Research in Maternal and Pediatric Patient Populations
Source: Pharmacotherapy. 2026 Feb 23;46(3):e70096. doi: 10.1002/phar.70096 (PMC12929201; doi:10.1002/phar.70096)
Supplement: Supplementary file 1 — Table S1: Maternal and pediatric keywords for PubMed query inclusion. Table S2: Animal and other keywords for PubMed query exclusion. Table S3: Maternal and pediatric population curated list. Table S4: Delivery and/or procedure code. Table S5: Drug name cleaning, including removing terms if they are extraction, vaccine, multi‐ingredient substance, or medical products (not drug), and cleaning terms by removing words represent the manufacturing/formulation descriptors and salt forms. Table S6: MarketScan drug publication frequency workbook, including five sheets for maternal and pediatric subpopulations. Each sheet includes the drugs with medication frequency larger than 10, and corresponding publication frequency. Table S7: MarketScan drugs with no publications in pregnancy, postpartum, 0–1, 1–12, and 12–18 years. Figure S1: MPRINT‐KP silver database. (A) Silver backend database, ADMET, absorption, distribution, metabolism, and transportation; ATC, anatomical therapeutic chemical; MoA, mechanism of action. (B) Web application architecture. Figure S2: Screenshots of MPRINT‐KP silver schemes. Figure S3: MarketScan‐based pharmaco‐epidemiology nested case control study design. Note S1: MPRINT‐KP silver database pharmacological knowledge. Note S2: MPRINT‐KP silver user interface. [file PHAR-46-0-s001.pdf]

Supplementary Table S1. Maternal and pediatric keywords for PubMed query inclusion

**STEP 1: Keywords from curators**	
Maternal:<br>‘maternal’, ‘mother’, ‘pregnancy’, ‘pregnant’, ‘postpartum’, ‘lactation’, ‘breast feeding’, ‘breastfeeding’, ‘nursing’, ‘labor’, ‘delivery’, ‘trimester’, ‘gestation’, ‘gestational’, ‘placenta’, ‘placental’, ‘parturition’, ‘childbirth’, ‘lactating’, ‘antenatal care’, ‘assisted reproductive technology’, ‘cesarean section’, ‘contraception’, ‘in vitro fertilization’, ‘induced labor’, ‘infant feeding’, ‘maternity’, ‘milk supply’, ‘miscarriage’, ‘obstetrics’, ‘perinatal’, ‘postnatal’, ‘postpartum’, ‘prenatal’, ‘stillbirth’, ‘birth’	Pediatric:<br>‘pediatric’, ‘neonate’, ‘neonatal’, ‘infant’, ‘child’, ‘newborn’, ‘preterm’, ‘premature’, ‘fetal’, ‘in utero’, ‘children’, ‘adolescent’, ‘adolescent health’, ‘formula feeding’, ‘neonatology’, ‘toddler’, ‘weaning’
**STEP 2: MeSH terms from training data**	
Maternal:<br>‘pregnancy’, ‘gestational age’, ‘pregnancy outcome’, ‘pregnancy complications’, ‘cesarean section’, ‘prenatal exposure delayed effects’, ‘breast feeding’, ‘milk, human’, ‘maternal-fetal exchange’, ‘pregnancy complications, infectious’, ‘pre-eclampsia’, ‘maternal exposure’, ‘obstetric labor, premature’, ‘labor, obstetric’, ‘mothers’, ‘placenta’, ‘pregnancy trimester, third’, ‘anesthesia, obstetrical’, ‘pregnancy trimester, first’, ‘diabetes, gestational’, ‘delivery, obstetric’, ‘infertility, female’, ‘pregnancy trimester, second’, ‘postpartum period’, ‘folic acid’, ‘amniotic fluid’, ‘fertilization in vitro’, ‘chorionic gonadotropin’, ‘prenatal care’, ‘prenatal diagnosis’, ‘oxytocin’, ‘abortion, induced’, ‘pregnancy in diabetics’, ‘maternal age’, ‘labor, induced’, ‘abortion, spontaneous’	Pediatric:<br>‘infant, newborn’, ‘child’, ‘infant’, ‘child, preschool’, ‘adolescent’, ‘infant, premature’, ‘infant, premature, diseases’, ‘birth weight’, ‘fetal blood’, ‘intensive care units, neonatal’, ‘infant, low birth weight’, ‘respiratory distress syndrome, newborn’, ‘infant, newborn, diseases’, ‘premature birth’, ‘growth & development’, ‘infant, very low birth weight’, ‘child development’, ‘neonatal abstinence syndrome’, ‘fetus’, ‘apgar score’, ‘embryology’, ‘amniotic fluid’, ‘infant, extremely premature’, ‘infant, small for gestational age’, ‘fetal diseases’, ‘umbilical cord’, ‘fetal growth retardation’

Supplementary Table S2. Animal and other keywords for PubMed query exclusion

**Animal study keywords**
'gerbil', 'guinea pig', 'lamb', 'mouse', 'mice', 'foal', 'dog', 'goat', 'rabbit', 'calf', 'zebrafish', 'piglet', 'rat', 'pig', 'cow', 'poultry', 'quail', 'squid', 'fish', 'gull', 'animal', 'gerbils', 'guinea pigs', 'lambs', 'foals', 'dogs', 'goats', 'rabbits', 'calves', 'zebrafishes', 'piglets', 'rats', 'pigs', 'cows', 'cattle', 'poultries', 'quails', 'squids', 'fishes', 'gulls', 'animals', 'alveolata', 'amoebozoa', 'choanoflagellata', 'cryptophyta', 'diplomonadida', 'euglenozoa', 'fungi', 'glaucophyta', 'haptophyta', 'mesomycetozoea', 'oxymonadida', 'parabasalidea', 'plants', 'retortamonadidae', 'rhizaria', 'rhodophyta', 'stramenopiles', 'viridiplantae'
**Other keywords**
'review', 'in-vitro', 'in vitro' (except 'in vitro fertilization')
**Keywords in 'publication type' field**
'review', 'systematic review', 'book review', 'scientific integrity review'
**Keywords in 'MeSH term' field**
'systematic reviews as topic', 'review literature as topic', 'in vitro techniques'

Supplementary Table S3. Maternal and pediatric population curated list

Subpopulation	Synonyms
Pregnant	‘pregnant’, ‘pregnancy’, ‘pregnancies’, ‘gravid’, ‘gravidity’, ‘gestation’, ‘primigravida’, ‘primip’, ‘placentation’
Labor	‘labor’, ‘labor, obstetric’, ‘birthing labor’, ‘labour’
Delivery	‘obstetric delivery’, ‘obstetric deliveries’, ‘delivery procedure’, ‘birthing procedure’, ‘pregnancy delivered’, ‘childbirth’, ‘parturition’, ‘delivery under medical care’, ‘birth procedures’, ‘delivery procedures’, ‘birthing’, ‘obstetrical delivery’
Postpartum	‘puerperium’, ‘postpartum’, ‘the period after childbirth’, ‘post pregnancy’, ‘post partum’, ‘puerperal’
Lactation	‘lactation’, ‘milk secretion’, ‘milk ejection’, ‘milk let down’
Fetus	‘fetus’, ‘fetuses’, ‘fetal’
Premature	‘preterm infant’, ‘premature infants’, ‘preterm infants’, ‘premature infant’, ‘premature babies’, ‘premature baby’, ‘preemies’, ‘premature infant human’, ‘preterm baby’, ‘preterm infant human’
Newborn	‘newborn’, ‘newborns’, ‘newborn infant’, ‘newborn infants’, ‘infant, newborn’, ‘infants, newborn’
Neonate	‘neonates’, ‘neonatal’, ‘neonate’, ‘neonatals’
Infant	‘infant’, ‘infants’, ‘baby’, ‘babies’
Child	‘child’, ‘children’, ‘child youth’, ‘childhood age person’, ‘0-11 years old’

Supplementary Table S4. Delivery and/or procedure code

**Delivery**		
ICD-10: Z37, Z37.0, Z37.1, Z38.00, Z38.0, Z38.1, Z38.2, Z38.00, Z38.01,Z37.2, Z37.3, Z37.5, Z37.6, Z37.50, Z37.51, Z37.52, Z37.53, Z37.54, Z38.3, Z38.4, Z38.5, Z38.6, Z37.60, Z37.68, Z37.69, Z38.7, Z38.8, Z38.30, Z38.31, Z38.61,Z37.9,O80		
**Cesarean Section**		
ICD-10: O82*, Z38.01	CPT Procedure: 59510, 59514, 59515, 59618, 59620, 59622	ICD-10-PCS Procedure: 10D.00Z0, 10D.00Z1, 10D.00Z2
**Vaginal**		
CPT Procedure: 59400, 59409, 59410, 59412		
**Vaginal after previous cesarean**		
CPT Procedure: 59610, 59612, 59614, 59618, 59620, 59622		

Supplementary Table S5. Drug name cleaning, including removing terms if they are extraction, vaccine, multi-ingredient substance, or medical products (not drug), and cleaning terms by removing words represent the manufacturing/formulation descriptors and salt forms.

**Remove the term if includes:**	**Clean the term by removing:**
Extraction and vaccine: ‘allergen’, ‘extract’, and ‘vaccine’<br>Multi ingredient: ‘multi’, ‘combination’, ‘prenatal vitamins’<br>Medical-related products: ‘cleanser’, ‘moisturizer’, ‘lotion’, “cream’, ‘monitor’, ‘device’, ‘placebo’, ‘ointment’, ‘capsule’, ‘syrup’, ‘gel’, ‘base’, ‘test’, ‘kit’	Manufacturing and formulation descriptors: ‘human’, ‘bovine’, ‘recombinant’, ‘micronized’, ‘modified’, ‘live’, ‘adjuvanted’, ‘dibasic’, ‘tribasic’, ‘macro’, ‘augmented’, ‘injection’, ‘microcrystalline’, ‘chelated’, ‘repository’, ‘adsorbed’, ‘purified’, ‘inactivated’, ‘monobasic’, ‘macrocrystals’, ‘extended’, ‘desiccated’<br>Salt forms: ‘HCl’, ‘hydrochloride’, ‘sulfate’, ‘phosphate’, ‘carbonate’, ‘maleate’, ‘acetate’, ‘benzoate’, ‘palmitate’, ‘stearate’, ‘propionate’, ‘lactate’, ‘gluconate’, ‘fumarate’, ‘tosylate’, ‘tartrate’, ‘succinyl’, ‘citrate’, ‘glubionate’, ‘malate’, ‘threonate’, ‘succinate’, ‘calcium’, ‘sodium’, ‘magnesium’, ‘potassium’, ‘iodine’, ‘chloride’, ‘zinc’, ‘aluminum’, ‘barium’

Supplementary Table S6. MarketScan drug publication frequency workbook, including five sheets for maternal and pediatric subpopulations. Each sheet includes the drugs with medication frequency larger than 10, and corresponding publication frequency.

Pregnancy Drug Publication Frequency						
**Original Drug Name**	**Cleaned Drug Name**	**Pregnancy frequency**	**All publication**	**PK publication**	**PE publication**	**CT publication**
Abacavir	Abacavir	34	564	248	512	90
Abatacept	Abatacept	22	17	1	17	1
Acarbose	Acarbose	98	15	7	15	3
Acebutolol	Acebutolol	126	47	30	40	12
Acetaminophen	Acetaminophen	54239	36433	10935	34428	9059
Acetazolamide	Acetazolamide	272	65	10	64	11
Acetic Acid	Acetic Acid	265	32194	14690	29927	6955
Acetic Acid Glacial	Acetic Acid Glacial	17	588	350	516	50
Acyclovir	Acyclovir	9704	412	66	400	65
Adalimumab	Adalimumab	810	133	45	127	58
Adapalene	Adapalene	689	529	232	497	103
Al Hydroxide	Al Hydroxide	50	40	13	37	25
Albendazole	Albendazole	30	250	99	214	86
Albuterol	Albuterol	30655	410	58	344	250
Alcaftadine	Alcaftadine	68	0	0	0	0
Alclometasone Dipropionate	Alclometasone	178	0	0	0	0
Alendronate	Alendronate	22	28	7	26	6
Algal Oil	Algal Oil	456	10	3	8	8
Allopurinol	Allopurinol	60	87	35	82	33
Almotriptan	Almotriptan	21	1	0	1	1
Aloe	Aloe	33	43796	18691	40797	10777
Alprazolam	Alprazolam	5549	27	9	23	3
Aluminum	Aluminum	206	277099	120172	260649	50900
Amantadine	Amantadine	24	30	1	27	9
Amiloride	Amiloride	27	199	75	177	41
Amino Acids	Amino Acids	254	277238	120205	260784	50905
Amitriptyline	Amitriptyline	1200	41	13	38	3
Amlodipine Besylate	Amlodipine Besylate	1746	63	11	61	12
Ammonium	Ammonium	144	18227	10778	16748	2795
Amoxicillin	Amoxicillin	96019	22376	8089	20832	6584
Amphetamine	Amphetamine	44	153514	56571	142458	39533
Ampicillin	Ampicillin	6927	189406	71887	177834	41093
Amylase	Amylase	100	484	318	453	28
Anastrozole	Anastrozole	16	30	9	25	16
Antibacterial	Antibacterial	91	16721	4414	16028	3259
Antipyrine	Antipyrine	96	1437	892	1327	218
APAP	APAP	626	32323	10319	30636	7201
Apixaban	Apixaban	79	18	8	14	3
Apremilast	Apremilast	27	1	0	0	1
Aprepitant	Aprepitant	16	86	27	80	13
Aripiprazole	Aripiprazole	826	40	11	39	17
Armodafinil	Armodafinil	78	11	2	11	4
Ascorbic Acid	Ascorbic Acid	97	27363	16497	25707	3624
Ascorbyl	Ascorbyl	1112	992	627	948	245
Asenapine	Asenapine	16	1	0	1	1
Aspirin	Aspirin	5885	25059	10796	23096	6423
Atazanavir	Atazanavir	31	204	67	188	58

Atenolol	Atenolol	722	83	20	80	38
Atomoxetine	Atomoxetine	163	38	1	38	20
Atorvastatin	Atorvastatin	689	1734	456	1686	701
Atovaquone	Atovaquone	127	29	3	29	8
Atropine	Atropine	386	276	50	263	111
Azathioprine	Azathioprine	458	1224	617	1114	131
Azelaic Acid	Azelaic Acid	4319	1	1	1	0
Azelastine	Azelastine	2153	14	6	11	7
Azithromycin	Azithromycin	70526	459	62	434	243
Aztreonam	Aztreonam	23	96	54	91	32
Bacitracin	Bacitracin	229	18	1	17	7
Bacitracin Zn	Bacitracin Zn	17	0	0	0	0
Baclofen	Baclofen	450	62	3	61	13
Baloxavir Marboxil	Baloxavir	25	5	0	4	4
Balsalazide Disodium	Balsalazide	90	3	1	3	2
Beclomethasone Dipropionate	Beclomethasone	1420	57	8	33	49
Benazepril	Benazepril	84	4	1	4	1
Benzocaine	Benzocaine	98	1517	840	1428	233
Benzonatate	Benzonatate	7136	1	1	1	0
Benzoyl Peroxide	Benzoyl Peroxide	2097	75	14	73	5
Benztropine Mesylate	Benztropine	40	4	2	4	0
Benzyl	Benzyl	31	20454	9989	19284	6241
Benzyl Alcohol	Benzyl Alcohol	144	3806	1194	3585	1688
Bepotastine Besilate	Bepotastine	49	1	0	1	1
Besifloxacin	Besifloxacin	74	1	0	0	1
Betaine	Betaine	54	839	649	764	146
Betamethasone Dipropionate	Betamethasone	7162	5	0	4	3
Betamethasone Valerate	Betamethasone	585	3	1	3	1
Betamethasone Ace	Betamethasone Ace	752	10	8	10	8
Betamethasone Na Phos	Betamethasone Na Phos	752	0	0	0	0
Bictegravir	Bictegravir	12	9	2	8	4
Bif	Bif	16	1195	378	1140	328
Bifidobacterium breve	Bifidobacterium breve	16	38	7	35	34
Bifidobacterium infantis	Bifidobacterium infantis	17	3270	1004	3015	191
Bimatoprost	Bimatoprost	39	1	0	1	0
Bioflavonoid	Bioflavonoid	1128	995	627	951	245
Biotin	Biotin	29368	1841	1258	1648	348
Bisacodyl	Bisacodyl	28	8160	3540	7669	1610
Bisoprolol	Bisoprolol	121	25	5	24	7
Boron	Boron	263	103	67	95	8
Brexpiprazole	Brexpiprazole	61	0	0	0	0
Brimonidine	Brimonidine	157	5	0	4	2
Bromfenac	Bromfenac	18	2	0	2	1
Bromocriptine Mesylate	Bromocriptine	449	708	315	690	141
Brompheniramine Mal	Brompheniramine Mal	1408	0	0	0	0
Budesonide	Budesonide	6029	196	17	150	149
Bumetanide	Bumetanide	20	23	9	21	9
Buprenorphine	Buprenorphine	1303	445	67	427	214
Bupropion	Bupropion	7372	71	15	65	23
Buspirone	Buspirone	4495	4	0	4	0

Butalbital	Butalbital	17173	29407	9312	28006	5997
Butoconazole Nitrate	Butoconazole	591	2	0	1	2
Butorphanol	Butorphanol	45	58	6	56	50
C1 Esterase Inhibitor	C1 Esterase Inhibitor	12	166	101	154	8
Ca	Ca	70695	276080	119703	259695	50797
Ca As	Ca As	235	95	35	91	12
Ca Ascorbate	Ca Ascorbate	2331	0	0	0	0
Ca Cl	Ca Cl	33	43	10	40	4
Ca Pantothenate	Ca Pantothenate	8907	0	0	0	0
Cabergoline	Cabergoline	1384	136	36	126	44
Caff	Caff	450	3461	1032	3332	1260
Caffeine	Caffeine	16529	48074	18076	45443	8692
Calcipotriene	Calcipotriene	236	8	2	8	3
Calcitriol	Calcitriol	150	622	390	576	64
Calcium	Calcium	1466	277127	120152	260684	50888
Canagliflozin	Canagliflozin	92	1	0	1	0
Carbamazepine	Carbamazepine	164	539	172	520	127
Carbidopa	Carbidopa	18	18	4	18	3
Carbinoxamine	Carbinoxamine	31	1	0	1	1
Cariprazine	Cariprazine	73	0	0	0	0
Carisoprodol	Carisoprodol	230	7570	3390	7057	1260
Carvedilol	Carvedilol	225	87	45	79	18
Cefaclor	Cefaclor	66	1665	649	1527	251
Cefadroxil	Cefadroxil	497	22	9	22	14
Cefdinir	Cefdinir	7660	126	69	115	26
Cefixime	Cefixime	306	59	14	57	31
Cefpodoxime Proxetil	Cefpodoxime Proxetil	629	99	53	98	56
Cefprozil	Cefprozil	379	16	11	16	13
Ceftriaxone	Ceftriaxone	264	452	80	443	103
Cefuroxime Axetil	Cefuroxime Axetil	3645	155	37	147	60
Celecoxib	Celecoxib	234	30	3	28	14
Cephalexin	Cephalexin	43585	93	25	92	47
Certolizumab Pegol	Certolizumab Pegol	278	36	10	35	14
Cetirizine	Cetirizine	744	28	1	23	16
Cetrorelix	Cetrorelix	860	141	36	135	114
Cevimeline	Cevimeline	11	0	0	0	0
Chlordiazepoxide	Chlordiazepoxide	45	1010	634	917	203
Chlorhexidine	Chlorhexidine	3534	222	21	196	151
Chloroquine	Chloroquine	24	694	139	675	197
Chlorpheniramine Polistirex	Chlorpheniramine	676	0	0	0	0
Chlorpheniramine Mal	Chlorpheniramine Mal	11	3	0	3	1
Chlorpromazine	Chlorpromazine	66	136	52	130	25
Chlorthalidone	Chlorthalidone	217	90	21	87	39
Chlorzoxazone	Chlorzoxazone	58	764	218	707	351
Cholecalc	Cholecalciferol	6575	467	406	414	214
Cholecalcif	Cholecalciferol	1229	467	406	414	214
Cholecalcife	Cholecalciferol	1209	467	406	414	214
Cholecalcifer	Cholecalciferol	121	467	406	414	214
Cholecalciferol	Cholecalciferol	66328	123174	47488	115970	22699
Cholestyramine	Cholestyramine	233	41	19	39	9
Choline	Choline	2036	151229	65051	141830	32672
Chorionic Gonadotropin	Chorionic Gonadotropin	2307	12059	6953	11517	2146
Chorionic Gonadotropin Alfa	Chorionic Gonadotropin Alfa	3141	481	343	428	48

Ciclesonide	Ciclesonide	79	5	2	4	4
Ciclopirox	Ciclopirox	716	1	0	1	1
Ciclopirox Olamine	Ciclopirox	272	1	0	1	1
Cimetidine	Cimetidine	228	78	25	71	38
Ciprofloxacin	Ciprofloxacin	6367	589	180	553	59
Citalopram Hydrobromide	Citalopram	5975	98	34	92	20
Citric Acid	Citric Acid	78	9841	3832	9037	2836
Clarithromycin	Clarithromycin	695	39816	19187	37874	4953
Clavulanate	Clavulanate	31128	709	117	687	247
Clidinium Bromide	Clidinium	19	16	4	15	3
Clindamycin	Clindamycin	22556	490	34	468	169
Clindamycin Palmitate	Clindamycin	41	490	34	468	169
Clobetasol	Clobetasol	3823	10	2	9	2
Clocortolone Pivalate	Clocortolone	70	0	0	0	0
Clomiphene	Clomiphene	4274	2182	414	2114	808
Clomipramine	Clomipramine	44	29	13	25	3
Clonazepam	Clonazepam	2803	91	22	85	12
Clonidine	Clonidine	412	267	52	254	143
Clopidogrel Hydrogen	Clopidogrel Hydrogen	52	26	3	25	3
Clorazepate Dipotassium	Clorazepate	18	6	4	5	2
Clotrimazole	Clotrimazole	6904	78	1	65	38
Cobamamide	Cobamamide	65	897	731	805	107
Cobicistat	Cobicistat	66	18	12	12	10
Cocoglycerides	Cocoglycerides	26	0	0	0	0
Codeine	Codeine	17098	3340	1022	3204	1275
Codeine Phos	Codeine Phos	646	3	0	3	2
Coenzyme Q10	Coenzyme Q10	20	104	63	99	20
Colchicine	Colchicine	72	166	21	164	37
Colesevelam	Colesevelam	88	1	0	1	0
Colestipol	Colestipol	104	0	0	0	0
Colistin Sulf	Colistin Sulf	45	0	0	0	0
Collagenase	Collagenase	11	35366	14336	32885	6701
Colloidal Sulfur	Colloidal Sulfur	27	21	2	21	3
Conjugated Estrogens	Conjugated Estrogens	89	2202	1189	1979	339
Copper	Copper	68	177618	75482	166691	30674
Cr	Cr	217	205250	94066	192367	36277
Crisaborole	Crisaborole	180	2	0	1	2
Cromolyn	Cromolyn	173	86	27	82	47
Cu	Cu	56210	175105	73874	164316	30061
Cu Oxide	Cu Oxide	62	0	0	0	0
Cu Sulf	Cu Sulf	62	0	0	0	0
Cyanocobalamin	Cyanocobalamin	3197	27531	17466	25815	3538
Cyclobenzaprine	Cyclobenzaprine	13511	6	2	6	0
Cyclopentolate	Cyclopentolate	55	28	1	26	16
Cyclosporine	Cyclosporine	382	1688	679	1579	204
Cyproheptadine	Cyproheptadine	462	26	11	22	7
Dalteparin	Dalteparin	13	81	27	80	44
Dapagliflozin Propanediol	Dapagliflozin Propanediol	114	1	0	1	1
Dapsone	Dapsone	487	20906	6514	20323	3248
Darunavir Ethanolate	Darunavir Ethanolate	30	59	32	50	30
Desipramine	Desipramine	12	76726	23788	72392	30198
Desloratadine	Desloratadine	177	9	4	7	8
Desmopressin	Desmopressin	87	181	63	176	23

Desogestrel	Desogestrel	2510	393	65	346	209
Desonide	Desonide	1055	190	18	144	146
Desoximetasone	Desoximetasone	421	0	0	0	0
Desvenlafaxine	Desvenlafaxine	543	25	15	23	8
Dexamethasone Sodium	Dexamethasone	81	25	9	21	21
Dexamethasone	Dexamethasone	5251	266807	115268	251507	49144
Dexlansoprazole	Dexlansoprazole	486	1	1	1	1
Dexmethylphenidate	Dexmethylphenidate	125	20	3	20	18
Dextroamphetamine	Dextroamphetamine	130	153	42	142	42
Dextromethorphan Hydrobromide	Dextromethorphan	13	1062	361	972	422
Dextrose	Dextrose	33	18155	11368	16998	3679
Diazepam	Diazepam	5311	54600	28066	50654	6015
Dichloralphenazone	Dichloralphenazone	175	760	216	703	351
Diclofenac	Diclofenac	1535	991	248	898	465
Diclofenac Epolamine	Diclofenac Epolamine	16	18	6	17	3
Dicloxacillin	Dicloxacillin	448	13	1	13	2
Dicyclomine	Dicyclomine	1649	56	6	53	22
Dienogest	Dienogest	34	172	41	150	107
Diflorasone Diacetate	Diflorasone	34	0	0	0	0
Difluprednate	Difluprednate	120	0	0	0	0
Digoxin	Digoxin	132	511	166	501	64
Dihydroergotamine Mesylate	Dihydroergotamine	26	0	0	0	0
Diltiazem	Diltiazem	207	20	5	14	7
Dimethyl	Dimethyl	49	275158	119058	258788	50854
Diphenhydram	Diphenhydram	50	54	13	50	16
diphenhydrAMINE	diphenhydrAMINE	98	3354	1031	3126	861
Diphenoxylate	Diphenoxylate	367	18	3	15	11
Diphtheria Toxoid	Diphtheria Toxoid	12	18554	7398	17527	2998
Disodiu	Disodiu	13	37	14	35	14
Disulfiram	Disulfiram	18	6	0	5	1
Divalproex	Divalproex	135	858	278	802	161
DM Hydrobrom	DM Hydrobrom	2888	0	0	0	0
Docosahe	Docosahexaenoic Acid	11	1095	865	1035	483
Docosahexa	Docosahexaenoic Acid	3640	1092	862	1032	483
Docosahexaeno	Docosahexaenoic Acid	795	2639	1643	2510	871
Docosahexaenoic A	Docosahexaenoic Acid	18483	1058	828	999	478
Docosahexaenoic Aci	Docosahexaenoic Acid	54	1053	824	994	476
Docosahexaenoic Acid	Docosahexaenoic Acid	26911	2627	1631	2499	871
Docosapentaenoic Acid	Docosapentaenoic Acid	181	60	59	58	5
Docusate	Docusate	740	73094	24918	68731	30199
Dolutegravir	Dolutegravir	57	98	27	95	45
Dornase Alfa	Dornase Alfa	36	14	1	13	7
Dorzolamide	Dorzolamide	78	4	0	4	0
Doxazosin Mesylate	Doxazosin	12	10	1	9	0
Doxepin	Doxepin	201	3	1	3	0
Doxycycline	Doxycycline	1239	314	32	295	86
Doxycycline Hyclate	Doxycycline Hyclate	7100	301	30	283	85
Doxylamine	Doxylamine	53254	89	10	85	36
Dronabinol	Dronabinol	15	3526	701	3306	435
Drospirenone	Drospirenone	1339	59	5	51	40
DSS	DSS	10662	234	79	220	41
Dulaglutide	Dulaglutide	134	1	1	0	0
Duloxetine	Duloxetine	2046	22	7	21	8

Dupilumab	Dupilumab	50	13	0	11	6
Econazole Nitrate	Econazole	362	1	0	1	0
Efinaconazole	Efinaconazole	130	0	0	0	0
Eico	Eico	177	613	497	579	173
Eicosap	Eicosapentaenoic Acid	1877	390	321	374	151
Eicosapentaenoic Aci	Eicosapentaenoic Acid	14	351	287	336	141
Eicosapentaenoic Acid	Eicosapentaenoic Acid	9733	35798	14544	33321	7199
Elagolix	Elagolix	15	3	0	1	2
Eletriptan Hydrobromide	Eletriptan	309	0	0	0	0
Elexacaftor	Elexacaftor	20	12	3	12	0
Eluxadoline	Eluxadoline	32	0	0	0	0
Elvitegravir	Elvitegravir	56	34	15	27	16
Empagliflozin	Empagliflozin	100	4	1	4	0
Emtricitabine	Emtricitabine	316	450	164	413	212
Enalapril	Enalapril	61	55	9	51	15
Enoxaparin	Enoxaparin	9502	324	90	315	132
Epinastine	Epinastine	64	0	0	0	0
Epinephrine	Epinephrine	2036	1960	840	1818	638
Erenumab-aooe	Erenumab-aooe	54	6	0	6	2
Ergocalciferol	Ergocalciferol	7593	4355	3514	3919	801
Erythromycin	Erythromycin	5818	44202	20784	41906	5758
Erythromycin Ethylsuccinate	Erythromycin Ethylsuccinate	49	4154	1620	3825	671
Escitalopram Oxalate	Escitalopram Oxalate	10281	45	13	42	11
Esomeprazole	Esomeprazole	2614	96	20	90	38
Estradiol Valerate	Estradiol	1488	7446	4094	6815	2293
Estradiol	Estradiol	23730	277020	120141	260580	50894
Estradiol V	Estradiol V	34	292	131	263	135
Estrogen	Estrogen	179	277051	120141	260607	50897
Eszopiclone	Eszopiclone	280	0	0	0	0
Etanercept	Etanercept	223	108	21	105	44
Ethinyl	Ethinyl	34	18029	6983	16922	4885
Ethinyl Estrad	Ethinyl Estrad	57	1139	316	1020	625
Ethinyl Estradiol	Ethinyl Estradiol	23360	1402	391	1268	721
Ethosuximide	Ethosuximide	11	24	14	22	2
Ethyl Oleate	Ethyl Oleate	139	15	14	12	0
Ethynodiol Diacetate	Ethynodiol	196	72	21	69	28
Etodolac	Etodolac	116	4	2	4	1
Etonogestrel	Etonogestrel	1717	152	24	141	64
Exenatide	Exenatide	27	9	1	8	2
Ezetimibe	Ezetimibe	37	23	13	21	6
Famciclovir	Famciclovir	242	8	2	7	1
Famotidine	Famotidine	9114	667	178	620	389
Fat Emulsion	Fat Emulsion	264	280	191	270	194
Fe Pentacarbonyl	Fe	48	0	0	0	0
Fe	Fe	46393	271334	117706	255260	50507
Fe Pentac	Fe Pentac	54	0	0	0	0
Fe Polysaccharid	Fe Polysaccharide	16	18983	11659	17980	1969
Fe Polysaccharide	Fe Polysaccharide	391	18983	11659	17980	1969
Felodipine	Felodipine	14	2	0	1	1
Fenofibrate	Fenofibrate	148	21	12	21	3
Fenoprofen	Fenoprofen	21	4	1	3	3
Fentanyl	Fentanyl	49	2217	200	2045	1569
Ferrous	Ferrous	1914	24017	14955	22596	3145
Ferrous Asparto G	Ferrous Asparto G	2585	1	1	1	1

Ferrous Asparto Glycinate	Ferrous Asparto Glycinate	34	3	2	3	2
Ferrous Bisglycin	Ferrous Bisglycin	11	4	2	2	4
Ferrous Fu	Ferrous Fu	403	41	22	39	35
Ferrous Fum	Ferrous Fum	4224	41	22	39	35
Fexofenadine	Fexofenadine	94	21	5	21	10
Filgrastim	Filgrastim	17	273	150	257	86
Fingolimod	Fingolimod	32	40	3	38	8
Flavoxate	Flavoxate	14	0	0	0	0
Flecainide	Flecainide	79	97	19	95	15
Fluconazole	Fluconazole	36219	334	39	327	90
Fludrocortisone	Fludrocortisone	140	70	23	69	4
Flunisolide	Flunisolide	55	4	1	3	4
Fluocinolone Acetonide	Fluocinolone Acetonide	782	10	1	9	4
Fluocinonide	Fluocinonide	1499	1	0	1	0
Fluorometholone	Fluorometholone	229	1	0	1	0
Fluorouracil	Fluorouracil	25	411	136	389	60
Fluoxetine	Fluoxetine	7654	239	71	220	61
Flurandrenolide	Flurandrenolide	77	1	0	1	0
Flurbiprofen	Flurbiprofen	17	16	2	12	14
Fluticasone	Fluticasone	20197	124	28	83	98
Fluticasone Furoate	Fluticasone	786	11	5	5	11
Fluvoxamine	Fluvoxamine	171	24	8	22	6
Fo	Fo	597	255129	109775	239627	48441
Fol	Fol	37	85639	33845	80555	19732
Folate Combinat	Folate Combinat	179	1	0	0	1
Foli	Foli	188	4472	1762	4319	1002
Folic	Folic	2808	19912	9656	18863	5256
Folic A	Folic A	4644	9243	4953	8747	1952
Folic Ac	Folic Ac	7405	4141	1641	4003	942
Folic Acid	Folic Acid	63249	19889	9652	18841	5251
Follicle Stimulating Hormone	Follicle Stimulating Hormone	2017	8322	3300	7808	2750
Follitropin Alfa	Follitropin Alfa	1978	274	93	259	133
Follitropin Beta	Follitropin Beta	1068	36	11	32	27
Fondaparinux	Fondaparinux	39	28	2	28	6
Formoterol	Formoterol	2187	528	219	468	197
Forskohlii	Forskohlii	26	0	0	0	0
Fosfomycin Tromethamine	Fosfomycin Tromethamine	326	56	8	50	15
Fremanezumab-vfrm	Fremanezumab-vfrm	32	3	0	3	1
Frovatriptan	Frovatriptan	46	0	0	0	0
Furosemide	Furosemide	423	392	125	380	113
Gabapentin	Gabapentin	2110	84	13	80	30
Galcanezumab-gnlm	Galcanezumab-gnlm	63	3	0	2	2
Ganciclovir	Ganciclovir	34	225	76	215	42
Ganirelix	Ganirelix	871	81	26	77	67
Gatifloxacin	Gatifloxacin	88	12	3	9	2
Gemfibrozil	Gemfibrozil	34	50	10	49	4
Gentamicin	Gentamicin	950	875	274	848	282
GG	GG	36	102426	46506	95530	17254
Ginger Ext	Ginger Ext	239	3	0	2	3
Glatiramer	Glatiramer	213	53	2	52	16
Glimepiride	Glimepiride	116	2	1	1	0
Glipizide	Glipizide	218	4	4	4	2
Glucagon	Glucagon	1081	463	353	425	102

Glyburide	Glyburide	9401	123	51	118	62
Glycerin	Glycerin	15	270442	117054	254236	50036
Glycopyrrolate	Glycopyrrolate	199	35	4	32	23
Glycopyrronium	Glycopyrronium	15	34	4	31	22
Golimumab	Golimumab	40	12	3	12	5
Gramicidin	Gramicidin	31	26	7	25	3
Granisetron	Granisetron	28	31	5	27	24
Griseofulvin	Griseofulvin	15	26	2	26	9
Guaifenesin	Guaifenesin	3828	5707	2121	5248	1460
Guanfacine	Guanfacine	121	11	0	11	7
Halcinonide	Halcinonide	39	1	0	1	1
Halobetasol	Halobetasol	195	1	0	1	0
Haloperidol	Haloperidol	60	83	17	80	12
HC	HC	2264	25288	14301	23720	4133
HC Ace	HC Ace	82	0	0	0	0
Heme	Heme	159	56644	30018	53389	8556
Heme Iron Polypeptide	Heme Iron Polypeptide	21	45159	25086	42635	6431
Heparin	Heparin	4407	39760	19754	37489	7534
Homatropine Methylbromide	Homatropine Methylbromide	694	2	1	2	2
Hyaluronate	Hyaluronate	20	441	213	375	88
Hydralazine	Hydralazine	275	314	42	309	173
Hydrochlorothiazide	Hydrochlorothiazide	3075	497	118	480	153
Hydrocodone Bitartrate	Hydrocodone	18475	2455	667	2353	1041
Hydrocodone Polistirex	Hydrocodone	669	0	0	0	0
Hydrocortisone Sodium	Hydrocortisone	18	2997	2218	2769	523
Hydrocortisone Valerate	Hydrocortisone	382	0	0	0	0
Hydrocortisone	Hydrocortisone	14641	6710	3615	6269	1516
Hydrocortisone Butyrate	Hydrocortisone Butyrate	167	2997	2218	2769	523
Hydromorphone	Hydromorphone	619	64	5	62	38
Hydroquinone	Hydroquinone	26	2155	745	1934	541
Hydroxychloroquine	Hydroxychloroquine	1877	296	61	284	60
Hydroxyprogesterone Caproate	Hydroxyprogesterone Caproate	5295	242	47	232	175
Hydroxyurea	Hydroxyurea	13	28183	17171	26031	4653
Hydroxyzine	Hydroxyzine	5206	35	2	35	13
Hydroxyzine Pamoate	Hydroxyzine	6442	8	1	8	3
Hyoscyamine	Hyoscyamine	430	22558	11142	21120	3459
Hyoscyamine Sulf	Hyoscyamine Sulf	42	2	0	2	0
Hypochlorous Acid	Hypochlorous Acid	12	10	2	9	1
Hypromellose	Hypromellose	14	23	13	21	6
Ibuprofen	Ibuprofen	10774	30877	14160	28577	4562
Icosapent Ethyl	Icosapent Ethyl	33	1	0	1	0
IF	IF	23	223536	99999	209414	43886
Imipramine	Imipramine	27	116	61	93	19
Imiquimod	Imiquimod	314	21	3	16	8
Immune Globulin	Immune Globulin	57	237984	108017	223173	46088
Indapamide	Indapamide	18	1	0	1	1
Indomethacin	Indomethacin	2479	3477	812	3309	1016
Infliximab	Infliximab	44	388	162	371	97
Insulin Aspart	Insulin Aspart	5109	1928	1355	1783	284
Insulin Aspart Protamine	Insulin Aspart Protamine	105	1928	1355	1783	284
Insulin Degludec	Insulin Degludec	253	7	3	7	4

Insulin Detemir	Insulin Detemir	3987	27	8	25	18
Insulin Glargine	Insulin Glargine	2793	39	19	37	23
Insulin Glulisine	Insulin Glulisine	39	1	0	1	0
Insulin Human Isophane	Insulin Human Isophane	10578	5	0	4	4
Insulin Human Regular	Insulin Human Regular	1788	11253	8083	10566	2093
Insulin Lispro	Insulin Lispro	4845	54	30	49	32
Insulin Lispro Protamine	Insulin Lispro Protamine	80	52	29	47	32
Interferon Beta-1A	Interferon Beta-1A	24	18	0	18	10
Iodide	Iodide	221	214228	91307	201665	42542
Iodine	Iodine	2156	82692	40018	76922	11940
Iodoquinol	Iodoquinol	44	1	0	1	1
Ipratropium Bromide	Ipratropium	2562	13	0	11	11
Irbesartan	Irbesartan	51	6	0	5	2
Iron	Iron	289	273947	118867	257686	50687
Iron Polysaccharide	Iron Polysaccharide	164	18983	11659	17980	1969
Iron Sucrose	Iron Sucrose	42	18982	11659	17979	1969
Isometheptene Mucate	Isometheptene Mucate	219	1581	557	1500	566
Isoniazid	Isoniazid	30	19966	8523	18502	5035
Isopropyl Alcohol	Isopropyl Alcohol	881	8115	1954	7757	1108
Isosorbide Mononitrate	Isosorbide Mononitrate	14	18271	8422	17014	3694
Isotretinoin	Isotretinoin	28	159	26	154	18
Itraconazole	Itraconazole	26	168	32	148	28
Ivacaftor	Ivacaftor	27	29	6	28	3
Ivermectin	Ivermectin	235	217	27	198	83
Ixekizumab	Ixekizumab	15	4	1	3	0
K Cl	K Cl	151	271	77	253	56
K Phos	K Phos	11	25	14	19	5
Ketoconazole	Ketoconazole	3710	73	13	65	20
Ketorolac Tromethamine	Ketorolac Tromethamine	753	85	10	70	68
Ketotifen	Ketotifen	15	18	3	17	8
L-Methylfolate	L-Methylfolate	751	9	6	9	6
Labetalol	Labetalol	17382	310	49	303	150
Lacosamide	Lacosamide	78	19	6	18	4
Lactobacillus Acidophilus	Lactobacillus Acidophilus	14	4902	3252	4536	1038
Lactobacillus c	Lactobacillus c	462	65	30	57	29
Lactobacillus casei	Lactobacillus casei	11	11036	3883	10644	3712
Lactulose	Lactulose	510	110	70	107	50
Lamivudine	Lamivudine	61	664	203	641	396
Lamotrigine	Lamotrigine	2861	285	88	267	104
Lancet	Lancet	41741	32	13	28	6
Lansoprazole	Lansoprazole	1847	19	4	19	9
Latanoprost	Latanoprost	150	3	0	3	0
Lauric Acid	Lauric Acid	2334	101199	42213	94398	19115
Leflunomide	Leflunomide	13	47	11	46	12
Letrozole	Letrozole	5564	414	39	390	315
Leuprolide	Leuprolide	1761	473	129	451	251
Levalbuterol	Levalbuterol	613	4	1	4	4
Levetiracetam	Levetiracetam	1149	205	52	198	55
Levocetirizine Dihydrochloride	Levocetirizine	1622	7	0	5	5
Levodopa	Levodopa	17	210	89	199	25
Levofloxacin	Levofloxacin	1338	105	18	94	14

Levom	Levom	221	6	3	6	1
Levomefolate	Levomefolate	151	70	12	62	47
Levomefolate Ca	Levomefolate Ca	285	1	1	1	1
Levomilnacipran	Levomilnacipran	31	0	0	0	0
Levonorgestrel	Levonorgestrel	2840	1472	244	1344	691
Levothyroxine	Levothyroxine	50580	9337	6852	8516	903
Lido	Lido	26	991	166	891	491
Lidocaine	Lidocaine	5174	798	105	702	486
Lifitegrast	Lifitegrast	158	0	0	0	0
Linaclotide	Linaclotide	481	0	0	0	0
Linagliptin	Linagliptin	26	0	0	0	0
Linezolid	Linezolid	23	85	15	79	20
Lingonb	Lingonb	239	0	0	0	0
Liothyronine	Liothyronine	1117	9507	6933	8667	905
Lipase	Lipase	100	274500	119682	258129	50447
Liraglutide	Liraglutide	285	13	1	12	6
Lisdexamfetamine Dimesylate	Lisdexamfetamine Dimesylate	2396	18	5	16	14
Lisinopril	Lisinopril	1549	48	13	45	13
Lithium	Lithium	230	248	116	227	19
Loperamide	Loperamide	120	31	4	30	9
Loratadine	Loratadine	548	25	6	22	17
Lorazepam	Lorazepam	2651	84	21	73	29
Lorcaserin	Lorcaserin	33	2	0	2	1
Losartan	Losartan	803	73	22	69	14
Loteprednol Etabonate	Loteprednol	532	0	0	0	0
Lovastatin	Lovastatin	41	10	2	9	2
Lubiprostone	Lubiprostone	83	1	0	1	1
Luliconazole	Luliconazole	36	3	0	1	2
Lurasidone	Lurasidone	438	3	1	3	0
Luteinizing Hormone	Luteinizing Hormone	2017	9486	4108	8821	2766
Magnesium	Magnesium	1359	100736	46339	94054	26064
Magnesium Hydroxide	Magnesium Hydroxide	17	24804	12708	23173	3978
Magnesium Oxide	Magnesium Oxide	106	16105	7494	14906	4440
Malathion	Malathion	26	30	20	30	0
Mebendazole	Mebendazole	32	66	6	65	22
Meclizine	Meclizine	1417	15	1	14	1
Medroxyprogesterone	Medroxyprogesterone	2589	124917	54030	116526	31319
Mefenamic Acid	Mefenamic Acid	31	788	219	729	369
Mefloquine	Mefloquine	186	206	40	194	132
Meloxicam	Meloxicam	1471	9	1	8	3
Memantine	Memantine	38	3	0	2	2
Meperidine	Meperidine	139	635	95	588	440
Mercaptopurine	Mercaptopurine	107	4895	2995	4488	702
Mesalamine	Mesalamine	1451	94	18	90	35
Mestranol	Mestranol	26	271	91	261	87
Metaxalone	Metaxalone	218	1	1	0	0
Metformin	Metformin	22585	894	279	836	549
Methadone	Methadone	36	913	180	895	310
Methenamine	Methenamine	33	22347	11115	20919	3364
Methenamine Hippurate	Methenamine Hippurate	11	12	6	11	2
Methimazole	Methimazole	727	191	87	178	32
Methocarbamol	Methocarbamol	755	1707	445	1660	694
Methotrexate	Methotrexate	77	2734	691	2684	498
Methylcobalamin	Methylcobalamin	661	888	727	795	107

Methyldopa	Methyldopa	3829	468	134	448	172
Methylene Blue	Methylene Blue	30	22381	11124	20951	3361
Methylergonovine	Methylergonovine	119	127	13	123	62
Methylphenidate	Methylphenidate	939	16280	7096	15162	1997
Methylprednisolone	Methylprednisolone	12697	3167	997	3034	1367
Methylprednisolone Sodium	Methylprednisolone	11	661	174	642	104
Metoclopramide	Metoclopramide	22635	1029	308	956	485
Metoprolol	Metoprolol	3432	133	43	129	45
Metronidazole	Metronidazole	49595	512	58	459	224
Mg Hydroxide	Mg Hydroxide	50	9780	3785	8980	2833
Mg Sulf	Mg Sulf	62	129	77	111	30
Miconazole Nitrate	Miconazole	281	766	108	724	204
Midazolam	Midazolam	11	331	66	303	172
Midodrine	Midodrine	70	6	0	5	1
Milnacipran	Milnacipran	20	1	0	1	1
Minocycline	Minocycline	678	132	15	127	32
Mirabegron	Mirabegron	46	3	0	3	1
Mirtazapine	Mirtazapine	441	26	3	24	5
Misoprostol	Misoprostol	752	2490	124	2376	1864
Mn	Mn	188	277229	120205	260775	50905
Modafinil	Modafinil	141	11	2	11	4
Mold, Suppository	Mold, Suppository	24	0	0	0	0
Mometasone Furoate	Mometasone	2830	17	4	10	13
Montelukast	Montelukast	8787	50	10	39	33
Morphine	Morphine	157	277211	120198	260757	50904
Moxifloxacin	Moxifloxacin	788	45	5	42	10
Mupirocin	Mupirocin	6135	20	0	20	4
Mycophenolate Mofetil	Mycophenolate Mofetil	22	157	35	149	23
Na Bicarb	Na Bicarb	97	9742	3751	8948	2817
Na Cl	Na Cl	161	148	43	136	20
Na Lact	Na Lact	23	2	1	2	0
Na Pho	Na Pho	30	1656	480	1588	272
Na Phos	Na Phos	28	1656	480	1588	272
Na Sulf	Na Sulf	58	0	0	0	0
Nabumetone	Nabumetone	128	1	0	1	0
Nadolol	Nadolol	112	14	1	14	4
Naftifine	Naftifine	106	3	0	3	3
Naloxegol	Naloxegol	17	0	0	0	0
Naloxone	Naloxone	601	597	95	572	288
Naltrexone	Naltrexone	233	877	239	844	146
Naproxen	Naproxen	2644	185	74	169	41
Naratriptan	Naratriptan	145	9	0	8	2
Nebivolol	Nebivolol	195	40	12	38	11
Neomycin	Neomycin	1154	63	10	60	21
Neomycin Sulf	Neomycin Sulf	2309	4	2	4	1
Nia	Nia	67	20558	6895	19534	3298
Niacin	Niacin	238	23817	14794	22441	2947
Niacinamide	Niacinamide	224	5546	3579	5145	1070
Nicotine	Nicotine	422	3421	1079	3312	285
Nicotine Polacrilex	Nicotine Polacrilex	69	2431	630	2374	121
Nifedipine	Nifedipine	11918	649	159	606	346
Nitazoxanide	Nitazoxanide	16	12	0	10	8
Nitrofurantoin	Nitrofurantoin	75065	123	5	119	24
Nitrofurantoin Monohydrate	Nitrofurantoin Monohydrate	71330	122	5	118	24

Nitroglycerin	Nitroglycerin	66	270397	117020	254192	50018
Nizatidine	Nizatidine	27	3	0	3	0
Norelgestromin	Norelgestromin	730	8	2	5	7
Norethindrone	Norethindrone	5839	7666	4141	7028	2377
Norethindrone Ace	Norethindrone Ace	3574	140	43	130	64
Norgestimate	Norgestimate	6708	41	11	35	25
Norgestrel	Norgestrel	581	1535	278	1405	743
Nortriptyline	Nortriptyline	435	104	25	98	36
Nystatin	Nystatin	10048	301	91	291	61
Ofloxacin	Ofloxacin	1635	5092	2919	4838	1048
Olanzapine	Olanzapine	191	75	14	68	16
Olive Oil	Olive Oil	46	169	71	142	137
Olmesartan Medoxomil	Olmesartan Medoxomil	93	56	16	52	12
Olopatadine	Olopatadine	1387	3	1	3	2
Omalizumab	Omalizumab	125	108	26	102	48
Omega-3	Omega-3	256	36005	14678	33516	7226
Omega-3 Fatty Acids	Omega-3 Fatty Acids	641	1523	1067	1451	650
Omega-3-Acid Ethyl Esters	Omega-3-Acid Ethyl Esters	150	213	123	203	87
Omeprazole	Omeprazole	11895	1894	491	1838	771
OnabotulinumtoxinA	OnabotulinumtoxinA	134	8	0	8	1
Ondansetron	Ondansetron	95193	185	12	168	123
Orphenadrine	Orphenadrine	101	1706	445	1660	693
Oseltamivir	Oseltamivir	21416	289	33	276	53
Oxaprozin	Oxaprozin	13	1	0	1	1
Oxazepam	Oxazepam	17	36	19	26	11
Oxcarbazepine	Oxcarbazepine	336	81	27	78	29
Oxiconazole Nitrate	Oxiconazole	109	0	0	0	0
Oxybutynin	Oxybutynin	369	15	1	14	4
Oxycodone	Oxycodone	11067	2937	785	2794	1327
Oxymetazoline	Oxymetazoline	42	18446	5115	17165	6944
Oxyquinoline	Oxyquinoline	17	4595	3335	4239	833
Oxytocin	Oxytocin	68	4284	790	4030	2419
Paliperidone	Paliperidone	15	13	4	13	4
Pantoprazole	Pantoprazole	11831	82	14	78	35
Paroxetine	Paroxetine	770	103	28	97	27
PEG	PEG	21	1604	602	1455	399
PEG Electrolyte Lavage Solution	PEG Electrolyte Lavage Solution	143	0	0	0	0
Penciclovir	Penciclovir	128	572	121	546	156
Penicillin G Benzathine	Penicillin G Benzathine	48	125	15	124	30
Penicillin V	Penicillin V	5162	3174	1133	2962	701
Pentosan Polysulfate	Pentosan Polysulfate	83	4	1	4	1
Pentoxifylline	Pentoxifylline	53	73	15	68	34
Permethrin	Permethrin	880	83	36	75	28
Perphenazine	Perphenazine	11	7	2	7	1
Phenazopyridine	Phenazopyridine	3202	4	3	4	1
Phendimetrazine	Phendimetrazine	20	0	0	0	0
Phenobarbital	Phenobarbital	16	929	370	864	240
Phentermine	Phentermine	505	23	1	21	12
Phenyleph	Phenyleph	252	625	150	574	318
Phenytoin	Phenytoin	34	1208	486	1116	185
Phytonadione	Phytonadione	186	714	331	690	143
Pilocarpine	Pilocarpine	22	18	9	16	0
Pimecrolimus	Pimecrolimus	303	6	2	5	6
Pindolol	Pindolol	69	32	11	31	14

Pioglitazone	Pioglitazone	76	98424	41378	93513	15018
Piroxicam	Piroxicam	91	28	1	26	24
Plecanatide	Plecanatide	32	0	0	0	0
Podofilox	Podofilox	35	200	109	186	33
Polyethylene Glycol 3350	Polyethylene Glycol 3350	1517	3	2	3	1
Polymyxin B	Polymyxin B	3661	19	6	18	5
Polymyxin B Sulf	Polymyxin B Sulf	2264	3	0	3	0
Potassium Nitrate	Potassium	349	37	1	37	12
Potassium	Potassium	2159	242246	107828	227376	43835
Potassium Bicarbonate	Potassium Bicarbonate	14	10	9	7	1
Pramipexole Dihydrochloride	Pramipexole	79	6	0	6	0
Pramoxine	Pramoxine	2528	6	0	6	4
Pravastatin	Pravastatin	138	1746	458	1698	711
Prazosin	Prazosin	200	729	442	578	66
Prednisolone	Prednisolone	1119	3139	939	3012	995
Prednisolone Sodium	Prednisolone	98	1581	416	1524	249
Prednisone	Prednisone	16739	1234	332	1201	238
Pregabalin	Pregabalin	258	32	2	28	14
Prenata	Prenata	3770	33021	15044	31821	3742
Prilocaine	Prilocaine	685	75	12	59	55
Prochlorperazine	Prochlorperazine	2666	2459	822	2302	466
Progesterone	Progesterone	66490	275802	119533	259444	50752
Progesterone, Wettable	Progesterone, Wettable	133	0	0	0	0
Progestin	Progestin	179	274736	118964	258480	50531
Proguanil	Proguanil	121	60	14	60	29
Promethazine	Promethazine	50000	863	226	803	407
Propranolol	Propranolol	2135	434	123	411	91
Propylthiouracil	Propylthiouracil	658	8560	3025	8173	1781
Protease	Protease	100	265044	114450	249102	49397
PSE	PSE	1546	5036	1412	4759	856
Pseudoephedrine	Pseudoephedrine	280	23876	10956	22037	4818
Pyr	Pyr	17	5293	2589	4881	1323
Pyridostigmine Bromide	Pyridostigmine	67	46	6	46	1
Pyridoxal Phos	Pyridoxal Phos	279	101	81	94	20
Pyridoxine	Pyridoxine	55353	267882	115986	252474	49273
Quetiapine	Quetiapine	1131	66	21	58	17
Rabeprazole	Rabeprazole	200	74	11	70	33
Raltegravir	Raltegravir	73	382	175	350	221
Ramipril	Ramipril	23	9	2	8	2
Ranitidine	Ranitidine	10513	92	18	91	49
Reduced Diphtheria Toxoid	Reduced Diphtheria Toxoid	22719	68	11	65	28
Rho(D) Immune Globulin	Rho(D) Immune Globulin	2685	496	387	455	61
Rifampin	Rifampin	38	546	84	524	102
Rifaximin	Rifaximin	91	40	17	37	12
Rilpivirine	Rilpivirine	61	24	10	23	16
Risperidone	Risperidone	146	109	28	104	44
Ritonavir	Ritonavir	71	371	174	339	214
Rivaroxaban	Rivaroxaban	107	35	12	31	5
Rizatriptan	Rizatriptan	1308	4	0	3	1
Ropinirole	Ropinirole	127	5	2	4	0

Rosuvastatin	Rosuvastatin	174	28	15	26	7
Salicylic Acid	Salicylic Acid	66	22207	8715	20693	4381
Salmeterol Xinafoate	Salmeterol Xinafoate	2567	17	1	12	17
Sapropterin Dihydrochloride	Sapropterin	21	22	12	20	6
Saxagliptin	Saxagliptin	11	792	230	738	501
Sch	Sch	34	53656	20420	50998	12427
Schi	Schi	181	1359	499	1267	135
Schizochytrium	Schizochytrium	54	10	3	8	8
Scopolamine	Scopolamine	1233	70	9	61	43
Secnidazole	Secnidazole	57	4	0	2	3
Secukinumab	Secukinumab	38	8	0	7	1
Selenium Sulfide	Selenium Sulfide	197	1	0	1	0
Semaglutide	Semaglutide	67	128683	60540	121013	31457
Senna	Senna	17	107	40	103	15
Sennosides	Sennosides	33	9	2	9	5
Sertraline	Sertraline	25700	96	38	90	36
Sesame Oil	Sesame Oil	12	19	5	13	9
Sildenafil	Sildenafil	21	216	31	206	104
Silver Sulfadiazine	Silver Sulfadiazine	686	17	0	17	5
Simethicone	Simethicone	67	70	15	66	48
Simvastatin	Simvastatin	186	223	93	197	59
Sitagliptin	Sitagliptin	229	12	7	11	10
Sodium	Sodium	487	276628	119966	260228	50798
Sodium Bicarbonate	Sodium Bicarbonate	48	15208	7090	13999	3741
Sodium Ferric Gluconate Complex	Sodium Ferric Gluconate Complex	14	2	0	2	0
Sodium Fluoride	Sodium Fluoride	1605	2798	1501	2637	516
Sodium Oxybate	Sodium Oxybate	56	17	11	11	3
Sodium Picosulfate	Sodium Picosulfate	43	2	0	2	1
Solifenacin	Solifenacin	41	5	0	3	3
Somatropin, E-Coli Derived	Somatropin, E-Coli Derived	14	59	17	52	46
Sotalol	Sotalol	57	356	106	350	50
Soybean Oil	Soybean Oil	321	164	107	156	143
Spinosad	Spinosad	34	2	0	2	1
Spironolactone	Spironolactone	965	492	161	470	129
Succinic Acid	Succinic Acid	428	2629	1404	2464	443
Sucralfate	Sucralfate	1860	6	0	6	0
Sulfacetamide	Sulfacetamide	1086	3	0	3	2
Sulfamethoxazole	Sulfamethoxazole	8010	864	101	849	182
Sulfasalazine	Sulfasalazine	196	603	468	542	90
Sulfur	Sulfur	497	140356	62355	131157	27650
Sulindac	Sulindac	37	18	3	18	9
Sumatriptan	Sumatriptan	3561	42	6	39	14
Suvorexant	Suvorexant	34	0	0	0	0
Tacrolimus	Tacrolimus	552	294	109	269	49
Tamoxifen	Tamoxifen	65	175	29	157	52
Tamsulosin	Tamsulosin	1102	12	2	11	5
Tapentadol	Tapentadol	46	4	2	4	4
Tavaborole	Tavaborole	52	0	0	0	0
Tazarotene	Tazarotene	110	2	0	2	1
Telmisartan	Telmisartan	20	43	11	41	10
Temazepam	Temazepam	196	12	5	11	3
Tenofovir	Tenofovir	30	955	331	893	588
Tenofovir Al	Tenofovir Al	35	34	10	32	25

Tenofovir Alafenamide	Tenofovir Alafenamide	76	460	161	417	289
Tenofovir Di	Tenofovir Di	21	178	68	168	126
Tenofovir Disoproxil	Tenofovir Disoproxil	386	941	323	884	579
Terbinafine	Terbinafine	296	23	2	22	10
Terbutaline	Terbutaline	276	305	45	293	183
Terconazole	Terconazole	31729	1	0	1	1
Testosterone Cypionate	Testosterone Cypionate	14	3	2	3	0
Tetanus Toxoid	Tetanus Toxoid	12	65174	33426	60699	10361
Tetracaine	Tetracaine	457	58	8	55	39
Tetracycline	Tetracycline	30	338	27	325	50
Tezacaftor	Tezacaftor	23	13	3	13	0
Thonzonium Brom	Thonzonium Brom	45	0	0	0	0
Thyroid	Thyroid	2362	143274	57934	135305	27654
Timolol	Timolol	170	64	14	61	15
Tinidazole	Tinidazole	669	498	68	461	91
Tiotropium Bromide	Tiotropium	94	4	2	4	2
Tizanidine	Tizanidine	878	2	0	2	0
Tobramycin	Tobramycin	3890	492	242	453	99
Tofacitinib	Tofacitinib	15	16	0	16	3
Tolterodine	Tolterodine	48	4	1	3	2
Topiramate	Topiramate	1603	173	40	162	51
Torsemide	Torsemide	24	115	39	105	24
Tramadol	Tramadol	3699	1113	260	1010	532
Tranexamic Acid	Tranexamic Acid	137	264	50	234	115
Travoprost	Travoprost	22	1	0	1	0
Trazodone	Trazodone	2602	12	3	12	5
Tretinoin	Tretinoin	1176	205702	89820	192609	43270
Triamcinolone Acetonide	Triamcinolone Acetonide	16857	62	5	57	23
Triamterene	Triamterene	271	412	132	397	115
Triazolam	Triazolam	151	6	3	4	1
Trifluridine	Trifluridine	20	53	46	47	2
Trimethobenzamide	Trimethobenzamide	89	20	10	19	1
Trimethoprim	Trimethoprim	10499	954	129	930	200
Ubrogepant	Ubrogepant	14	0	0	0	0
Ulipristal	Ulipristal	29	5820	1993	5508	1002
Umeclidinium	Umeclidinium	20	2	0	0	2
Urea	Urea	84	41477	23595	38443	5965
Urofollitropin	Urofollitropin	36	74	18	66	43
Ursodiol	Ursodiol	3934	2360	1521	2150	298
Ustekinumab	Ustekinumab	119	36	15	34	15
Valacyclovir	Valacyclovir	35201	60	17	57	24
Valsartan	Valsartan	144	54	16	51	13
Vancomycin	Vancomycin	191	617	177	586	137
Varenicline	Varenicline	291	10	0	10	3
Vedolizumab	Vedolizumab	13	23	10	23	16
Venlafaxine	Venlafaxine	2645	81	28	77	25
Verapamil	Verapamil	289	159	31	152	30
Vilanterol Trifenatate	Vilanterol	654	0	0	0	0
Vilazodone	Vilazodone	255	5	1	5	1
Vitamin	Vitamin	2899	266265	113870	250402	50388
Vitamin B	Vitamin B	1703	80452	33336	75843	18498
Vitamin B12	Vitamin B12	90903	101780	44825	95037	16457
Vitamin B2	Vitamin B2	16	26326	16547	24722	3384
Vitamin B3	Vitamin B3	5318	832	388	787	134

Vitamin B6	Vitamin B6	5905	26859	16819	25211	3495
Vitamin C	Vitamin C	109477	60911	30804	56719	10769
Vitamin D	Vitamin D	64	138031	57019	129948	25057
Vitamin E	Vitamin E	87	21115	10106	19961	3695
Vortioxetine Hydrobromide	Vortioxetine	271	2	0	2	0
Warfarin	Warfarin	93	476	80	463	62
Water, Sterile	Water, Sterile	38	3973	1906	3564	801
Witepsol H 15	Witepsol H 15	40	0	0	0	0
Zafirlukast	Zafirlukast	25	10	1	8	9
Zaleplon	Zaleplon	85	1	0	1	0
Zanamivir	Zanamivir	13	112	20	105	25
Zidovudine	Zidovudine	26	5261	1348	5025	1871
Zinc	Zinc	565	2238	1795	2082	418
Ziprasidone	Ziprasidone	89	14	3	13	6
Zolmitriptan	Zolmitriptan	183	0	0	0	0
Zolpidem	Zolpidem	5487	1122	510	1096	37
Zonisamide	Zonisamide	165	28	14	26	12

Postpartum Drug Publication Frequency

Original Drug Name	Cleaned Drug Name	postpartum frequency	All publication	PK publication	PE publication	CT publication
Abacavir	Abacavir	29	108	41	101	31
Abatacept	Abatacept	28	3	1	3	1
Acamprosate	Acamprosate	17	2	0	2	0
Acarbose	Acarbose	19	0	0	0	0
Acebutolol	Acebutolol	61	7	5	6	1
Acetaminophen	Acetaminophen	249682	5841	1873	5427	1597
Acetazolamide	Acetazolamide	206	10	1	10	3
Acetic Acid	Acetic Acid	134	5627	2852	5222	1266
Acetic Acid Glacial	Acetic Acid Glacial	14	93	60	77	16
Acyclovir	Acyclovir	4319	46	14	45	8
Adalimumab	Adalimumab	870	18	7	17	8
Adapalene	Adapalene	997	112	62	105	25
Al Hydroxide	Al Hydroxide	63	3	0	3	1
Albendazole	Albendazole	55	33	23	25	19
Albuterol	Albuterol	17628	40	1	36	18
Alcaftadine	Alcaftadine	27	0	0	0	0
Alclometasone Dipropionate	Alclometasone	174	0	0	0	0
Alendronate	Alendronate	24	9	1	8	1
Algal Oil	Algal Oil	108	4	2	3	4
Allopurinol	Allopurinol	62	9	4	9	4
Almotriptan	Almotriptan	22	1	1	1	1
Aloe	Aloe	21	6916	3349	6382	1760
Alprazolam	Alprazolam	8646	8	4	6	0
Aluminum	Aluminum	340	43928	20573	40865	9486
Amantadine	Amantadine	29	6	2	4	0
Amiloride	Amiloride	20	46	25	35	6
Amino Acids	Amino Acids	50	43956	20582	40890	9488
Aminocaproic Acid	Aminocaproic Acid	12	11	4	9	0
Amiodarone	Amiodarone	14	31	8	30	3
Amitriptyline	Amitriptyline	1212	8	4	7	2
Amlodipine Besylate	Amlodipine Besylate	3471	17	5	16	5
Ammonium	Ammonium	386	3482	2190	3188	684
Amoxicillin	Amoxicillin	75125	4097	1746	3810	1290
Amphetamine	Amphetamine	48	32056	13517	29759	8178

Ampicillin	Ampicillin	1138	29747	12378	27612	7230
Amylase	Amylase	117	70	49	62	5
Anastrozole	Anastrozole	28	1	0	1	0
Antibacterial	Antibacterial	124	2087	639	1987	445
APAP	APAP	266	5061	1763	4708	1270
Apixaban	Apixaban	389	2	1	2	0
Apraclonidine	Apraclonidine	12	0	0	0	0
Apremilast	Apremilast	63	0	0	0	0
Aprepitant	Aprepitant	33	15	7	13	0
Aripiprazole	Aripiprazole	1347	13	1	13	2
Armodafinil	Armodafinil	81	3	2	3	1
Ascorbic Acid	Ascorbic Acid	128	5846	3758	5395	967
Ascorbyl	Ascorbyl	457	226	158	208	52
Asenapine	Asenapine	18	0	0	0	0
Aspirin	Aspirin	1168	3667	1769	3348	973
Atazanavir	Atazanavir	18	49	23	48	7
Atenolol	Atenolol	692	12	9	10	3
Atomoxetine	Atomoxetine	231	2	0	2	1
Atorvastatin	Atorvastatin	942	142	33	133	51
Atovaquone	Atovaquone	94	3	0	3	2
Atropine	Atropine	530	26	8	22	15
Atropine Sulf	Atropine Sulf	16	0	0	0	0
Azathioprine	Azathioprine	435	154	87	144	16
Azelaic Acid	Azelaic Acid	2295	0	0	0	0
Azelastine	Azelastine	2457	0	0	0	0
Azithromycin	Azithromycin	32201	44	6	41	27
Aztreonam	Aztreonam	15	3	1	3	2
Bacitracin	Bacitracin	247	4	0	4	3
Baclofen	Baclofen	921	16	3	16	4
Baloxavir Marboxil	Baloxavir	82	0	0	0	0
Balsalazide Disodium	Balsalazide	87	0	0	0	0
Barrier Skin Protectant	Barrier Skin Protectant	61	4	2	4	2
Beclomethasone Dipropionate	Beclomethasone	778	2	0	2	2
Belimumab	Belimumab	12	3	3	2	1
Benazepril	Benazepril	162	2	1	2	1
Benzocaine	Benzocaine	33	347	179	326	83
Benzonatate	Benzonatate	7937	0	0	0	0
Benzoyl Peroxide	Benzoyl Peroxide	1904	18	5	18	1
Benztropine Mesylate	Benztropine	85	0	0	0	0
Bepotastine Besilate	Bepotastine	51	0	0	0	0
Besifloxacin	Besifloxacin	75	0	0	0	0
Betaine	Betaine	28	188	125	171	66
Betamethasone Dipropionate	Betamethasone	6213	0	0	0	0
Betamethasone Valerate	Betamethasone	3668	1	1	1	0
Betamethasone Ace	Betamethasone Ace	11	1	1	1	1
Betamethasone Na Phos	Betamethasone Na Phos	11	0	0	0	0
Bethanechol	Bethanechol	49	0	0	0	0
Bi Subcitrate K	Bi Subcitrate K	15	0	0	0	0
Bictegravir	Bictegravir	38	2	2	2	2
Bif	Bif	14	269	79	244	156
Bifidobacterium breve	Bifidobacterium breve	14	26	3	21	22
Bifidobacterium infantis	Bifidobacterium infantis	16	289	76	266	33

Bifidobacterium lactis	Bifidobacterium lactis	15	42	11	41	42
Bimatoprost	Bimatoprost	55	0	0	0	0
Bioflavonoid	Bioflavonoid	465	225	157	207	52
Biotin	Biotin	12630	393	293	354	100
Bisacodyl	Bisacodyl	127	1391	618	1307	343
Bisoprolol	Bisoprolol	154	7	3	6	1
Boron	Boron	37	25	20	22	2
Brexpiprazole	Brexpiprazole	115	0	0	0	0
Brimonidine	Brimonidine	130	0	0	0	0
Brinzolamide	Brinzolamide	14	0	0	0	0
Brivaracetam	Brivaracetam	13	2	2	2	1
Bromfenac	Bromfenac	35	0	0	0	0
Bromocriptine Mesylate	Bromocriptine	172	297	120	287	80
Brompheniramine Mal	Brompheniramine Mal	2976	0	0	0	0
Budesonide	Budesonide	2870	17	1	16	10
Bumetanide	Bumetanide	63	6	2	4	2
Buprenorphine	Buprenorphine	1103	111	21	107	49
Bupropion	Bupropion	9918	15	7	13	5
Buspirone	Buspirone	5212	1	0	1	0
Butalbital	Butalbital	5695	4516	1553	4232	1038
Butoconazole Nitrate	Butoconazole	79	0	0	0	0
Butorphanol	Butorphanol	38	9	1	8	7
C1 Esterase Inhibitor	C1 Esterase Inhibitor	11	23	11	21	1
Ca	Ca	30916	43726	20464	40684	9449
Ca As	Ca As	86	17	5	17	6
Ca Ascorbate	Ca Ascorbate	1024	0	0	0	0
Ca Pantothenate	Ca Pantothenate	3517	0	0	0	0
Cabergoline	Cabergoline	332	38	12	37	15
Caff	Caff	221	514	171	487	202
Caffeine	Caffeine	5432	7210	2923	6738	1495
Calcipotriene	Calcipotriene	448	0	0	0	0
Calcitriol	Calcitriol	180	91	67	86	13
Calcium	Calcium	724	43862	20520	40807	9473
Canagliflozin	Canagliflozin	37	0	0	0	0
Candesartan Cilexetil	Candesartan Cilexetil	19	1	1	1	0
Capecitabine	Capecitabine	18	0	0	0	0
Captopril	Captopril	46	41897	19421	39030	9025
Carbamazepine	Carbamazepine	166	62	38	55	10
Carbidopa	Carbidopa	17	4	3	4	0
Carbinoxamine	Carbinoxamine	41	0	0	0	0
Cariprazine	Cariprazine	107	0	0	0	0
Carisoprodol	Carisoprodol	265	951	467	855	170
Carvedilol	Carvedilol	607	11	7	11	2
Cefaclor	Cefaclor	42	248	84	222	56
Cefadroxil	Cefadroxil	949	1	1	1	0
Cefdinir	Cefdinir	7139	4	2	4	0
Cefixime	Cefixime	172	4	2	3	2
Cefpodoxime Proxetil	Cefpodoxime Proxetil	294	11	4	11	7
Cefprozil	Cefprozil	357	1	1	1	1
Ceftriaxone	Ceftriaxone	77	40	9	38	16
Cefuroxime Axetil	Cefuroxime Axetil	3060	18	4	17	6
Celecoxib	Celecoxib	623	8	3	8	5
Cephalexin	Cephalexin	49338	9	2	9	2
Certolizumab Pegol	Certolizumab Pegol	289	8	4	8	3
Cetirizine	Cetirizine	625	6	4	5	1
Cetrorelix	Cetrorelix	70	0	0	0	0

Chlophedianol	Chlophedianol	18	0	0	0	0
Chlordiazepoxide	Chlordiazepoxide	59	113	77	98	27
Chlorhexidine	Chlorhexidine	4696	55	3	48	38
Chloroquine	Chloroquine	11	70	16	65	23
Chlorpheniramine Polistirex	Chlorpheniramine	463	0	0	0	0
Chlorpheniramine Mal	Chlorpheniramine Mal	22	1	0	1	1
Chlorpromazine	Chlorpromazine	15	32	15	30	1
Chlorthalidone	Chlorthalidone	343	12	9	10	3
Chlorzoxazone	Chlorzoxazone	95	208	54	198	110
Cholecalc	Cholecalc	2536	136	120	121	71
Cholecalcif	Cholecalcif	401	136	120	121	71
Cholecalcife	Cholecalcife	442	136	120	121	71
Cholecalcifer	Cholecalcifer	44	136	120	121	71
Cholecalciferol	Cholecalciferol	28950	19014	8066	17668	4056
Cholestyramine	Cholestyramine	231	6	3	5	1
Choline	Choline	624	25269	11667	23420	6134
Chorionic Gonadotropin	Chorionic Gonadotropin	135	319	221	293	23
Chorionic Gonadotropin Alfa	Chorionic Gonadotropin Alfa	80	51	35	44	1
Ciclesonide	Ciclesonide	49	0	0	0	0
Ciclopirox	Ciclopirox	909	0	0	0	0
Ciclopirox Olamine	Ciclopirox	215	0	0	0	0
Cimetidine	Cimetidine	109	14	8	12	6
Ciprofloxacin	Ciprofloxacin	10606	84	43	78	9
Citalopram Hydrobromide	Citalopram	8982	40	25	34	13
Citric Acid	Citric Acid	296	1271	598	1159	322
Clarithromycin	Clarithromycin	1121	5089	2184	4797	752
Clavulanate	Clavulanate	34633	98	22	94	43
Clidinium Bromide	Clidinium	28	1	1	0	0
Clindamycin	Clindamycin	21813	74	7	71	42
Clindamycin Palmitate	Clindamycin	62	74	7	71	42
Clioquinol	Clioquinol	11	1	0	1	0
Clobazam	Clobazam	14	5811	2078	5471	1346
Clobetasol	Clobetasol	5063	1	0	0	1
Clocortolone Pivalate	Clocortolone	67	0	0	0	0
Clomiphene	Clomiphene	179	42	12	41	4
Clomipramine	Clomipramine	48	6	3	6	0
Clonazepam	Clonazepam	3999	13	3	11	1
Clonidine	Clonidine	622	25	10	24	11
Clopidogrel Hydrogen	Clopidogrel Hydrogen	109	2	0	2	0
Clorazepate Dipotassium	Clorazepate	37	0	0	0	0
Clotrimazole	Clotrimazole	5197	3	0	2	1
Cobamamide	Cobamamide	33	189	150	178	32
Cobicistat	Cobicistat	56	9	7	7	6
Codeine Polistirex	Codeine	17	0	0	0	0
Codeine	Codeine	24672	513	172	487	207
Codeine Phos	Codeine Phos	305	2	0	2	2
Coenzyme Q10	Coenzyme Q10	21	8	8	8	2
Colchicine	Colchicine	102	8	1	8	2
Colesevelam	Colesevelam	91	0	0	0	0
Colestipol	Colestipol	110	0	0	0	0
Colistin Sulf	Colistin Sulf	38	0	0	0	0
Collagenase	Collagenase	21	5415	2250	4946	1220

Colloidal Sulfur	Colloidal Sulfur	21	3	0	3	1
Conjugated Estrogens	Conjugated Estrogens	1814	208	130	190	22
Copper	Copper	1963	26790	12181	24897	5400
Cr	Cr	117	33306	16514	30877	6988
Cranberry	Cranberry	17	822	167	797	52
Crisaborole	Crisaborole	271	0	0	0	0
Cromolyn	Cromolyn	102	22	7	20	15
Cu	Cu	24694	26202	11769	24342	5253
Cu Oxide	Cu Oxide	13	0	0	0	0
Cu Sulf	Cu Sulf	39	1	1	1	1
Cyanocobalamin	Cyanocobalamin	1395	5866	3908	5396	989
Cyclobenzaprine	Cyclobenzaprine	9281	1	1	1	0
Cyclopentolate	Cyclopentolate	54	4	0	4	1
Cyclosporine	Cyclosporine	361	215	102	200	27
Cyproheptadine	Cyproheptadine	188	6	4	6	1
Dabigatran Etexilate Mesylate	Dabigatran Etexilate	14	1	1	0	0
Dapagliflozin Propanediol	Dapagliflozin Propanediol	78	1	0	1	1
Dapsone	Dapsone	620	3255	901	3132	609
Darunavir Ethanolate	Darunavir Ethanolate	21	18	14	16	13
Dasatinib	Dasatinib	12	1	0	1	0
Desipramine	Desipramine	14	12627	4613	11854	5162
Desloratadine	Desloratadine	156	2	2	1	1
Desmopressin	Desmopressin	62	57	20	57	3
Desogestrel	Desogestrel	5119	66	8	64	37
Desonide	Desonide	946	16	1	15	10
Desoximetasone	Desoximetasone	407	0	0	0	0
Desvenlafaxine	Desvenlafaxine	802	9	8	9	6
Dexamethasone Sodium	Dexamethasone	75	2	1	2	2
Dexamethasone	Dexamethasone	5911	41849	19381	38984	9008
Dexchlorpheniramine Mal	Dexchlorpheniramine Mal	20	0	0	0	0
Dexlansoprazole	Dexlansoprazole	339	0	0	0	0
Dexmethylphenidate	Dexmethylphenidate	118	2	1	1	1
Dextroamphetamine	Dextroamphetamine	114	16	5	15	2
Dextromethorphan Hydrobromide	Dextromethorphan	17	275	97	262	121
Diaphragm	Diaphragm	165	103	22	97	12
Diazepam	Diazepam	2479	7791	4048	7047	1211
Dichloralphenazone	Dichloralphenazone	38	208	54	198	110
Diclofenac	Diclofenac	3900	237	57	223	123
Diclofenac Epolamine	Diclofenac Epolamine	43	1	1	1	0
Dicloxacillin	Dicloxacillin	27324	4	1	4	0
Dicyclomine	Dicyclomine	2062	5	1	5	1
Dienogest	Dienogest	107	1	0	1	0
Diethylpropion	Diethylpropion	18	291	147	265	82
Diflorasone Diacetate	Diflorasone	31	0	0	0	0
Diflunisal	Diflunisal	35	0	0	0	0
Difluprednate	Difluprednate	131	0	0	0	0
Digoxin	Digoxin	65	95	32	93	10
Dihydroergotamine Mesylate	Dihydroergotamine	33	0	0	0	0
Diltiazem	Diltiazem	288	4	0	3	2
Dimethyl	Dimethyl	141	43687	20441	40638	9472
Diphenhydram	Diphenhydram	63	7	1	5	3

diphenhydrAMINE	diphenhydrAMINE	65	767	242	719	223
Diphenoxylate	Diphenoxylate	503	1	0	1	0
Disodiu	Disodiu	18	4	0	4	2
Disulfiram	Disulfiram	26	2	0	2	0
Divalproex	Divalproex	263	107	60	99	20
DM Hydrobrom	DM Hydrobrom	4762	0	0	0	0
Docosahexa	Docosahexa	1341	508	427	478	223
Docosahexaeno	Docosahexaeno	388	869	630	812	327
Docosahexaenoic A	Docosahexaenoic A	8027	496	416	468	222
Docosahexaenoic Aci	Docosahexaenoic Aci	28	495	415	467	222
Docosahexaenoic Acid	Docosahexaenoic Acid	11048	865	626	808	327
Docosapentaenoic Acid	Docosapentaenoic Acid	85	33	32	32	7
Docusate	Docusate	5962	11895	5093	11164	5237
Dolutegravir	Dolutegravir	61	20	9	20	12
Dornase Alfa	Dornase Alfa	27	1	0	1	1
Dorzolamide	Dorzolamide	85	1	0	1	0
Doxazosin Mesylate	Doxazosin	22	2	1	2	0
Doxepin	Doxepin	271	5	3	4	0
Doxycycline	Doxycycline	2151	13	2	10	5
Doxycycline Hyclate	Doxycycline Hyclate	7619	13	2	10	5
Doxylamine	Doxylamine	689	8	3	8	1
Dronabinol	Dronabinol	17	687	127	639	91
Drospirenone	Drospirenone	6275	7	2	7	5
DSS	DSS	3752	47	13	46	10
Dulaglutide	Dulaglutide	175	0	0	0	0
Duloxetine	Duloxetine	2595	8	6	7	3
Dupilumab	Dupilumab	70	2	0	2	0
Econazole Nitrate	Econazole	385	0	0	0	0
Efinaconazole	Efinaconazole	209	0	0	0	0
Eico	Eico	56	200	176	191	70
Eicosap	Eicosap	596	161	144	152	66
Eicosapentaenoic Acid	Eicosapentaenoic Acid	3722	5769	2530	5280	1407
Elagolix	Elagolix	28	0	0	0	0
Eletriptan Hydrobromide	Eletriptan	490	1	1	1	1
Elexacaftor	Elexacaftor	21	6	2	6	0
Eluxadoline	Eluxadoline	23	0	0	0	0
Elvitegravir	Elvitegravir	46	9	7	7	6
Empagliflozin	Empagliflozin	102	0	0	0	0
Emtricitabine	Emtricitabine	275	134	55	130	81
Enalapril	Enalapril	531	10	4	10	4
Enoxaparin	Enoxaparin	7710	60	15	60	21
Epinastine	Epinastine	59	1	1	1	0
Epinephrine	Epinephrine	2080	271	153	254	58
Erenumab-aooe	Erenumab-aooe	89	3	0	3	1
Ergocalciferol	Ergocalciferol	4638	969	739	877	251
Erythromycin	Erythromycin	4641	5716	2465	5352	881
Erythromycin Ethylsuccinate	Erythromycin Ethylsuccinate	40	628	280	553	112
Escitalopram Oxalate	Escitalopram Oxalate	18137	14	9	13	5
Esomeprazole	Esomeprazole	1203	13	2	11	10
Estradiol Valerate	Estradiol	132	571	428	534	141
Estradiol	Estradiol	6329	43910	20564	40853	9485
Estradiol V	Estradiol V	107	10	6	10	5
Estrogen	Estrogen	623	43919	20568	40859	9485
Eszopiclone	Eszopiclone	262	0	0	0	0

Etanercept	Etanercept	255	9	1	9	1
Ethinyl	Ethinyl	118	2723	1188	2556	820
Ethinyl Estrad	Ethinyl Estrad	256	84	34	83	49
Ethinyl Estradiol	Ethinyl Estradiol	98959	124	51	122	68
Ethynodiol Diacetate	Ethynodiol	400	10	4	10	3
Etodolac	Etodolac	308	0	0	0	0
Etonogestrel	Etonogestrel	10786	48	6	48	28
Exenatide	Exenatide	26	0	0	0	0
Ezetimibe	Ezetimibe	47	5	4	5	1
Famciclovir	Famciclovir	213	1	1	0	0
Famotidine	Famotidine	3287	146	40	140	94
Fe Pentacarbonyl	Fe	40	0	0	0	0
Fe	Fe	20132	43239	20283	40217	9419
Fe Polysa	Fe Polysa	28	0	0	0	0
Fe Polysaccharide	Fe Polysaccharide	158	4221	2722	3908	633
Felodipine	Felodipine	11	0	0	0	0
Female Condom	Female Condom	113	3	0	3	0
Fenofibrate	Fenofibrate	142	4	3	4	0
Fenoprofen	Fenoprofen	36	2	0	2	2
Fentanyl	Fentanyl	40	231	33	214	154
Ferrous	Ferrous	3045	5313	3514	4896	919
Ferrous Asparto G	Ferrous Asparto G	985	0	0	0	0
Ferrous Bisglycin	Ferrous Bisglycin	191	1	1	1	1
Ferrous Fu	Ferrous Fu	89	12	8	11	12
Ferrous Fum	Ferrous Fum	20808	12	8	11	12
Fexofenadine	Fexofenadine	96	2	1	2	1
Fidaxomicin	Fidaxomicin	38	0	0	0	0
Filgrastim-sndz	Filgrastim-sndz	12	0	0	0	0
Fingolimod	Fingolimod	50	14	0	14	3
Flavoxate	Flavoxate	18	0	0	0	0
Flecainide	Flecainide	67	22	3	22	5
Flibanserin	Flibanserin	12	0	0	0	0
Fluconazole	Fluconazole	40882	33	10	31	10
Fludrocortisone	Fludrocortisone	113	5	1	5	2
Flunisolide	Flunisolide	35	0	0	0	0
Fluocinolone Acetonide	Fluocinolone Acetonide	1021	0	0	0	0
Fluocinonide	Fluocinonide	1902	0	0	0	0
Fluorometholone	Fluorometholone	260	0	0	0	0
Fluorouracil	Fluorouracil	93	60	24	56	13
Fluoxetine	Fluoxetine	11005	84	42	72	23
Flurandrenolide	Flurandrenolide	114	0	0	0	0
Flurbiprofen	Flurbiprofen	25	7	3	5	6
Fluticasone	Fluticasone	17142	4	0	4	3
Fluticasone Furoate	Fluticasone	673	0	0	0	0
Fluvoxamine	Fluvoxamine	200	14	10	12	3
Fo	Fo	314	40720	18921	37815	9030
Fol	Fol	17	13725	5927	12781	3482
Folate Combinat	Folate Combinat	623	1	0	0	1
Foli	Foli	105	559	272	540	189
Folic	Folic	1044	3176	1764	2950	921
Folic A	Folic A	2353	1758	1084	1639	450
Folic Ac	Folic Ac	3804	527	254	513	187
Folic Acid	Folic Acid	25006	3175	1764	2949	921
Follicle Stimulating Hormone	Follicle Stimulating Hormone	129	414	310	379	68
Follitropin Alfa	Follitropin Alfa	110	24	14	20	1

Follitropin Beta	Follitropin Beta	51	2	2	2	0
Fondaparinux	Fondaparinux	28	7	0	7	1
Formoterol	Formoterol	1625	132	76	127	46
Forskohlii	Forskohlii	18	0	0	0	0
Fosfomycin Tromethamine	Fosfomycin Tromethamine	70	3	2	3	1
Fremanezumab-vfrm	Fremanezumab-vfrm	39	2	0	2	1
Frovatriptan	Frovatriptan	50	0	0	0	0
Furosemide	Furosemide	5734	83	29	80	32
Gabapentin	Gabapentin	3713	17	5	16	8
Galcanezumab-gnlm	Galcanezumab-gnlm	77	2	0	2	1
Ganciclovir	Ganciclovir	33	53	22	50	4
Ganirelix	Ganirelix	59	0	0	0	0
Gatifloxacin	Gatifloxacin	96	0	0	0	0
Gemfibrozil	Gemfibrozil	28	6	3	6	0
Gentamicin	Gentamicin	1147	167	76	160	78
GG	GG	16	16886	7946	15571	3488
Ginger Ext	Ginger Ext	19	0	0	0	0
Glatiramer	Glatiramer	221	15	1	15	7
Glimepiride	Glimepiride	95	0	0	0	0
Glipizide	Glipizide	130	2	2	2	1
Glucagon	Glucagon	268	120	101	112	19
Glucose Meter	Glucose Meter	437	2	2	0	0
Glyburide	Glyburide	604	6	5	6	2
Glycopyrrolate	Glycopyrrolate	207	0	0	0	0
Glycopyrronium	Glycopyrronium	46	0	0	0	0
Golimumab	Golimumab	40	2	2	2	1
Gramicidin	Gramicidin	34	6	2	5	2
Granisetron	Granisetron	11	8	0	6	8
Griseofulvin	Griseofulvin	15	0	0	0	0
Guaifenesin	Guaifenesin	2222	1067	486	972	324
Guanfacine	Guanfacine	89	0	0	0	0
Guselkumab	Guselkumab	23	0	0	0	0
Halcinonide	Halcinonide	48	0	0	0	0
Halobetasol	Halobetasol	294	0	0	0	0
Haloperidol	Haloperidol	59	18	5	16	1
HC	HC	1868	3530	1984	3249	662
HC Ace	HC Ace	65	0	0	0	0
HCTZ	HCTZ	12	67	19	65	22
Heme	Heme	43	10513	5747	9801	1939
Heparin	Heparin	236	6527	3472	6103	1330
Homatropine Methylbromide	Homatropine Methylbromide	552	2	1	2	1
Hyaluronate	Hyaluronate	19	47	31	35	9
Hydralazine	Hydralazine	1010	49	12	47	17
Hydrochlorothiazide	Hydrochlorothiazide	7438	68	20	66	22
Hydrocodone Bitartrate	Hydrocodone	100088	345	87	325	160
Hydrocodone Polistirex	Hydrocodone	446	0	0	0	0
Hydrocortisone Sodium	Hydrocortisone	15	739	544	680	118
Hydrocortisone Valerate	Hydrocortisone	332	0	0	0	0
Hydrocortisone	Hydrocortisone	17269	1317	781	1224	294
Hydrocortisone Butyrate	Hydrocortisone Butyrate	162	739	544	680	118
Hydromorphone	Hydromorphone	5741	21	3	18	13
Hydroquinone	Hydroquinone	58	3790	1244	3341	878

Hydroxychloroquine	Hydroxychloroquine	1783	34	9	30	13
Hydroxyprogesterone Caproate	Hydroxyprogesterone Caproate	97	16	0	16	10
Hydroxyurea	Hydroxyurea	21	4595	3012	4172	891
Hydroxyzine	Hydroxyzine	4372	5	1	4	1
Hydroxyzine Pamoate	Hydroxyzine	2914	0	0	0	0
Hyoscyamine	Hyoscyamine	729	3225	1904	2940	555
Hyoscyamine Sulf	Hyoscyamine Sulf	60	0	0	0	0
Hypochlorous Acid	Hypochlorous Acid	15	3	0	3	1
Ibuprofen	Ibuprofen	324058	5320	2660	4842	1045
Icosapent Ethyl	Icosapent Ethyl	41	0	0	0	0
IF	IF	23	35599	17052	32954	8199
Imipramine	Imipramine	40	41	27	35	9
Imiquimod	Imiquimod	451	5	2	5	2
Immune Globulin	Immune Globulin	34	38141	18602	35378	8678
Indapamide	Indapamide	23	0	0	0	0
Indomethacin	Indomethacin	349	1093	295	1034	296
Infliximab	Infliximab	37	39	24	37	10
Infusion Pump, Insulin	Infusion Pump, Insulin	45	15	8	13	10
Infusion Pump, Parenteral	Infusion Pump, Parenteral	28	0	0	0	0
Ingenol Mebutate	Ingenol Mebutate	15	0	0	0	0
Insulin Aspart	Insulin Aspart	1797	222	158	195	45
Insulin Aspart Protamine	Insulin Aspart Protamine	17	222	158	195	45
Insulin Degludec	Insulin Degludec	233	2	0	2	0
Insulin Detemir	Insulin Detemir	688	2	0	2	1
Insulin Glargine	Insulin Glargine	893	4	3	4	1
Insulin Glulisine	Insulin Glulisine	23	0	0	0	0
Insulin Human Isophane	Insulin Human Isophane	712	0	0	0	0
Insulin Human Regular	Insulin Human Regular	207	1731	1334	1620	263
Insulin Lispro	Insulin Lispro	1490	1	1	1	1
Interferon Beta-1A	Interferon Beta-1A	56	7	2	7	3
Iodide	Iodide	103	32847	14644	30741	7687
Iodine	Iodine	941	14197	7344	13144	2641
Iodoquinol	Iodoquinol	30	0	0	0	0
Ipratropium Bromide	Ipratropium	1413	7	0	7	6
Irbesartan	Irbesartan	74	0	0	0	0
Iron	Iron	245	43568	20422	40525	9447
Iron Polysaccharide	Iron Polysaccharide	148	4221	2722	3908	633
Isometheptene Mucate	Isometheptene Mucate	49	351	135	335	149
Isoniazid	Isoniazid	54	2743	1251	2580	769
Isopropyl Alcohol	Isopropyl Alcohol	86	1128	306	1081	153
Isosorbide Dinitrate	Isosorbide Dinitrate	26	65	20	64	5
Isosorbide Mononitrate	Isosorbide Mononitrate	20	2917	1448	2699	605
Isotretinoin	Isotretinoin	157	3	1	3	1
Itraconazole	Itraconazole	25	10	0	9	1
Ivacaftor	Ivacaftor	29	7	3	7	0
Ivermectin	Ivermectin	504	22	6	17	13
Ixekizumab	Ixekizumab	23	1	1	0	0
K Cl	K Cl	372	60	26	53	15
Ketoconazole	Ketoconazole	4545	6	2	5	2
Ketoprofen	Ketoprofen	29	12	1	10	7
Ketorolac Tromethamine	Ketorolac Tromethamine	4260	28	7	23	22
L-Methylfolate	L-Methylfolate	181	6	6	6	4

Labetalol	Labetalol	31029	54	13	51	21
Lacosamide	Lacosamide	90	7	6	6	1
Lactic Acid	Lactic Acid	88	191	123	176	30
Lactobacillus Acidophilus	Lactobacillus Acidophilus	19	1174	816	1067	269
Lactobacillus c	Lactobacillus c	161	15	6	13	8
Lactobacillus casei	Lactobacillus casei	11	1475	573	1410	577
Lactulose	Lactulose	380	50	34	49	26
Lamivudine	Lamivudine	46	212	70	209	162
Lamotrigine	Lamotrigine	2988	55	32	52	20
Lancet	Lancet	2597	7	4	6	2
Lanolin	Lanolin	15	15	0	6	14
Lansoprazole	Lansoprazole	799	2	0	2	0
Latanoprost	Latanoprost	140	0	0	0	0
Lauric Acid	Lauric Acid	802	16649	7540	15375	3541
Ledipasvir	Ledipasvir	22	2	1	2	1
Leflunomide	Leflunomide	32	10	3	10	1
Letrozole	Letrozole	262	5	2	4	1
Leucovorin	Leucovorin	16	13	5	13	2
Leuprolide	Leuprolide	175	4	0	4	2
Levalbuterol	Levalbuterol	310	0	0	0	0
Levetiracetam	Levetiracetam	1212	36	14	35	11
Levocarnitine	Levocarnitine	27	108	98	100	14
Levocetirizine Dihydrochloride	Levocetirizine	1405	1	1	1	1
Levodopa	Levodopa	17	68	30	67	6
Levofloxacin	Levofloxacin	3188	4	1	3	1
Levom	Levom	103	2	0	2	0
Levomefolate	Levomefolate	66	14	9	14	10
Levomefolate Ca	Levomefolate Ca	647	0	0	0	0
Levomilnacipran	Levomilnacipran	25	0	0	0	0
Levonorgestrel	Levonorgestrel	20048	179	44	173	97
Levothyroxine	Levothyroxine	41354	1628	1261	1485	184
Lido	Lido	18	119	14	105	66
Lidocaine	Lidocaine	7208	112	9	95	73
Lifitegrast	Lifitegrast	182	0	0	0	0
Linaclotide	Linaclotide	479	0	0	0	0
Linagliptin	Linagliptin	12	0	0	0	0
Linezolid	Linezolid	102	5	3	5	2
Lingonb	Lingonb	19	0	0	0	0
Liothyronine	Liothyronine	987	1674	1293	1526	210
Lipase	Lipase	117	43535	20476	40501	9397
Liraglutide	Liraglutide	399	1	0	1	1
Lisdexamfetamine Dimesylate	Lisdexamfetamine Dimesylate	2370	1	0	1	1
Lisinopril	Lisinopril	3439	8	2	8	3
Lithium	Lithium	307	70	32	66	2
Loperamide	Loperamide	143	3	0	3	1
Loratadine	Loratadine	362	4	2	3	1
Lorazepam	Lorazepam	5709	20	2	19	3
Lorcaserin	Lorcaserin	29	0	0	0	0
Losartan	Losartan	1464	10	3	10	4
Loteprednol Etabonate	Loteprednol	657	0	0	0	0
Lovastatin	Lovastatin	32	0	0	0	0
Lubiprostone	Lubiprostone	59	0	0	0	0
Luliconazole	Luliconazole	40	0	0	0	0

Lurasidone	Lurasidone	486	1	1	1	0
Luteinizing Hormone	Luteinizing Hormone	129	746	466	688	126
Magnesium	Magnesium	1295	16215	8513	15043	4358
Magnesium Hydroxide	Magnesium Hydroxide	49	3306	1871	3057	556
Magnesium Oxide	Magnesium Oxide	215	2651	1459	2435	655
Malathion	Malathion	17	3	2	3	0
Mebendazole	Mebendazole	28	3	0	3	2
Meclizine	Meclizine	1022	1	1	0	0
Medroxyprogesterone	Medroxyprogesterone	11259	19901	9151	18340	5756
Mefenamic Acid	Mefenamic Acid	47	214	54	202	116
Mefloquine	Mefloquine	50	9	5	9	7
Megestrol	Megestrol	104	10	2	10	5
Meloxicam	Meloxicam	3703	0	0	0	0
Memantine	Memantine	21	0	0	0	0
Menthol	Menthol	22	42	9	40	12
Meperidine	Meperidine	361	117	24	98	80
Mercaptopurine	Mercaptopurine	105	760	500	697	154
Mesalamine	Mesalamine	1288	11	6	10	5
Mestranol	Mestranol	25	30	10	29	7
Metaxalone	Metaxalone	376	0	0	0	0
Metformin	Metformin	9252	71	31	67	44
Methadone	Methadone	31	162	34	159	56
Methenamine	Methenamine	44	3204	1899	2923	541
Methenamine Hippurate	Methenamine Hippurate	24	2	2	2	0
Methimazole	Methimazole	774	30	20	27	9
Methocarbamol	Methocarbamol	2041	140	32	130	51
Methotrexate	Methotrexate	255	77	17	76	4
Methylcobalamin	Methylcobalamin	173	189	150	178	32
Methyldopa	Methyldopa	1920	72	21	67	26
Methylene Blue	Methylene Blue	40	3210	1900	2928	541
Methylergonovine	Methylergonovine	2097	79	11	76	46
Methylphenidate	Methylphenidate	1093	1950	828	1810	322
Methylprednisolone	Methylprednisolone	13314	478	154	451	234
Metoclopramide	Metoclopramide	10286	283	94	270	146
Metolazone	Metolazone	14	2	1	2	2
Metoprolol	Metoprolol	3713	28	10	28	7
Metronidazole	Metronidazole	22674	58	14	48	28
Mg Hydroxide	Mg Hydroxide	64	1263	590	1151	328
Mg Sulf	Mg Sulf	42	17	10	15	5
Miconazole Nitrate	Miconazole	180	53	8	49	14
Miconazole	Miconazole	133	54	8	50	14
Midodrine	Midodrine	65	1	0	0	1
Milnacipran	Milnacipran	17	1	0	1	0
Mineral Oil	Mineral Oil	13	7	3	6	4
Minocycline	Minocycline	853	12	2	12	1
Mirabegron	Mirabegron	34	0	0	0	0
Mirtazapine	Mirtazapine	619	7	2	7	2
Misoprostol	Misoprostol	5959	330	19	320	250
Mn	Mn	118	43955	20582	40889	9488
Modafinil	Modafinil	162	3	2	3	1
Mometasone Furoate	Mometasone	2465	0	0	0	0
Montelukast	Montelukast	7391	4	1	2	2
Morphine	Morphine	1225	43954	20581	40888	9488
Moxifloxacin	Moxifloxacin	1112	3	0	3	0
Mupirocin	Mupirocin	11237	8	0	5	4

Mycophenolate	Mycophenolate	18	1701	651	1576	323
Mycophenolate Mofetil	Mycophenolate Mofetil	94	8	0	7	1
Na Bicarb	Na Bicarb	259	1250	585	1140	318
Na Cl	Na Cl	375	22	8	19	3
Na Pho	Na Pho	40	170	52	166	19
Na Phos	Na Phos	31	170	52	166	19
Na Sulf	Na Sulf	140	0	0	0	0
Nabumetone	Nabumetone	280	0	0	0	0
Nadolol	Nadolol	87	4	1	4	2
Naftifine	Naftifine	117	0	0	0	0
Naloxegol	Naloxegol	12	0	0	0	0
Naloxone	Naloxone	845	142	31	138	67
Naltrexone	Naltrexone	342	210	43	202	41
Naproxen	Naproxen	16935	24	9	21	12
Naratriptan	Naratriptan	160	3	1	3	2
Nebivolol	Nebivolol	205	6	2	6	2
Neomycin	Neomycin	1643	8	3	7	3
Neomycin Sulf	Neomycin Sulf	1906	0	0	0	0
Nepafenac	Nepafenac	12	0	0	0	0
Nia	Nia	34	2795	977	2648	522
Niacin	Niacin	57	5302	3469	4889	873
Niacinamide	Niacinamide	123	1314	879	1203	272
Nicotine	Nicotine	256	562	233	538	71
Nicotine Polacrilex	Nicotine Polacrilex	36	339	138	324	31
Nifedipine	Nifedipine	22127	83	35	73	35
Nitazoxanide	Nitazoxanide	16	2	0	1	2
Nitrofurantoin	Nitrofurantoin	21456	8	3	7	5
Nitrofurantoin Monohydrate	Nitrofurantoin Monohydrate	20469	8	3	7	5
Nitroglycerin	Nitroglycerin	277	43432	20349	40385	9399
Nizatidine	Nizatidine	19	2	1	2	0
Norelgestromin	Norelgestromin	3592	0	0	0	0
Norethindrone	Norethindrone	140894	617	441	580	165
Norethindrone Ace	Norethindrone Ace	20726	12	3	12	6
Norgestimate	Norgestimate	26433	0	0	0	0
Norgestrel	Norgestrel	2157	182	50	177	105
Nortriptyline	Nortriptyline	507	34	14	32	17
Nutriceutical	Nutriceutical	290	3	2	3	3
Nystatin	Nystatin	14550	30	11	30	5
Ofloxacin	Ofloxacin	2134	1376	766	1292	406
Olanzapine	Olanzapine	411	28	9	26	4
Olive Oil	Olive Oil	12	49	32	42	42
Olmesartan Medoxomil	Olmesartan Medoxomil	187	10	3	9	4
Olopatadine	Olopatadine	1353	0	0	0	0
Omalizumab	Omalizumab	89	20	8	20	10
Omega-3	Omega-3	70	5825	2575	5334	1417
Omega-3 Fatty Acids	Omega-3 Fatty Acids	238	551	449	517	245
Omega-3-Acid Ethyl Esters	Omega-3-Acid Ethyl Esters	100	66	48	63	33
Omeprazole	Omeprazole	7186	204	50	192	76
OnabotulinumtoxinA	OnabotulinumtoxinA	159	1	0	1	0
Ondansetron	Ondansetron	23496	21	1	19	15
Opium	Opium	28	1322	304	1236	553
Orphenadrine	Orphenadrine	186	140	33	130	50
Oseltamivir	Oseltamivir	9182	15	3	14	2
Oxaprozin	Oxaprozin	27	0	0	0	0

Oxazepam	Oxazepam	16	5	2	3	2
Oxcarbazepine	Oxcarbazepine	341	10	6	9	4
Oxiconazole Nitrate	Oxiconazole	77	0	0	0	0
Oxybutynin	Oxybutynin	501	1	0	1	0
Oxycodone	Oxycodone	168071	447	111	424	219
Oxymetazoline	Oxymetazoline	96	3936	1218	3635	1247
Oxymorphone	Oxymorphone	11	47	41	37	9
Oxyquinoline	Oxyquinoline	14	749	593	693	136
Paliperidone	Paliperidone	27	2	2	2	0
Pantoprazole	Pantoprazole	5258	58	15	55	26
Paroxetine	Paroxetine	1947	37	22	31	11
PEG	PEG	41	203	99	186	55
PEG Electrolyte Lavage Solution	PEG Electrolyte Lavage Solution	318	0	0	0	0
Penciclovir	Penciclovir	85	31	12	26	12
Penicillin V	Penicillin V	4032	356	132	338	108
Pentazocine	Pentazocine	55	259	62	242	133
Pentosan Polysulfate	Pentosan Polysulfate	62	0	0	0	0
Permethrin	Permethrin	697	10	6	10	4
Perphenazine	Perphenazine	12	1	0	1	0
Petrolatum	Petrolatum	19	28	13	26	14
Petrolatum, White	Petrolatum, White	18	17	8	16	8
Phenazopyridine	Phenazopyridine	2991	1	1	1	0
Phendimetrazine	Phendimetrazine	28	0	0	0	0
Phenobarb	Phenobarb	16	96	47	91	33
Phenobarbital	Phenobarbital	39	138	69	122	43
Phentermine	Phentermine	1189	0	0	0	0
Phenyleph	Phenyleph	163	81	39	79	16
Phenytoin	Phenytoin	32	137	68	121	25
Phytonadione	Phytonadione	28	253	149	247	69
Pilocarpine	Pilocarpine	16	4	2	3	0
Pimecrolimus	Pimecrolimus	443	0	0	0	0
Pindolol	Pindolol	36	7	5	7	2
Pioglitazone	Pioglitazone	36	17497	7893	16415	3035
Piroxicam	Piroxicam	42	4	2	3	2
Plecanatide	Plecanatide	29	0	0	0	0
Podofilox	Podofilox	30	124	91	118	20
Polyethylene G	Polyethylene G	19	33	12	30	12
Polyethylene Glycol 3350	Polyethylene Glycol 3350	3538	3	1	3	2
Polymyxin B	Polymyxin B	4631	1	0	1	1
Polymyxin B Sulf	Polymyxin B Sulf	1868	0	0	0	0
Polyureaurethane	Polyureaurethane	17	0	0	0	0
Potassium Bitartrate	Potassium	85	0	0	0	0
Potassium Nitrate	Potassium	424	6	1	5	3
Potassium	Potassium	2825	40472	19383	37623	8704
Pramipexole Dihydrochloride	Pramipexole	72	0	0	0	0
Pramoxine	Pramoxine	3531	3	0	3	1
Prasterone	Prasterone	19	1242	901	1131	253
Pravastatin	Pravastatin	158	142	35	133	53
Prazosin	Prazosin	297	114	68	82	16
Prednisolone	Prednisolone	1390	461	135	443	192
Prednisolone Sodium	Prednisolone	115	156	37	152	17
Prednisone	Prednisone	21563	129	35	128	15
Pregabalin	Pregabalin	309	4	1	4	2

Prenata	Prenata	1412	5468	2310	5167	808
Prilocaine	Prilocaine	1075	29	0	23	20
Prochlorperazine	Prochlorperazine	611	292	102	258	75
Progesterone	Progesterone	2041	43456	20268	40421	9403
Progestin	Progestin	623	43100	20032	40091	9298
Proguanil	Proguanil	80	3	0	3	2
Promethazine	Promethazine	8896	222	57	209	118
Propranolol	Propranolol	2557	67	25	63	17
Propylthiouracil	Propylthiouracil	147	972	295	916	249
Protease	Protease	117	42318	19737	39333	9221
PSE	PSE	3089	851	206	812	144
Pseudoephedrine	Pseudoephedrine	203	3612	1764	3283	938
Pyridostigmine Bromide	Pyridostigmine	70	11	1	11	0
Pyridoxal Phos	Pyridoxal Phos	61	30	26	26	8
Pyridoxine	Pyridoxine	1075	42207	19652	39305	9087
Quetiapine	Quetiapine	1321	15	5	14	3
Quinapril	Quinapril	16	7	3	7	3
Rabeprazole	Rabeprazole	92	56	13	53	24
Raltegravir	Raltegravir	43	110	64	108	82
Ramelteon	Ramelteon	13	1	0	1	0
Ramipril	Ramipril	47	1	0	1	0
Ranitidine	Ranitidine	3323	10	3	9	7
Reduced Diphtheria Toxoid	Reduced Diphtheria Toxoid	790	19	1	18	6
Retapamulin	Retapamulin	13	0	0	0	0
Rho(D) Immune Globulin	Rho(D) Immune Globulin	34	114	91	105	14
Rifampin	Rifampin	90	77	19	75	22
Rifaximin	Rifaximin	118	0	0	0	0
Rilpivirine	Rilpivirine	57	6	5	6	5
Rimegepant	Rimegepant	46	2	1	2	1
Risankizumab-rzaa	Risankizumab-rzaa	14	0	0	0	0
Risperidone	Risperidone	312	13	3	13	2
Ritonavir	Ritonavir	45	107	62	105	84
Rivaroxaban	Rivaroxaban	416	6	3	6	1
Rizatriptan	Rizatriptan	1733	1	1	1	1
Ropinirole	Ropinirole	132	1	1	0	0
Rosuvastatin	Rosuvastatin	336	6	5	6	1
Sacubitril	Sacubitril	67	1	0	1	0
Salicylic Acid	Salicylic Acid	101	3505	1534	3232	640
Salmeterol Xinafoate	Salmeterol Xinafoate	1900	2	0	2	0
Sapropterin Dihydrochloride	Sapropterin	21	2	1	2	1
Sch	Sch	11	8589	3175	8023	2038
Schi	Schi	38	175	61	164	12
Schizochytrium	Schizochytrium	32	4	2	3	4
Scop Hydr	Scop Hydr	16	0	0	0	0
Scopolamine	Scopolamine	377	11	2	9	7
Secnidazole	Secnidazole	52	0	0	0	0
Secukinumab	Secukinumab	49	1	0	1	0
Segesterone	Segesterone	75	0	0	0	0
Selenium Sulfide	Selenium Sulfide	168	0	0	0	0
Semaglutide	Semaglutide	169	20573	10381	19259	5831
Senna	Senna	351	21	4	21	5
Sennosides	Sennosides	387	4	2	4	3
Sennosides A and B	Sennosides A and B	125	4	2	4	3

Sertraline	Sertraline	53420	67	34	57	35
Sevelamer	Sevelamer	18	0	0	0	0
Silver Sulfadiazine	Silver Sulfadiazine	907	5	0	3	2
Simethicone	Simethicone	129	10	1	8	6
Simvastatin	Simvastatin	197	67	33	60	16
Sitagliptin	Sitagliptin	157	1	0	1	1
Sodium	Sodium	1042	43840	20541	40789	9455
Sodium Bicarbonate	Sodium Bicarbonate	36	1901	1009	1729	470
Sodium Fluoride	Sodium Fluoride	1799	383	193	362	72
Sodium Oxybate	Sodium Oxybate	34	5	4	4	1
Sodium Picosulfate	Sodium Picosulfate	197	2	1	1	2
Sofosbuvir	Sofosbuvir	44	4	1	4	1
Solifenacin	Solifenacin	41	0	0	0	0
Sotalol	Sotalol	25	44	7	44	8
Spacer, Inhalation	Spacer, Inhalation	174	6	0	5	5
Spinosad	Spinosad	35	1	0	1	1
Spironolactone	Spironolactone	1965	102	38	98	39
Succinic Acid	Succinic Acid	111	412	270	374	79
Sucralfate	Sucralfate	1130	3	0	3	0
Sulconazole Nitrate	Sulconazole	15	0	0	0	0
Sulfacetamide	Sulfacetamide	1039	0	0	0	0
Sulfamethoxazole	Sulfamethoxazole	17237	123	17	121	53
Sulfasalazine	Sulfasalazine	198	94	85	87	25
Sulfur	Sulfur	476	23046	10891	21292	5196
Sulindac	Sulindac	44	1	0	1	1
Sumatriptan	Sumatriptan	4901	9	2	7	4
Suvorexant	Suvorexant	34	0	0	0	0
Tacrolimus	Tacrolimus	527	38	18	36	6
Tamoxifen	Tamoxifen	111	15	2	14	2
Tamsulosin	Tamsulosin	1347	1	0	1	0
Tapentadol	Tapentadol	64	1	0	1	1
Tavaborole	Tavaborole	56	0	0	0	0
Tazarotene	Tazarotene	237	0	0	0	0
Telmisartan	Telmisartan	48	9	2	9	2
Temazepam	Temazepam	216	1	1	1	0
Temozolomide	Temozolomide	11	1	0	1	0
Tenofovir	Tenofovir	27	314	133	304	247
Tenofovir Al	Tenofovir Al	35	14	10	14	13
Tenofovir Alafenamide	Tenofovir Alafenamide	122	157	78	150	119
Tenofovir Di	Tenofovir Di	11	64	35	63	55
Tenofovir Disoproxil	Tenofovir Disoproxil	268	307	128	299	242
Terbinafine	Terbinafine	780	2	0	2	1
Terconazole	Terconazole	3122	0	0	0	0
Teriflunomide	Teriflunomide	15	0	0	0	0
Testosterone Cypionate	Testosterone Cypionate	16	1	1	1	0
Tetracaine	Tetracaine	31	8	0	8	8
Tetracycline	Tetracycline	83	19	6	17	5
Tezacaftor	Tezacaftor	27	6	2	6	0
Theophylline	Theophylline	14	185	97	172	48
Thonzonium Brom	Thonzonium Brom	38	0	0	0	0
Thyroid	Thyroid	2064	22195	10099	20793	4764
Ticagrelor	Ticagrelor	11	2	0	2	0
Timolol	Timolol	199	12	4	12	2
Tinidazole	Tinidazole	551	33	9	31	9
Tiotropium Bromide	Tiotropium	93	0	0	0	0
Tizanidine	Tizanidine	1478	0	0	0	0

Tobramycin	Tobramycin	3644	57	36	52	16
Tofacitinib	Tofacitinib	34	2	0	2	1
Tolterodine	Tolterodine	47	0	0	0	0
Topiramate	Topiramate	2611	17	9	16	9
Torsemide	Torsemide	57	19	8	18	8
Tramadol	Tramadol	11834	283	72	262	148
Tranexamic Acid	Tranexamic Acid	733	168	23	150	90
Travoprost	Travoprost	28	0	0	0	0
Trazodone	Trazodone	3063	4	3	3	2
Tretinoin	Tretinoin	2716	32577	15895	30143	7887
Triamcinolone Acetonide	Triamcinolone Acetonide	15568	4	1	3	2
Triamterene	Triamterene	627	85	30	82	33
Triazolam	Triazolam	372	0	0	0	0
Trifarotene	Trifarotene	20	0	0	0	0
Trifluridine	Trifluridine	19	8	8	8	1
Trimethobenzamide	Trimethobenzamide	13	3	0	3	0
Trimethoprim	Trimethoprim	20247	137	24	135	56
Ubrogepant	Ubrogepant	50	1	0	1	0
Ulipristal	Ulipristal	139	697	293	640	93
Umeclidinium	Umeclidinium	20	0	0	0	0
Urea	Urea	179	6600	3898	5997	1163
Ursodiol	Ursodiol	306	349	241	316	54
Ustekinumab	Ustekinumab	152	10	5	9	4
Valacyclovir	Valacyclovir	17017	10	4	10	6
Valganciclovir	Valganciclovir	22	8	1	8	1
Valsartan	Valsartan	392	10	3	10	3
Vancomycin	Vancomycin	363	84	50	79	26
Varenicline	Varenicline	410	0	0	0	0
Vedolizumab	Vedolizumab	14	8	4	8	7
Velpatasvir	Velpatasvir	21	4	1	4	1
Venlafaxine	Venlafaxine	3275	28	19	25	12
Verapamil	Verapamil	325	24	3	23	2
Vilanterol	Vilanterol	13	0	0	0	0
Vilanterol Trifenatate	Vilanterol	576	0	0	0	0
Vilazodone	Vilazodone	371	1	1	0	0
Vitamin	Vitamin	959	42130	19693	39173	9294
Vitamin A	Vitamin A	20	37932	18095	35204	8580
Vitamin B	Vitamin B	654	14597	6432	13719	3604
Vitamin B1	Vitamin B1	30	5518	3647	5096	912
Vitamin B12	Vitamin B12	39639	16846	8234	15519	3239
Vitamin B2	Vitamin B2	42	5639	3726	5178	942
Vitamin B3	Vitamin B3	2399	200	84	191	37
Vitamin B6	Vitamin B6	2437	5729	3781	5263	962
Vitamin C	Vitamin C	46831	10641	5954	9730	2148
Vitamin D	Vitamin D	155	22118	10242	20544	4680
Vitamin E	Vitamin E	22	4274	2470	4006	925
Voriconazole	Voriconazole	15	5	0	5	0
Vortioxetine Hydrobromide	Vortioxetine	423	3	0	3	0
Warfarin	Warfarin	631	86	12	83	17
Water, Sterile	Water, Sterile	20	1093	544	967	247
Witch Hazel	Witch Hazel	18	26	10	23	6
Zafirlukast	Zafirlukast	20	0	0	0	0
Zaleplon	Zaleplon	104	2	1	2	1
Zidovudine	Zidovudine	37	1413	355	1360	692

Zinc	Zinc	159	689	528	642	165
Zinc Oxide	Zinc Oxide	18	7448	2822	6824	1558
Ziprasidone	Ziprasidone	103	0	0	0	0
Zolmitriptan	Zolmitriptan	247	1	1	1	1
Zolpidem	Zolpidem	3310	114	64	109	11
Zonisamide	Zonisamide	174	7	5	6	4

Pediatric (0-1 Year Old) Drug Publication Frequency						
**Original Drug Name**	**Cleaned Drug Name**	**peds: 0~1 frequency**	**All publication**	**PK publication**	**PE publication**	**CT publication**
Acetaminophen	Acetaminophen	5926	52987	17206	51153	15188
Acetazolamide	Acetazolamide	49	142	28	141	36
Acetic Acid	Acetic Acid	94	38695	18202	36682	9915
Acetylcysteine	Acetylcysteine	35	9561	3871	9142	2891
Acyclovir	Acyclovir	1656	704	133	700	161
Adapalene	Adapalene	32	648	284	612	159
AHF	AHF	21	952	471	918	295
Al Hydroxide	Al Hydroxide	18	97	37	94	67
Albendazole	Albendazole	80	322	139	290	135
Albumin-Free	Albumin-Free	30	14	12	14	13
Albuterol	Albuterol	133031	709	83	699	423
Alclometasone Dipropionate	Alclometasone	3724	1	1	1	1
Aluminum Hydroxide	Aluminum Hydroxide	28	28652	14811	27058	5902
Amino Acids	Amino Acids	371	323176	139991	308338	72917
Aminocaproic Acid	Aminocaproic Acid	87	93	30	87	29
Amiodarone	Amiodarone	49	295	48	288	74
Amlodipine	Amlodipine	107	69	22	67	23
Amlodipine Besylate	Amlodipine Besylate	138	69	22	67	23
Ammonium	Ammonium	82	22912	12977	21460	4677
Amoxicillin	Amoxicillin	557516	31258	11236	29942	10680
Ampicillin	Ampicillin	13	233038	88803	222628	61271
Amylase	Amylase	422	839	527	796	113
Antihemophilic Factor Plasma	Antihemophilic Factor Plasma	30	2343	782	2244	553
Antihemophilic Factor VIII	Antihemophilic Factor VIII	13	952	471	918	295
Antipyrine	Antipyrine	153	2820	1579	2675	580
Apraclonidine	Apraclonidine	14	10	1	9	3
Ascorbic Acid	Ascorbic Acid	5908	28680	17575	27241	4422
Aspirin	Aspirin	682	31134	13384	29358	8844
Atenolol	Atenolol	90	103	19	100	54
Atorvastatin	Atorvastatin	19	1630	438	1596	638
Atovaquone	Atovaquone	1262	57	6	57	20
Atropine	Atropine	276	610	81	604	295
Atropine Sulf	Atropine Sulf	15	39	1	39	23
Azelastine	Azelastine	183	8	2	7	4
Azithromycin	Azithromycin	61774	835	96	807	496
Bacitracin	Bacitracin	1954	57	1	56	20
Bacitracin Zn	Bacitracin Zn	43	0	0	0	0
Baclofen	Baclofen	76	64	12	62	19
Barrier Skin Protectant	Barrier Skin Protectant	490	43	6	41	20
Becaplermin	Becaplermin	18	34	28	30	3
Beclomethasone Dipropionate	Beclomethasone	1889	52	7	49	44
Benzocaine	Benzocaine	163	2924	1528	2795	594
Benzonatate	Benzonatate	18	4	2	4	0

Benzoyl Peroxide	Benzoyl Peroxide	72	80	19	78	4
Besifloxacin	Besifloxacin	66	9	0	8	9
Betaine Anhydrous	Betaine Anhydrous	11	193	137	179	41
Betamethasone Dipropionate	Betamethasone	7401	9	1	9	5
Betamethasone Valerate	Betamethasone	2059	16	1	16	10
Betaxolol	Betaxolol	14	13	1	13	6
Bethanechol	Bethanechol	313	25	1	25	8
Biotin	Biotin	246	2242	1462	2133	436
Bosentan	Bosentan	57	63	14	63	29
Brompheniramine Mal	Brompheniramine Mal	7974	3	0	3	3
Budesonide	Budesonide	20517	304	50	291	234
Bumetanide	Bumetanide	25	49	24	45	25
Bupropion	Bupropion	29	58	13	52	15
Ca	Ca	105	320783	138734	306066	72533
Caffeine	Caffeine	508	65533	24859	62968	16029
Calcipotriene	Calcipotriene	11	4	0	4	1
Calcitriol	Calcitriol	193	694	463	664	142
Calcium	Calcium	24	322653	139738	307831	72853
Cannabidiol	Cannabidiol	19	123	50	117	43
Captopril	Captopril	287	302574	131090	288664	69204
Carbamide Peroxide	Carbamide Peroxide	15	1	1	1	1
Carbinoxamine	Carbinoxamine	134	3	2	3	1
Carboxymethylcellulose	Carboxymethylcellulose	213	62	20	57	25
Carvedilol	Carvedilol	43	168	57	161	51
Cefaclor	Cefaclor	66	2610	1009	2469	724
Cefadroxil	Cefadroxil	539	57	27	54	31
Cefdinir	Cefdinir	156104	159	78	147	55
Cefixime	Cefixime	3567	156	33	155	102
Cefpodoxime Proxetil	Cefpodoxime Proxetil	108	167	87	166	124
Cefprozil	Cefprozil	11705	52	22	52	39
Ceftibuten	Ceftibuten	97	16	2	16	14
Ceftriaxone	Ceftriaxone	126	1159	176	1148	365
Cefuroxime Axetil	Cefuroxime Axetil	272	358	56	354	127
Cellulose	Cellulose	208	1665	561	1496	667
Cephalexin	Cephalexin	27866	229	61	225	136
Cetirizine	Cetirizine	16177	48	12	46	32
Chlophedianol	Chlophedianol	13	0	0	0	0
Chlorhexidine	Chlorhexidine	130	313	29	293	207
Chloride	Chloride	30	255293	108946	243338	60029
Chlorothiazide	Chlorothiazide	1049	118	45	114	43
Chlorpheniramine Mal	Chlorpheniramine Mal	15	5	1	5	3
Cholecalciferol	Cholecalciferol	6209	149440	57981	142683	35174
Cholestyramine	Cholestyramine	1627	114	56	111	29
Chorionic Gonadotropin	Chorionic Gonadotropin	25	3535	2526	3354	446
Ciclopirox	Ciclopirox	185	8	0	8	2
Ciclopirox Olamine	Ciclopirox	280	7	0	7	2
Cimetidine	Cimetidine	1014	111	38	109	53
Ciprofloxacin	Ciprofloxacin	30695	1082	250	1046	226
Citalopram Hydrobromide	Citalopram	16	103	39	99	28
Citric Acid	Citric Acid	237	12446	4821	11672	3805
Clarithromycin	Clarithromycin	1700	39689	17078	37918	6896
Clavulanate	Clavulanate	127970	1762	224	1728	772
Clindamycin	Clindamycin	427	665	41	656	167
Clindamycin Palmitate	Clindamycin	6314	664	41	655	167

Clobazam	Clobazam	194	45236	16233	43620	10854
Clobetasol	Clobetasol	550	34	6	34	10
Clocortolone Pivalate	Clocortolone	237	1	0	1	1
Clonazepam	Clonazepam	262	224	56	217	54
Clonidine	Clonidine	163	374	104	369	199
Clopidogrel Hydrogen	Clopidogrel Hydrogen	31	46	11	46	20
Clotrimazole	Clotrimazole	9294	60	2	58	18
Coagulation Factor IX	Coagulation Factor IX	12	47813	19719	45547	13927
Codeine	Codeine	672	3610	1131	3483	1472
Colestipol	Colestipol	158	2	2	2	2
Colistin Sulf	Colistin Sulf	35	3	0	3	1
Collagenase	Collagenase	13	44871	18435	42510	10270
Conjugated Estrogens	Conjugated Estrogens	4003	1521	943	1416	161
Corticotropin	Corticotropin	207	2309	1326	2211	550
Crisaborole	Crisaborole	1974	2	1	2	2
Cromolyn	Cromolyn	95	177	53	175	117
Cu	Cu	365	213004	87475	203060	46927
Cyanocobalamin	Cyanocobalamin	13	30294	19026	28739	4698
Cyclobenzaprine	Cyclobenzaprine	18	5	1	5	1
Cyclopentolate	Cyclopentolate	348	74	2	71	38
Cyclosporine	Cyclosporine	30	3818	1430	3712	998
Cyproheptadine	Cyproheptadine	1073	34	5	32	13
Desloratadine	Desloratadine	57	8	4	7	5
Desmopressin	Desmopressin	22	188	68	188	40
Desonide	Desonide	14817	314	51	301	246
Desoximetasone	Desoximetasone	321	2	0	2	1
Dexamethasone Sodium	Dexamethasone	972	36	9	34	32
Dexamethasone	Dexamethasone	29132	302302	130892	288415	69155
Dexchlorpheniramine Mal	Dexchlorpheniramine Mal	13	1	0	1	0
Dexmethylphenidate	Dexmethylphenidate	11	3	1	3	1
Dextromethorphan Hydrobromide	Dextromethorphan	13	1511	478	1441	674
Dextrose	Dextrose	51	19430	10785	18579	5040
Diazepam	Diazepam	1122	67498	34177	63440	10260
Diazoxide	Diazoxide	211	331	128	328	49
Diclofenac	Diclofenac	11	1296	364	1237	619
Dicyclomine	Dicyclomine	105	44	3	42	16
Digoxin	Digoxin	830	771	273	756	158
Diphenhydram	Diphenhydram	18	97	24	92	25
diphenhydrAMINE	diphenhydrAMINE	343	4819	1422	4614	1530
Diphenoxylate	Diphenoxylate	11	20	0	20	5
Diphtheria Toxoid	Diphtheria Toxoid	241	25033	10668	23919	6278
Divalproex	Divalproex	14	1287	534	1242	417
DM Hydrobrom	DM Hydrobrom	8028	0	0	0	0
Dornase Alfa	Dornase Alfa	138	46	5	45	24
Dorzolamide	Dorzolamide	143	8	0	8	4
Doxepin	Doxepin	17	10	4	9	0
Doxycycline	Doxycycline	55	218	22	216	54
Doxycycline Hyclate	Doxycycline Hyclate	24	208	21	206	51
Econazole Nitrate	Econazole	1490	1	0	1	1
Emicizumab-kxwh	Emicizumab-kxwh	16	14	7	14	8
Emollient	Emollient	24	7939	4651	7544	2040
Enalapril	Enalapril	862	119	35	111	56
Enoxaparin	Enoxaparin	375	260	99	256	122
Epinephrine	Epinephrine	15132	2556	1012	2455	884

Epoetin Alfa	Epoetin Alfa	69	1126	744	1080	440
Epoetin Alfa-epbx	Epoetin Alfa-epbx	16	0	0	0	0
Ergocalciferol	Ergocalciferol	44	4350	3353	4192	1047
Erythromycin	Erythromycin	58007	46243	19471	44103	8626
Erythromycin Ethylsuccinate	Erythromycin Ethylsuccinate	1549	6032	2322	5680	1571
Escitalopram Oxalate	Escitalopram Oxalate	35	39	11	38	13
Esomeprazole	Esomeprazole	9445	109	32	105	56
Estradiol	Estradiol	996	322916	139906	308091	72910
Ethanol	Ethanol	309	47514	19706	45485	11343
Ethinyl Estradiol	Ethinyl Estradiol	133	171	55	156	84
Etonogestrel	Etonogestrel	13	20	4	17	15
Famotidine	Famotidine	41433	869	235	841	520
Fe	Fe	1642	311712	135185	297352	71832
Felbamate	Felbamate	12	483	126	455	113
Ferrous	Ferrous	2179	25585	16075	24305	3913
Ferrous Fum	Ferrous Fum	30	50	34	48	43
Fexofenadine	Fexofenadine	17	18	5	18	9
Filgrastim	Filgrastim	66	655	344	641	282
Filgrastim-aafi	Filgrastim-aafi	14	0	0	0	0
Flavoring Aid	Flavoring Aid	239	93	54	89	42
Flecainide	Flecainide	135	168	37	167	43
Fluconazole	Fluconazole	21803	560	74	552	139
Fludrocortisone	Fludrocortisone	159	120	50	119	15
Fluocinolone Acetonide	Fluocinolone Acetonide	14746	21	1	20	18
Fluocinonide	Fluocinonide	602	7	0	7	5
Fluoride	Fluoride	1255	140560	63684	133335	31249
Fluorometholone	Fluorometholone	41	3	0	3	1
Fluoxetine	Fluoxetine	28	228	65	217	57
Flurandrenolide	Flurandrenolide	76	0	0	0	0
Fluticasone	Fluticasone	12640	141	28	138	109
Foli	Foli	227	2907	1230	2829	757
Folic Ac	Folic Ac	359	2577	1095	2515	688
Folic Acid	Folic Acid	526	20344	10369	19467	5621
Formoterol	Formoterol	114	590	293	549	203
Formula, Infant	Formula, Infant	1805	3234	1662	2787	1482
Furosemide	Furosemide	3691	641	234	628	220
Gabapentin	Gabapentin	316	108	21	99	40
Gatifloxacin	Gatifloxacin	28	25	4	25	13
Gentamicin	Gentamicin	13440	1692	509	1655	493
Gentian Violet	Gentian Violet	13	64	7	61	13
GG	GG	12	115949	54374	109823	24819
Glucagon	Glucagon	190	604	433	561	144
Glucose Meter	Glucose Meter	101	27	25	19	2
Glycerin	Glycerin	81	314451	135953	299869	71397
Glycerol Phenylbutyrate	Glycerol Phenylbutyrate	17	32	25	32	13
Glycopyrrolate	Glycopyrrolate	144	73	11	73	52
Gramicidin	Gramicidin	336	36	8	35	13
Griseofulvin	Griseofulvin	358	57	2	57	25
Guaifenesin	Guaifenesin	111	10493	4117	9985	2964
Guanfacine	Guanfacine	16	5	0	5	1
Halobetasol	Halobetasol	30	1	0	1	0
HC	HC	2702	20292	11427	19136	3855
HC Ace	HC Ace	36	0	0	0	0
Heparin	Heparin	22	42158	21439	40420	11048
Hydrochlorothiazide	Hydrochlorothiazide	203	558	173	543	202

Hydrocodone Bitartrate	Hydrocodone	4544	2730	759	2642	1188
Hydrocortisone Sodium	Hydrocortisone	186	3195	2273	3023	801
Hydrocortisone Valerate	Hydrocortisone	2913	1	0	1	1
Hydrocortisone	Hydrocortisone	68000	8328	4001	8006	2783
Hydrocortisone Butyrate	Hydrocortisone Butyrate	1082	3195	2273	3023	801
Hydroxocobalamin	Hydroxocobalamin	15	88	46	88	10
Hydroxyurea	Hydroxyurea	35	34374	20014	32375	7152
Hydroxyzine	Hydroxyzine	8183	63	7	62	39
Hyoscyamine	Hyoscyamine	3574	32606	15248	30927	5863
Hyoscyamine Sulf	Hyoscyamine Sulf	15	2	0	2	0
Ibuprofen	Ibuprofen	8726	39081	17410	36779	7580
Imiquimod	Imiquimod	26	43	1	41	18
Immune Globulin	Immune Globulin	24	273030	124926	259879	64861
Insulin Aspart	Insulin Aspart	17	2354	1796	2214	395
Insulin Glargine	Insulin Glargine	20	42	24	42	29
Insulin Lispro	Insulin Lispro	14	44	29	44	29
Iodoquinol	Iodoquinol	52	9	3	9	2
Ipratropium Bromide	Ipratropium	1943	61	1	61	47
Iron	Iron	281	316314	137135	301745	72334
Isoniazid	Isoniazid	37	26161	10647	24837	7992
Isopropyl Alcohol	Isopropyl Alcohol	44	7122	1950	6909	1390
Isradipine	Isradipine	20	18	6	18	14
Itraconazole	Itraconazole	19	308	75	300	92
Ivacaftor	Ivacaftor	13	25	7	23	7
Ivermectin	Ivermectin	86	268	26	258	115
K Phos	K Phos	15	35	25	26	12
Ketoconazole	Ketoconazole	20129	145	34	141	37
Ketorolac Tromethamine	Ketorolac Tromethamine	12	101	18	99	80
Ketotifen	Ketotifen	12	65	14	64	42
Lacosamide	Lacosamide	101	52	18	51	25
Lactobacillus rhamnosus GG	Lactobacillus rhamnosus GG	30	229	45	222	157
Lactulose	Lactulose	10935	240	168	237	115
Lamivudine	Lamivudine	12	706	241	701	504
Lamotrigine	Lamotrigine	13	303	93	293	134
Lancet	Lancet	316	30	11	27	9
Lansoprazole	Lansoprazole	9693	54	12	52	30
Latanoprost	Latanoprost	82	18	2	16	12
Leucovorin	Leucovorin	40	261	120	259	117
Levalbuterol	Levalbuterol	5489	7	1	7	7
Levetiracetam	Levetiracetam	2303	367	93	362	194
Levocarnitine	Levocarnitine	393	1622	1320	1484	164
Levocetirizine Dihydrochloride	Levocetirizine	690	13	4	13	10
Levofloxacin	Levofloxacin	205	212	25	211	40
Levomefolate Ca	Levomefolate Ca	1309	0	0	0	0
Levonorgestrel	Levonorgestrel	20	152	34	140	81
Levothyroxine	Levothyroxine	2549	8878	6319	8451	1139
Lidocaine	Lidocaine	2977	851	163	814	483
Linezolid	Linezolid	74	198	36	193	48
Lipase	Lipase	422	318433	139095	303694	71920
Lisdexamfetamine Dimesylate	Lisdexamfetamine Dimesylate	26	10	1	10	1
Lisinopril	Lisinopril	109	93	33	89	33
Loperamide	Loperamide	13	67	9	65	31
Loratadine	Loratadine	667	28	6	27	18

Lorazepam	Lorazepam	274	145	38	135	69
Losartan	Losartan	26	119	39	111	49
Loteprednol Etabonate	Loteprednol	72	1	0	1	1
Lubricant	Lubricant	327	3999	1675	3843	999
Magnesium	Magnesium	12	118440	56601	112872	34925
Magnesium Hydroxide	Magnesium Hydroxide	38	28718	14846	27121	5923
Mebendazole	Mebendazole	17	88	14	88	39
Mefloquine	Mefloquine	1129	612	112	602	470
Meloxicam	Meloxicam	11	3	1	2	1
Menthol	Menthol	21	415	70	397	71
Mercaptopurine	Mercaptopurine	17	6388	3398	6048	1560
Mesalamine	Mesalamine	65	105	15	103	44
Metformin	Metformin	29	552	187	534	290
Methadone	Methadone	167	927	190	915	332
Methotrexate	Methotrexate	11	2127	555	2103	1044
Methylphenidate	Methylphenidate	32	29304	12751	27931	6088
Methylprednisolone	Methylprednisolone	41	4167	1226	4064	2089
Metoclopramide	Metoclopramide	818	1366	377	1306	694
Metoprolol	Metoprolol	16	166	48	161	67
Metronidazole	Metronidazole	719	668	92	655	247
Mg Hydroxide	Mg Hydroxide	18	12331	4720	11567	3840
Miconazole Nitrate	Miconazole	631	1522	263	1503	456
Midazolam	Midazolam	62	1098	221	1071	693
Mometasone Furoate	Mometasone	8966	23	4	23	12
Montelukast	Montelukast	7664	91	20	88	61
Morphine	Morphine	175	323152	139983	308314	72916
Moxifloxacin	Moxifloxacin	16012	78	11	76	21
Mupirocin	Mupirocin	77126	106	1	103	47
Mycophenolate Mofetil	Mycophenolate Mofetil	53	324	112	319	151
Na Fluoride	Na Fluoride	13683	0	0	0	0
Na Phos	Na Phos	24	2574	693	2546	940
Nadolol	Nadolol	23	32	8	32	16
Naftifine	Naftifine	21	0	0	0	0
Naloxone	Naloxone	16	565	93	551	262
Naproxen	Naproxen	33	186	91	170	31
Nebulizer, Direct Patient Interface	Nebulizer, Direct Patient Interface	463	0	0	0	0
Neomycin	Neomycin	4317	198	25	195	82
Neomycin Sulf	Neomycin Sulf	2738	16	2	16	8
Nitazoxanide	Nitazoxanide	67	20	1	17	18
Nitrofurantoin	Nitrofurantoin	1128	164	9	161	38
Nitrofurantoin Monohydrate	Nitrofurantoin Monohydrate	14	161	9	158	38
Nizatidine	Nizatidine	4245	7	2	7	5
Norethindrone	Norethindrone	49	2040	1435	1828	461
Norethindrone Ace	Norethindrone Ace	30	19	2	16	11
Norgestimate	Norgestimate	34	2	0	1	2
Nystatin	Nystatin	205574	545	177	528	105
Ofloxacin	Ofloxacin	27270	6225	3135	6011	1827
Olopatadine	Olopatadine	358	0	0	0	0
Omeprazole	Omeprazole	11527	1939	501	1899	806
Ondansetron	Ondansetron	25957	187	24	187	132
Oseltamivir	Oseltamivir	41507	437	46	428	132
Ostomy Product	Ostomy Product	28	0	0	0	0
Oxcarbazepine	Oxcarbazepine	369	127	38	124	54
Oxiconazole Nitrate	Oxiconazole	199	0	0	0	0

Oxybutynin	Oxybutynin	1576	40	2	38	22
Oxycodone	Oxycodone	3132	3289	917	3186	1508
Ozenoxacin	Ozenoxacin	15	2	0	2	2
Palivizumab	Palivizumab	6594	533	44	517	250
Pantoprazole	Pantoprazole	138	117	25	112	64
Pegfilgrastim	Pegfilgrastim	19	10	6	10	8
Penicillin V	Penicillin V	724	4340	1560	4108	1483
Permethrin	Permethrin	1357	155	44	144	72
Petrolatum	Petrolatum	117	155	29	149	68
Petrolatum, White	Petrolatum, White	606	81	18	78	42
Phenobarb	Phenobarb	15	1275	519	1241	495
Phenobarbital	Phenobarbital	1422	1673	677	1591	587
Phenyleph	Phenyleph	208	590	145	570	287
Phenylephrine	Phenylephrine	45	11423	5238	10169	2543
Phenytoin	Phenytoin	41	1461	652	1388	317
Phytonadione	Phytonadione	126	924	435	903	200
Pilocarpine	Pilocarpine	11	49	26	46	1
Pimecrolimus	Pimecrolimus	1013	46	7	45	38
Polyethylene Glycol 3350	Polyethylene Glycol 3350	2495	18	4	18	14
Polymyxin B	Polymyxin B	72407	66	7	65	36
Polymyxin B Sulf	Polymyxin B Sulf	2703	9	0	9	7
Potassium Nitrate	Potassium	18	75	6	74	25
Potassium	Potassium	456	283823	125787	270270	64863
Potassium Iodide	Potassium Iodide	28	88970	44032	84146	18450
Pramoxine	Pramoxine	48	6	1	6	5
Prednicarbate	Prednicarbate	97	4	0	4	3
Prednisolone	Prednisolone	38237	4572	1270	4493	2021
Prednisolone Sodium	Prednisolone	56506	2464	623	2443	898
Prednisone	Prednisone	902	2269	542	2260	951
Prilocaine	Prilocaine	2235	170	18	156	105
Proguanil	Proguanil	1254	118	25	117	66
Promethazine	Promethazine	133	1253	341	1200	632
Propafenone	Propafenone	11	64	15	59	22
Proparacaine	Proparacaine	46	16	0	16	15
Propranolol	Propranolol	6361	1327	241	1309	437
Protease	Protease	422	306618	131866	292376	69919
PSE	PSE	7950	11099	2961	10877	3371
Pyridoxine	Pyridoxine	18	304265	132131	290279	69483
Rabeprazole	Rabeprazole	56	105	18	101	63
Ranitidine	Ranitidine	140146	158	39	154	83
Retapamulin	Retapamulin	35	11	0	9	8
Rifampin	Rifampin	39	1253	210	1240	372
Saccharomyces Boulardii	Saccharomyces Boulardii	12	50	5	49	41
Salicylic Acid	Salicylic Acid	18	29534	12070	27936	7402
Salmeterol Xinafoate	Salmeterol Xinafoate	102	14	1	13	12
Sapropterin Dihydrochloride	Sapropterin	32	47	32	45	22
Scop Hydr	Scop Hydr	15	0	0	0	0
Scopolamine	Scopolamine	13	66	13	63	39
Selenium Sulfide	Selenium Sulfide	609	7	0	7	6
Sertraline	Sertraline	63	106	41	95	46
Sevelamer	Sevelamer	12	7	5	7	4
Sildenafil	Sildenafil	409	341	52	324	161
Silver Nitrate	Silver	18	76	6	75	25
Silver Sulfadiazine	Silver Sulfadiazine	5024	87	6	78	43
Simethicone	Simethicone	108	193	47	182	143

Sirolimus	Sirolimus	72	431	125	420	137
Sodium	Sodium	18754	321128	139302	306381	72370
Sodium Bicarbonate	Sodium Bicarbonate	1869	22050	10139	20850	6518
Sodium Fluoride	Sodium Fluoride	18828	3071	1461	2915	706
Sodium Polystyrene Sulfonate	Sodium Polystyrene Sulfonate	92	18	6	18	3
Somatropin, E-Coli Derived	Somatropin, E-Coli Derived	170	48	14	46	41
Sorbitol	Sorbitol	49	240	112	226	60
Sotalol	Sotalol	139	665	180	647	121
Spacer, Inhalation	Spacer, Inhalation	7931	90	20	87	55
Spinosad	Spinosad	49	1	0	1	1
Spironolactone	Spironolactone	604	756	279	739	248
Steviol Glycosides	Steviol Glycosides	11	5	3	5	0
Sucralfate	Sucralfate	1096	25	0	25	8
Sucrose	Sucrose	407	20541	12391	19497	2837
Sulfacetamide	Sulfacetamide	2634	11	0	11	5
Sulfamethoxazole	Sulfamethoxazole	26526	2267	265	2247	738
Sulfur	Sulfur	14	151335	68073	143390	36989
Tacrolimus	Tacrolimus	1272	944	398	936	394
Tamsulosin	Tamsulosin	14	9	0	8	4
Terbinafine	Terbinafine	11	45	3	45	18
Terconazole	Terconazole	12	0	0	0	0
Testosterone Cypionate	Testosterone Cypionate	127	4	2	4	1
Testosterone Enanthate	Testosterone Enanthate	39	22	4	21	15
Tetanus Toxoid	Tetanus Toxoid	34	76451	39688	72329	15639
Tetracaine	Tetracaine	33	93	8	93	60
Theophylline	Theophylline	15	2197	976	2092	613
Thonzonium Brom	Thonzonium Brom	35	0	0	0	0
Timolol	Timolol	7281	209	31	204	79
Tobramycin	Tobramycin	18796	916	410	878	245
Topiramate	Topiramate	215	367	126	356	201
Tramadol	Tramadol	26	1571	418	1494	754
Trazodone	Trazodone	18	14	3	13	5
Tretinoin	Tretinoin	110	239623	106522	228032	61000
Triamcinolone Acetonide	Triamcinolone Acetonide	60541	177	10	176	69
Trimethoprim	Trimethoprim	93636	2454	326	2422	789
Triprolidine	Triprolidine	66	22185	11598	20871	5050
Tropicamide	Tropicamide	25	57	1	53	32
Ursodiol	Ursodiol	453	6327	3218	6013	1793
Valacyclovir	Valacyclovir	39	63	22	62	26
Valganciclovir	Valganciclovir	257	146	39	143	55
Valproic Acid	Valproic Acid	20	1286	534	1241	417
Vancomycin	Vancomycin	69	1536	461	1507	383
Vigabatrin	Vigabatrin	308	363	62	357	185
Vita	Vita	708	59602	29848	56584	12491
Vitamin	Vitamin	1111	310222	132760	296022	72192
Vitamin A	Vitamin A	11246	279011	121304	266010	66665
Vitamin B1	Vitamin B1	1105	26759	16688	25476	3988
Vitamin B12	Vitamin B12	10845	120269	52190	114426	24781
Vitamin B2	Vitamin B2	9983	29018	18122	27512	4502
Vitamin B3	Vitamin B3	9986	1169	478	1124	240
Vitamin B6	Vitamin B6	9983	29654	18418	28123	4623
Vitamin C	Vitamin C	16643	68156	34730	64305	13984
Vitamin D	Vitamin D	9862	165755	68045	158220	38566
Von Willebrand Factor	Von Willebrand Factor	21	1231	707	1185	279

Voriconazole	Voriconazole	23	176	54	169	49
Warfarin	Warfarin	14	457	77	452	107
Water	Water	786	85074	31853	80661	21852
Water, Sterile	Water, Sterile	551	6177	2769	5671	1459
Zidovudine	Zidovudine	122	5617	1706	5437	2572
Zinc	Zinc	24	3103	2286	2965	906
Zinc Oxide	Zinc Oxide	734	41236	15622	39011	8728
Zonisamide	Zonisamide	49	101	42	98	45
**Pediatric (1-12 Years Old) Drug Publication Frequency**						
**Original Drug Name**	**Cleaned Drug Name**	**peds: 1~12 frequency**	**All publication**	**PK publication**	**PE publication**	**CT publication**
1,1,1,3,3-Pentafluoropropane	1,1,1,3,3-Pentafluoropropane	58	473	43	468	236
Abacavir	Abacavir	249	1418	545	1348	431
Abatacept	Abatacept	121	114	28	111	55
AbobotulinumtoxinA	AbobotulinumtoxinA	26	29	2	28	19
Acarbose	Acarbose	20	17	13	16	3
Acebutolol	Acebutolol	11	61	24	58	15
Acesulfame	Acesulfame	12	33	10	31	4
Acetaminophen	Acetaminophen	204345	78831	22560	76273	18959
Acetazolamide	Acetazolamide	2659	340	44	339	45
Acetic Acid	Acetic Acid	4463	64270	28556	61618	15218
Acetylcysteine	Acetylcysteine	361	14323	5726	13725	4056
Acitretin	Acitretin	54	190	16	189	22
Acrivastine	Acrivastine	22	5	0	3	5
Acyclovir	Acyclovir	47932	1044	158	1041	259
Adalimumab	Adalimumab	3610	541	98	537	291
Adapalene	Adapalene	38268	1115	456	1056	269
AHF	AHF	181	2097	1020	2036	599
AHF VIII Sucrose Formulated	AHF VIII Sucrose Formulated	84	0	0	0	0
Al Hydroxide	Al Hydroxide	1515	173	69	165	105
Albendazole	Albendazole	8300	1363	315	1304	519
Albumin-Free	Albumin-Free	460	36	21	36	27
Albuterol	Albuterol	1684446	1789	216	1707	1182
Alcaftadine	Alcaftadine	477	1	0	1	1
Alclometasone Dipropionate	Alclometasone	17177	5	1	5	4
Alendronate	Alendronate	115	102	31	100	54
Alfuzosin	Alfuzosin	13	5	1	5	2
Alglucosidase Alfa	Alglucosidase Alfa	14	66	22	66	22
Allopurinol	Allopurinol	334	390	147	382	137
Almond Oil	Almond Oil	99	8	0	8	4
Almotriptan	Almotriptan	131	6	3	6	4
Aloe	Aloe	96	83461	31598	80121	22127
Aloe Vera	Aloe Vera	35	15848	5773	15124	2670
Alprazolam	Alprazolam	3171	45	9	43	7
Alteplase	Alteplase	34	32254	11558	31048	7312
Aluminum	Aluminum	4074	483203	186681	464560	103454
Aluminum Hydroxide	Aluminum Hydroxide	276	45738	23019	43592	9049
Amantadine	Amantadine	3545	122	8	121	57
Ambrisentan	Ambrisentan	191	15	5	13	8
Amcinonide	Amcinonide	28	0	0	0	0
Amikacin	Amikacin	17	715	148	703	217
Amiloride	Amiloride	183	749	256	713	174

Amino Acids	Amino Acids	261	483787	186843	465133	103500
Aminocaproic Acid	Aminocaproic Acid	1543	151	44	145	54
Amiodarone	Amiodarone	75	262	50	256	80
Amitriptyline	Amitriptyline	13667	216	35	208	66
Amlodipine	Amlodipine	364	127	41	121	43
Amlodipine Besylate	Amlodipine Besylate	2893	127	41	121	43
Ammonium	Ammonium	7040	35209	16818	33581	7209
Amoxicillin	Amoxicillin	5172956	49907	17248	47970	15787
Amphetamine	Amphetamine	19741	252985	85266	240686	71199
Amphotericin B	Amphotericin B	23	99001	41206	94591	23387
Ampicillin	Ampicillin	857	363163	123292	349769	91784
Amylase	Amylase	4546	1322	850	1254	172
Anakinra	Anakinra	227	507	278	495	102
Anastrozole	Anastrozole	1035	44	12	44	17
Anthralin	Anthralin	109	29	1	29	14
Antibacterial	Antibacterial	36	40215	10014	39018	9793
Antihemophilic Factor Plasma	Antihemophilic Factor Plasma	460	3803	1453	3668	882
Antihemophilic Factor VIII	Antihemophilic Factor VIII	96	2099	1021	2038	601
Antihemophilic Factor VIII Fc Fusion Protein Recom	Antihemophilic Factor VIII Fc Fusion Protein Recom	97	0	0	0	0
Antihemophilic Factor VIII Pegylated	Antihemophilic Factor VIII Pegylated	63	0	0	0	0
Antipyrine	Antipyrine	2144	7148	3386	6784	1650
APAP	APAP	134	70415	20865	68162	16302
Apixaban	Apixaban	40	28	12	26	12
Apraclonidine	Apraclonidine	153	11	1	11	4
Apremilast	Apremilast	12	6	1	5	2
Aprepitant	Aprepitant	281	191	53	182	49
Arformoterol	Arformoterol	26	1	1	1	1
Arginine	Arginine	68	483786	186842	465132	103500
Aripiprazole	Aripiprazole	32423	297	48	291	135
Armodafinil	Armodafinil	37	49	3	49	26
Artemether	Artemether	28	735	138	719	550
Ascorbic Acid	Ascorbic Acid	7846	50136	26194	48272	7030
Ascorbyl	Ascorbyl	15	1624	881	1584	371
Asenapine	Asenapine	585	8	3	8	6
Asfotase Alfa	Asfotase Alfa	72	36	15	34	10
Aspirin	Aspirin	2764	51565	21326	49276	14023
Atazanavir	Atazanavir	50	648	137	630	184
Atenolol	Atenolol	3112	103	21	98	51
Atomoxetine	Atomoxetine	51964	520	38	506	322
Atorvastatin	Atorvastatin	954	1722	448	1695	471
Atovaquone	Atovaquone	19676	94	13	92	37
Atropine	Atropine	13499	1292	165	1279	733
Atropine Sulf	Atropine Sulf	226	56	3	55	35
Azathioprine	Azathioprine	1174	3685	1566	3547	804
Azelaic Acid	Azelaic Acid	1513	16	5	15	10
Azelastine	Azelastine	62092	67	5	57	55
Azithromycin	Azithromycin	1385100	1336	162	1303	684
Aztreonam	Aztreonam	284	130	31	126	69
Bacitracin	Bacitracin	10850	114	2	109	41
Bacitracin Zn	Bacitracin Zn	454	0	0	0	0
Baclofen	Baclofen	5853	360	17	351	119

Baloxavir Marboxil	Baloxavir	1395	30	5	30	24
Balsalazide Disodium	Balsalazide	387	3	1	3	3
Barrier Skin Protectant	Barrier Skin Protectant	2877	197	8	176	93
Beclomethasone Dipropionate	Beclomethasone	189424	321	82	306	287
Beclomethasone Dipropionate Monohydrate	Beclomethasone Dipropionate Monohydrate	25	1	0	1	1
Benazepril	Benazepril	43	12	1	11	7
Benralizumab	Benralizumab	24	18	4	14	13
Benzalkonium	Benzalkonium	22	288	33	278	155
Benzocaine	Benzocaine	2187	7693	3445	7304	1792
Benzoin Compound	Benzoin Compound	26	1	0	1	0
Benzonatate	Benzonatate	38238	7	3	7	0
Benzoyl Peroxide	Benzoyl Peroxide	47942	217	29	203	88
Benztropine Mesylate	Benztropine	1372	21	1	21	5
Benzyl Alcohol	Benzyl Alcohol	1244	5453	1900	5285	1902
Bepotastine Besilate	Bepotastine	1592	7	0	5	7
Besifloxacin	Besifloxacin	842	10	0	9	10
Betaine	Betaine	53	2037	1216	1927	559
Betaine Anhydrous	Betaine Anhydrous	112	214	156	206	42
Betamethasone Dipropionate	Betamethasone	56342	37	2	36	25
Betamethasone Valerate	Betamethasone	14221	46	0	46	33
Betaxolol	Betaxolol	74	18	8	18	7
Bethanechol	Bethanechol	580	24	1	24	6
Bi Subcitrate K	Bi Subcitrate K	14	0	0	0	0
Bicalutamide	Bicalutamide	66	10	2	10	2
Bictegravir	Bictegravir	46	4	2	4	4
Bif	Bif	252	1291	353	1231	475
Bifidobacterium breve	Bifidobacterium breve	254	51	14	47	41
Bifidobacterium infantis	Bifidobacterium infantis	256	16220	4614	15769	2325
Bifidobacterium lactis	Bifidobacterium lactis	46	118	15	113	110
Bimatoprost	Bimatoprost	80	7	0	7	5
Bioflavonoid	Bioflavonoid	19	1645	888	1604	379
Biotin	Biotin	3014	3364	2185	3245	708
Bisacodyl	Bisacodyl	589	14631	5584	13949	3395
Bismuth Tribromophenate	Bismuth	34	0	0	0	0
Bisoprolol	Bisoprolol	41	22	3	20	8
Bosentan	Bosentan	190	119	31	117	70
Brexpiprazole	Brexpiprazole	134	6	3	5	2
Brimonidine	Brimonidine	674	23	2	23	9
Brinzolamide	Brinzolamide	266	9	0	9	5
Brivaracetam	Brivaracetam	549	27	9	25	19
Bromfenac	Bromfenac	103	3	0	3	2
Bromocriptine Mesylate	Bromocriptine	52	177	83	177	35
Brompheniramine Mal	Brompheniramine Mal	537825	7	0	7	7
Budesonide	Budesonide	224237	946	241	923	763
Bumetanide	Bumetanide	180	54	16	52	29
Buprenorphine	Buprenorphine	18	202	19	198	61
Bupropion	Bupropion	8777	93	12	89	27
Burosumab-twza	Burosumab-twza	124	46	25	46	27
Buspirone	Buspirone	9054	32	3	30	17
Butalbital	Butalbital	1587	66347	19424	64338	14952
Butenafine	Butenafine	34	3	0	3	1

Butylated Hydroxytoluene	Butylated Hydroxytoluene	32	40	28	39	11
C1 Esterase Inhibitor	C1 Esterase Inhibitor	44	268	171	252	56
C30-45 Alkyl Cetearyl Dim	C30-45 Alkyl Cetearyl Dim	17	0	0	0	0
Ca	Ca	341	479359	184877	460837	102843
Ca Ascorbate	Ca Ascorbate	68	1	0	1	1
Ca Cl	Ca Cl	18	81	24	76	13
Ca Pantothenate	Ca Pantothenate	67	0	0	0	0
Cabergoline	Cabergoline	27	65	32	63	6
Caff	Caff	17	4224	1088	4120	1381
Caffeine	Caffeine	1592	100953	34417	97286	21741
Calcipotriene	Calcipotriene	2567	60	3	60	33
Calcitonin	Calcitonin	16	5011	3996	4876	662
Calcitriol	Calcitriol	1817	1296	759	1263	346
Calcium	Calcium	319	482447	186313	463823	103365
Canakinumab	Canakinumab	331	189	29	187	74
Candesartan Cilexetil	Candesartan Cilexetil	34	16	7	14	9
Candida Albicans Antigen	Candida Albicans Antigen	13	633	162	610	81
Cannabidiol	Cannabidiol	1564	296	95	285	118
Cantharidin	Cantharidin	24	32	1	31	16
Capsaicin	Capsaicin	13	245	151	228	16
Captopril	Captopril	186	430076	165783	413588	93786
Carbamazepine	Carbamazepine	1794	1880	818	1849	708
Carbamide Peroxide	Carbamide Peroxide	246	24	2	22	7
Carbidopa	Carbidopa	660	127	55	127	35
Carbinoxamine	Carbinoxamine	7848	6	2	6	2
Carboxymethylcellulose	Carboxymethylcellulose	979	67	26	58	31
Cariprazine	Cariprazine	159	5	4	5	2
Carisoprodol	Carisoprodol	23	14998	6321	14306	2544
Carvedilol	Carvedilol	476	460	107	438	94
Castor Oil	Castor Oil	14	35	17	31	7
Cefaclor	Cefaclor	781	5223	1800	4938	1323
Cefadroxil	Cefadroxil	28892	112	43	108	55
Cefazolin	Cefazolin	20	405	105	398	152
Cefdinir	Cefdinir	1379698	146	60	144	72
Cefepime	Cefepime	23	193	27	188	67
Cefixime	Cefixime	23539	244	45	242	136
Cefpodoxime Proxetil	Cefpodoxime Proxetil	1454	194	94	193	134
Cefprozil	Cefprozil	104064	84	28	84	62
Ceftazidime	Ceftazidime	18	752	152	744	245
Ceftibuten	Ceftibuten	463	28	5	28	22
Ceftriaxone	Ceftriaxone	807	1673	196	1661	425
Cefuroxime Axetil	Cefuroxime Axetil	19065	472	55	468	148
Celecoxib	Celecoxib	1338	69	10	68	32
Cellulose	Cellulose	971	2765	745	2516	1069
Cenobamate	Cenobamate	31	2	2	2	1
Cephalexin	Cephalexin	680615	345	77	340	175
Cetirizine	Cetirizine	230295	173	25	167	113
Chlophedianol	Chlophedianol	1146	0	0	0	0
Chloral Hydrate	Chloral Hydrate	50	342	19	340	198
Chlorcyclizine	Chlorcyclizine	18	12	2	11	4
Chlordiazepoxide	Chlordiazepoxide	47	1379	588	1305	347
Chlorhexidine	Chlorhexidine	33362	573	13	533	392
Chloride	Chloride	113	391387	151191	375567	87025

Chloroquine	Chloroquine	274	1852	321	1842	737
Chlorothiazide	Chlorothiazide	835	163	69	160	53
Chloroxylenol	Chloroxylenol	20	4	2	3	0
Chlorpheniramine Polistirex	Chlorpheniramine	2517	0	0	0	0
Chlorpheniramine	Chlorpheniramine	24	20072	9687	18398	2726
Chlorpheniramine Mal	Chlorpheniramine Mal	775	20	2	18	13
Chlorpromazine	Chlorpromazine	556	241	81	236	100
Chlorthalidone	Chlorthalidone	106	112	22	107	55
Chlorzoxazone	Chlorzoxazone	68	1780	417	1731	847
Cholecalciferol	Cholecalciferol	13042	226488	79424	217586	48491
Cholestyramine	Cholestyramine	2761	147	78	145	38
Chorionic Gonadotropin	Chorionic Gonadotropin	24	2123	1340	2017	284
Ciclesonide	Ciclesonide	4247	45	15	41	42
Ciclopirox	Ciclopirox	6519	26	0	25	11
Ciclopirox Olamine	Ciclopirox	3638	25	0	24	11
Cidofovir	Cidofovir	21	234	31	229	76
Cimetidine	Cimetidine	13083	197	68	194	83
Cinacalcet	Cinacalcet	31	88	49	83	28
Ciprofloxacin	Ciprofloxacin	481412	1846	370	1798	401
Citalopram Hydrobromide	Citalopram	13841	186	31	182	66
Citric Acid	Citric Acid	2114	20788	8347	19864	6176
Citrulline	Citrulline	15	106079	43752	101978	24185
Clarithromycin	Clarithromycin	34798	55574	20712	52847	9288
Clascoterone	Clascoterone	21	6	1	6	5
Clavulanate	Clavulanate	1171001	2905	302	2859	1169
Clemastine	Clemastine	66	1795	415	1744	859
Clidinium Bromide	Clidinium	34	12	6	9	3
Clindamycin	Clindamycin	128667	958	45	942	259
Clindamycin Palmitate	Clindamycin	100074	956	45	940	259
Clioquinol	Clioquinol	94	26	1	26	6
Clobazam	Clobazam	7656	63111	20882	60960	13923
Clobetasol	Clobetasol	30126	115	8	113	55
Clocortolone Pivalate	Clocortolone	2884	3	0	3	3
Clomipramine	Clomipramine	476	95	19	94	32
Clonazepam	Clonazepam	12444	377	87	372	86
Clonidine	Clonidine	94787	900	398	892	441
Clopidogrel Hydrogen	Clopidogrel Hydrogen	196	80	15	78	30
Clorazepate Dipotassium	Clorazepate	329	15	3	15	3
Clotrimazole	Clotrimazole	61062	86	2	85	33
Clozapine	Clozapine	33	158	30	158	38
Coagulation Factor IX	Coagulation Factor IX	198	75327	27440	72330	20612
Coagulation Factor VIIa	Coagulation Factor VIIa	56	417	124	413	170
Coal Tar	Coal Tar	11	140962	34858	137764	55210
Cobamamide	Cobamamide	111	1235	926	1202	160
Cobicistat	Cobicistat	84	16	9	13	12
Codeine	Codeine	83954	4088	1055	3991	1393
Codeine Phos	Codeine Phos	1966	16	1	16	7
Coenzyme Q10	Coenzyme Q10	67	302	186	291	74
Colchicine	Colchicine	805	505	85	496	102
Colesevelam	Colesevelam	86	2	1	2	1
Colestipol	Colestipol	255	18	16	18	16
Colistimethate	Colistimethate	82	23	5	23	17
Colistin Sulf	Colistin Sulf	1736	4	0	4	2
Collagenase	Collagenase	233	69169	26327	65433	15368

Collodion	Collodion	48	15	8	15	2
Colloidal Sulfur	Colloidal Sulfur	255	198	19	198	7
Conjugated Estrogens	Conjugated Estrogens	7981	2368	1476	2272	350
Corticotropin	Corticotropin	69	3591	2082	3492	763
Cosyntropin	Cosyntropin	23	225	155	219	98
Cr	Cr	49	357249	147313	342857	75805
Crisaborole	Crisaborole	31408	24	2	22	21
Crizotinib	Crizotinib	21	55	10	53	14
Cromolyn	Cromolyn	5861	626	126	605	415
Crotamiton	Crotamiton	28	8	0	8	5
Cu	Cu	4520	328099	121480	315216	67439
Curcumin	Curcumin	37	30	5	28	19
Cyanocobalamin	Cyanocobalamin	315	52046	27396	50153	7696
Cyclobenzaprine	Cyclobenzaprine	4045	6	2	6	1
Cyclopentolate	Cyclopentolate	4514	167	3	158	84
Cyclophosphamide	Cyclophosphamide	95	370230	146447	356095	86109
Cyclosporine	Cyclosporine	1389	7446	2771	7270	2045
Cyproheptadine	Cyproheptadine	77763	117	20	112	62
Cysteamine	Cysteamine	67	181245	92013	173312	47847
Cysteamine Bitartrate	Cysteamine	104	132	64	129	47
Cytarabine	Cytarabine	103	2045	317	2033	953
Dabrafenib Mesylate	Dabrafenib	52	31	5	31	9
Dantrolene	Dantrolene	69	143	22	143	19
Dapsone	Dapsone	4987	23230	6481	22737	4062
Darbepoetin Alfa	Darbepoetin Alfa	84	90	46	88	39
Darifenacin Hydrobromide	Darifenacin	35	0	0	0	0
Darunavir Ethanolate	Darunavir Ethanolate	22	68	33	64	49
Dasatinib	Dasatinib	71	95	15	95	28
Deferasirox	Deferasirox	486	221	151	220	138
Deferiprone	Deferiprone	54	181	123	180	105
Deferoxamine Mesylate	Deferoxamine	17	610	387	609	202
Deflazacort	Deflazacort	289	100	24	100	60
Deoxy-D-Glucose	Deoxy-D-Glucose	13	1840	659	1780	99
Dermatophagoides Farinae Ext	Dermatophagoides Farinae Ext	18	8	0	7	5
Dermatophagoides Pter	Dermatophagoides Pter	18	632	345	593	171
Desipramine	Desipramine	70	139278	43197	135420	57769
Desloratadine	Desloratadine	4781	47	9	41	35
Desmopressin	Desmopressin	48110	714	205	705	330
Desogestrel	Desogestrel	654	64	6	62	29
Desonide	Desonide	65271	960	242	937	777
Desoximetasone	Desoximetasone	8573	9	0	9	5
Desvenlafaxine	Desvenlafaxine	811	32	14	32	9
Dexamethasone Sodium	Dexamethasone	4950	32	5	32	27
Dexamethasone	Dexamethasone	518079	429112	165216	412682	93594
Dexbrompheniramine	Dexbrompheniramine	322	36	3	36	1
Dexchlorpheniramine	Dexchlorpheniramine	235	15	4	15	6
Dexchlorpheniramine Mal	Dexchlorpheniramine Mal	1337	3	0	3	2
Dexlansoprazole	Dexlansoprazole	151	4	1	4	3
Dexmedetomidine	Dexmedetomidine	20	862	160	842	616
Dexmethylphenidate	Dexmethylphenidate	170620	104	11	101	84
Dextroamphetamine	Dextroamphetamine	8683	534	77	520	273
Dextromethorphan Polistirex	Dextromethorphan	102	109	37	104	32

Dextromethorphan Hydrobromide	Dextromethorphan	565	3026	880	2910	1273
Dextrose	Dextrose	400	26902	15080	25608	6312
Diazepam	Diazepam	53089	102511	45562	97493	14978
Diazoxide	Diazoxide	303	222	86	221	48
Dichloralphenazone	Dichloralphenazone	86	1773	412	1724	845
Diclofenac	Diclofenac	5006	2079	467	2020	970
Diclofenac Epolamine	Diclofenac Epolamine	52	45	13	43	8
Dicloxacillin	Dicloxacillin	365	47	12	46	17
Dicyclomine	Dicyclomine	17134	26	3	26	5
Dienogest	Dienogest	11	13	5	13	8
Diflorasone Diacetate	Diflorasone	205	2	0	2	1
Diflunisal	Diflunisal	17	2	0	2	1
Difluprednate	Difluprednate	455	6	0	6	4
Digoxin	Digoxin	1064	409	174	401	96
Dihydroergotamine Mesylate	Dihydroergotamine	62	1	0	1	1
Diltiazem	Diltiazem	65	64	10	61	29
Dimethicone	Dimethicone	12	37	2	36	24
Diphencyprone	Diphencyprone	12	49	4	49	19
Diphenhydram	Diphenhydram	1515	243	41	239	87
diphenhydrAMINE	diphenhydrAMINE	2989	9584	2767	9231	3192
Diphenoxylate	Diphenoxylate	1100	20	0	20	4
Diphtheria Toxoid	Diphtheria Toxoid	389	36125	12941	34764	8048
Dipyridamole	Dipyridamole	22	1778	423	1757	472
Disodiu	Disodiu	11	228	75	224	124
Divalproex	Divalproex	13676	3114	1302	3023	1085
DM Hydrobrom	DM Hydrobrom	585419	0	0	0	0
Docosahe	Docosahe	59	883	673	844	410
Docosahexaenoic Aci	Docosahexaenoic Aci	52	845	641	809	400
Docosanol	Docosanol	21	0	0	0	0
Docusate	Docusate	294	148963	50613	144629	62741
Dolutegravir	Dolutegravir	110	97	28	93	59
Donepezil	Donepezil	18	25	3	22	15
Dornase Alfa	Dornase Alfa	3348	130	13	128	80
Dorzolamide	Dorzolamide	2074	27	0	27	10
Doxazosin Mesylate	Doxazosin	640	36	2	35	13
Doxepin	Doxepin	1290	18	4	18	3
Doxycycline	Doxycycline	19231	601	58	592	148
Doxycycline Hyclate	Doxycycline Hyclate	25267	579	55	572	143
Doxylamine	Doxylamine	23	87	26	82	29
Dressing	Dressing	14	12128	2567	11527	1863
Dronabinol	Dronabinol	357	5112	828	4875	835
Drospirenone	Drospirenone	842	31	4	29	14
DSS	DSS	11	764	280	731	175
Dulaglutide	Dulaglutide	16	3	2	1	2
Duloxetine	Duloxetine	2408	38	6	36	15
Dupilumab	Dupilumab	2209	117	16	111	62
Echothiophate Iodide	Echothiophate Iodide	115	5	1	5	3
Econazole Nitrate	Econazole	13172	2	0	2	1
Eculizumab	Eculizumab	17	273	72	273	59
Efavirenz	Efavirenz	44	347	151	338	239
Efinaconazole	Efinaconazole	482	3	1	3	2
Electrolytes	Electrolytes	15	204258	77865	196593	38457
Eletriptan Hydrobromide	Eletriptan	125	2	0	2	1

Elexacaftor	Elexacaftor	400	32	7	29	15
Elosulfase Alfa	Elosulfase Alfa	24	19	10	18	14
Eltrombopag Olamine	Eltrombopag	242	97	20	96	51
Elvitegravir	Elvitegravir	79	32	14	29	21
Emicizumab-kxwh	Emicizumab-kxwh	231	60	22	59	22
Emollient	Emollient	148	12023	6359	11471	2776
Emtricitabine	Emtricitabine	265	537	217	522	306
Enalapril	Enalapril	5850	212	69	203	115
Enoxaparin	Enoxaparin	1006	218	86	216	68
Entecavir	Entecavir	46	80	29	67	46
Epinastine	Epinastine	3199	7	3	7	5
Epinephrine	Epinephrine	386342	3892	1488	3770	1190
Eplerenone	Eplerenone	88	13	3	10	7
Epoetin Alfa	Epoetin Alfa	142	856	587	839	311
Epoetin Alfa-epbx	Epoetin Alfa-epbx	26	0	0	0	0
Ergocalciferol	Ergocalciferol	10698	7441	5600	7234	1718
Erythromycin	Erythromycin	139417	65737	24272	62496	11689
Erythromycin Ethylsuccinate	Erythromycin Ethylsuccinate	4571	9284	3307	8784	2107
Escitalopram Oxalate	Escitalopram Oxalate	32515	81	13	79	28
Eslicarbazepine	Eslicarbazepine	336	458	166	421	108
Esomeprazole	Esomeprazole	12260	206	43	203	98
Estradiol Valerate	Estradiol	11	2185	1661	2065	515
Estradiol	Estradiol	2825	483247	186688	464604	103473
Estradiol V	Estradiol V	11	48	34	48	16
Estrogen	Estrogen	32	483373	186719	464730	103470
Eszopiclone	Eszopiclone	47	1	0	1	1
Etanercept	Etanercept	1682	513	92	508	271
Eteplirsen	Eteplirsen	15	12	1	12	8
Ethambutol	Ethambutol	56	53356	17109	50815	8874
Ethanol	Ethanol	221	74833	28405	72226	16215
Ethinyl	Ethinyl	11	28937	10860	27971	7966
Ethinyl Estradiol	Ethinyl Estradiol	13280	410	177	385	241
Ethosuximide	Ethosuximide	7838	205	63	199	60
Ethyl	Ethyl	94	465751	179380	447578	101021
Ethynodiol Diacetate	Ethynodiol	66	16	4	15	6
Etodolac	Etodolac	323	5	1	5	2
Etonogestrel	Etonogestrel	71	31	4	31	12
Etoposide	Etoposide	67	8086	1752	7856	2502
Everolimus	Everolimus	380	211	70	205	107
Ezetimibe	Ezetimibe	104	86	60	83	36
Factor IX Albumin Fusion Protein	Factor IX Albumin Fusion Protein	42	6	4	6	5
Factor IX Fc Fusion Protein	Factor IX Fc Fusion Protein	39	13	9	13	7
Famciclovir	Famciclovir	286	20	8	20	11
Famotidine	Famotidine	40958	1003	227	975	536
Fe	Fe	1212	461477	178088	443599	101664
Felbamate	Felbamate	773	958	206	903	206
Fenfluramine	Fenfluramine	56	93	39	92	53
Fenofibrate	Fenofibrate	160	30	18	28	13
Fenoprofen	Fenoprofen	17	2	1	2	1
Fentanyl	Fentanyl	68	2308	447	2240	1466
Ferrous	Ferrous	5116	43024	23727	41354	5654
Ferrous Bisglycin	Ferrous Bisglycin	15	8	6	7	8
Ferrous Fum	Ferrous Fum	2311	54	38	51	47

Fesoterodine	Fesoterodine	85	51	33	47	39
Fexofenadine	Fexofenadine	1850	57	6	51	45
Fidaxomicin	Fidaxomicin	44	7	2	7	3
Filgrastim	Filgrastim	449	1196	568	1170	501
Filgrastim-aafi	Filgrastim-aafi	80	0	0	0	0
Filgrastim-sndz	Filgrastim-sndz	74	0	0	0	0
Flavoring Aid	Flavoring Aid	497	217	98	208	106
Flecainide	Flecainide	325	104	26	104	35
Fluconazole	Fluconazole	50845	645	86	636	125
Fludrocortisone	Fludrocortisone	2516	192	94	192	42
Flunisolide	Flunisolide	1365	46	7	43	43
Fluocinolone Acetonide	Fluocinolone Acetonide	67094	97	7	93	71
Fluocinonide	Fluocinonide	24109	20	0	20	13
Fluoride	Fluoride	5898	211575	84349	202713	45731
Fluorometholone	Fluorometholone	3914	32	1	32	16
Fluorouracil	Fluorouracil	4683	681	156	650	144
Fluoxetine	Fluoxetine	79578	748	113	722	317
Fluphenazine	Fluphenazine	124	15	2	15	3
Flurandrenolide	Flurandrenolide	2220	4	0	4	1
Flurbiprofen	Flurbiprofen	57	28	6	27	20
Fluticasone	Fluticasone	835699	771	197	725	642
Fluticasone Furoate	Fluticasone	5388	50	16	38	48
Fluvoxamine	Fluvoxamine	2869	85	14	82	39
Folate Combinat	Folate Combinat	32	7	0	7	4
Foli	Foli	346	3405	1636	3311	826
Folic A	Folic A	114	16103	7682	15602	3740
Folic Ac	Folic Ac	1838	2739	1413	2680	684
Folic Acid	Folic Acid	198057	24496	11718	23702	5844
Fondaparinux	Fondaparinux	19	17	5	17	8
Formaldehyde	Formaldehyde	391	5199	2078	4988	1671
Formoterol	Formoterol	44196	613	256	564	249
Formula, Infant	Formula, Infant	444	1181	502	1091	516
Fosfomycin Tromethamine	Fosfomycin Tromethamine	25	149	26	148	40
Fosphenytoin	Fosphenytoin	15	52	19	51	30
Frovatriptan	Frovatriptan	55	1	1	1	0
Furosemide	Furosemide	4521	651	219	635	178
Gabapentin	Gabapentin	10504	246	40	236	81
Galantamine Hydrobromide	Galantamine	96	10	1	10	3
Ganciclovir	Ganciclovir	532	739	242	723	234
Gatifloxacin	Gatifloxacin	576	57	7	55	25
Gemfibrozil	Gemfibrozil	50	118	26	116	36
Gentamicin	Gentamicin	70932	1496	281	1470	376
GG	GG	695	186182	77822	178057	38191
Glimepiride	Glimepiride	15	5	3	5	2
Glipizide	Glipizide	22	17	13	17	1
Glucagon	Glucagon	23017	1063	780	996	299
Glucose Meter	Glucose Meter	3799	63	48	46	13
Glutamine	Glutamine	69	437072	170240	419499	93722
Glutathione	Glutathione	43	362040	141170	347327	83721
Glyburide	Glyburide	44	75	32	74	11
Glycerin	Glycerin	352	470218	181395	451870	101173
Glycerol Phenylbutyrate	Glycerol Phenylbutyrate	108	46	34	45	16
Glycopyrrolate	Glycopyrrolate	3168	119	18	116	81
Glycopyrronium	Glycopyrronium	274	117	18	114	80

Gramicidin	Gramicidin	1511	70	13	61	30
Granisetron	Granisetron	381	85	15	84	72
Griseofulvin	Griseofulvin	33422	179	8	178	62
Griseofulvin, Ultramicrocrystalline	Griseofulvin, Ultramicrocrystalline	4004	179	8	178	62
Guaifenesin	Guaifenesin	20869	19823	7310	19080	5172
Guanfacine	Guanfacine	173979	116	17	112	70
Halcinonide	Halcinonide	479	14	0	14	13
Halobetasol	Halobetasol	2214	3	0	3	1
Haloperidol	Haloperidol	487	348	49	342	96
HC	HC	180970	26966	13307	25434	4904
HC Ace	HC Ace	1780	0	0	0	0
Heparin	Heparin	216	67275	31338	64775	16411
Histrelin	Histrelin	1101	28	9	28	15
Homatropine Hydrobromide	Homatropine	119	2	0	2	0
Homatropine Methylbromide	Homatropine Methylbromide	2423	2	0	2	2
Hyaluronate	Hyaluronate	24	1368	521	1227	329
Hydralazine	Hydralazine	74	117	20	110	41
Hydrated Silic	Hydrated Silic	15	108	52	102	30
Hydrochlorothiazide	Hydrochlorothiazide	1164	706	255	667	234
Hydrocod Bit	Hydrocod Bit	32	0	0	0	0
Hydrocodone Bitartrate	Hydrocodone	141671	3366	796	3299	1241
Hydrocodone Polistirex	Hydrocodone	2510	0	0	0	0
Hydrocortisone Probutate	Hydrocortisone	34	0	0	0	0
Hydrocortisone Sodium	Hydrocortisone	1983	4882	3393	4633	1015
Hydrocortisone Valerate	Hydrocortisone	19885	1	0	1	1
Hydrocortisone	Hydrocortisone	293120	14795	6359	14408	4360
Hydrocortisone Butyrate	Hydrocortisone Butyrate	8122	4882	3393	4633	1015
Hydromorphone	Hydromorphone	234	97	13	92	44
Hydroquinone	Hydroquinone	50	4435	908	4230	1677
Hydroxocobalam	Hydroxocobalam	92	72	33	72	8
Hydroxocobalamin	Hydroxocobalamin	123	89	46	89	11
Hydroxychloroquine	Hydroxychloroquine	1529	378	60	375	73
Hydroxyurea	Hydroxyurea	1832	44200	24622	42136	9844
Hydroxyzine	Hydroxyzine	140188	168	16	162	107
Hydroxyzine Pamoate	Hydroxyzine	7446	16	1	16	9
Hyoscyamine	Hyoscyamine	22111	54640	23031	52287	10538
Hyoscyamine Sulf	Hyoscyamine Sulf	255	1	0	1	0
Hypochlorous Acid	Hypochlorous Acid	190	167	13	155	60
Hypromellose	Hypromellose	61	63	26	60	18
Ibuprofen	Ibuprofen	149879	60543	23876	57759	10477
Icosapent Ethyl	Icosapent Ethyl	15	1	0	1	0
Idursulfase	Idursulfase	18	47807	20911	46134	12176
Iloperidone	Iloperidone	57	2	1	2	1
Imatinib Mesylate	Imatinib	98	501	63	497	136
Imipramine	Imipramine	4690	480	197	463	155
Imipramine Pamoate	Imipramine	85	0	0	0	0
Imiquimod	Imiquimod	31935	94	1	94	35
Immune Globulin	Immune Globulin	880	408729	168568	391986	91450
Indomethacin	Indomethacin	685	2744	709	2599	563
Infliximab	Infliximab	172	1386	459	1348	567
Infusion Pump, Insulin	Infusion Pump, Insulin	1103	570	345	511	265

Infusion Pump, Parenteral	Infusion Pump, Parenteral	165	0	0	0	0
Insulin Aspart	Insulin Aspart	18391	4281	3148	4048	756
Insulin Aspart Protamine	Insulin Aspart Protamine	34	4279	3147	4046	755
Insulin Degludec	Insulin Degludec	2250	26	17	24	19
Insulin Detemir	Insulin Detemir	2745	46	36	44	36
Insulin Glargine	Insulin Glargine	17277	122	90	117	96
Insulin Glulisine	Insulin Glulisine	233	11	8	10	9
Insulin Human Isophane	Insulin Human Isophane	338	3	2	2	3
Insulin Human Regular	Insulin Human Regular	143	18501	14025	17631	4057
Insulin Lispro	Insulin Lispro	15956	80	57	75	54
Insulin Lispro Protamine	Insulin Lispro Protamine	23	74	52	69	51
Insulin Lispro-aabc	Insulin Lispro-aabc	13	0	0	0	0
Interferon Gamma-1B	Interferon Gamma-1B	47	6	3	6	5
Inulin	Inulin	25	295	208	277	90
Io	Io	19	482042	186178	463427	103271
Iodine	Iodine	26	166501	74169	159132	32025
Iodoquinol	Iodoquinol	285	11	2	11	2
Iohexol	Iohexol	32	223	94	216	54
Ipratropium Bromide	Ipratropium	36537	162	7	162	125
Irbesartan	Irbesartan	109	20	6	16	14
Irinotecan	Irinotecan	26	1938	383	1911	858
Iron Pentacarbonyl	Iron	33	40128	20833	38626	4614
Iron	Iron	544	471035	182030	452796	102530
Iron Polysaccharide	Iron Polysaccharide	21	32970	18016	31752	3199
Isometheptene Mucate	Isometheptene Mucate	106	2384	644	2307	960
Isoniazid	Isoniazid	843	44227	16677	42364	14127
Isopropyl Alcohol	Isopropyl Alcohol	1568	12663	3356	12203	1872
Isotretinoin	Isotretinoin	3043	321	55	317	98
Isradipine	Isradipine	173	7	4	7	4
Itraconazole	Itraconazole	722	898	191	882	216
Ivabradine	Ivabradine	28	21	3	21	8
Ivacaftor	Ivacaftor	2043	167	35	160	83
Ivermectin	Ivermectin	26879	1493	135	1463	582
Ixekizumab	Ixekizumab	40	8	1	8	5
K Cl	K Cl	237	562	152	538	144
K Phos	K Phos	527	21	9	20	8
Ketamine	Ketamine	17	1428	125	1415	857
Ketoconazole	Ketoconazole	124941	331	63	321	97
Ketoprofen	Ketoprofen	22	91	19	90	58
Ketorolac Tromethamine	Ketorolac Tromethamine	4303	228	23	226	154
Ketotifen	Ketotifen	2031	192	40	186	130
L-Methylfolate	L-Methylfolate	385	7	4	6	5
Labetalol	Labetalol	255	204	65	201	72
Lacosamide	Lacosamide	3474	108	34	107	61
Lactic Acid	Lactic Acid	37	3180	2142	3035	404
Lactobacillus Acidophilus	Lactobacillus Acidophilus	103	9917	5721	9511	2202
Lactobacillus Bulgaricus	Lactobacillus Bulgaricus	30	11	3	11	9
Lactobacillus casei	Lactobacillus casei	71	4365	1949	4225	1301
Lactobacillus rhamnosus GG	Lactobacillus rhamnosus GG	174	313	60	303	161
Lactobacillus Sporogenes	Lactobacillus Sporogenes	12	9784	5691	9387	2089
Lactulose	Lactulose	30463	433	259	417	188

Lamivudine	Lamivudine	388	1146	440	1104	746
Lamotrigine	Lamotrigine	18039	705	199	684	281
Lancet	Lancet	14269	48	14	44	16
Lanreotide	Lanreotide	21	21	9	21	9
Lansoprazole	Lansoprazole	29807	114	29	110	75
Latanoprost	Latanoprost	1589	41	2	40	30
Latanoprostene Bunod	Latanoprostene Bunod	11	1	0	1	0
Lecithin	Lecithin	17	5155	2611	4957	1163
Ledipasvir	Ledipasvir	37	60	8	59	46
Leflunomide	Leflunomide	280	133	23	130	35
Letrozole	Letrozole	509	60	20	59	39
Leucovorin	Leucovorin	3055	564	261	556	236
Leuprolide	Leuprolide	3210	239	123	235	138
Levalbuterol	Levalbuterol	58645	30	8	29	27
Levetiracetam	Levetiracetam	35839	738	179	726	383
Levocarnitine	Levocarnitine	6529	1400	1113	1318	241
Levocetirizine Dihydrochloride	Levocetirizine	48968	33	5	31	25
Levodopa	Levodopa	609	783	424	762	151
Levofloxacin	Levofloxacin	5084	372	34	366	81
Levomefolate	Levomefolate	647	29	7	27	16
Levomefolate Ca	Levomefolate Ca	1661	3	3	2	3
Levomilnacipran	Levomilnacipran	13	0	0	0	0
Levonorgestrel	Levonorgestrel	1799	290	32	273	107
Levothyroxine	Levothyroxine	40085	14206	9142	13751	1802
Lidocaine	Lidocaine	59889	1424	172	1341	843
Lifitegrast	Lifitegrast	43	1	0	1	0
Linaclotide	Linaclotide	232	1	1	1	0
Lindane	Lindane	353	237	158	230	22
Linezolid	Linezolid	662	290	50	277	54
Liothyronine	Liothyronine	286	14854	9203	14361	1995
Lipase	Lipase	4554	476304	185736	457750	102051
Liraglutide	Liraglutide	53	36	15	34	15
Lisdexamfetamine Dimesylate	Lisdexamfetamine Dimesylate	183569	109	9	106	68
Lisinopril	Lisinopril	4684	177	73	172	65
Lithium	Lithium	2206	508	202	492	117
Lodoxamide Tromethamine	Lodoxamide Tromethamine	69	5	0	5	5
Lomustine	Lomustine	28	312	59	303	101
Loperamide	Loperamide	641	115	21	108	41
Lopinavir	Lopinavir	145	369	157	366	271
Loratadine	Loratadine	23187	159	20	148	126
Lorazepam	Lorazepam	9277	231	42	221	83
Lorlatinib	Lorlatinib	11	3	1	3	1
Losartan	Losartan	1521	225	78	215	110
Loteprednol Etabonate	Loteprednol	7215	11	0	11	8
Lovastatin	Lovastatin	39	64	36	60	28
Loxapine	Loxapine	42	7	4	7	1
Lubiprostone	Lubiprostone	515	4	0	4	3
Lubricant	Lubricant	9613	7318	2560	7085	1688
Luliconazole	Luliconazole	319	3	0	3	1
Lumacaftor	Lumacaftor	919	62	12	62	29
Lumefantrine	Lumefantrine	28	754	142	737	553
Lurasidone	Lurasidone	1718	20	3	20	10
Macitentan	Macitentan	21	14	6	12	6

Mafenide	Mafenide	12	9	1	9	2
Magnesium	Magnesium	439	178752	78570	171795	50683
Magnesium Hydroxide	Magnesium Hydroxide	373	45901	23092	43749	9116
Magnesium Oxide	Magnesium Oxide	168	30954	14210	29631	8448
Malathion	Malathion	8851	114	37	106	29
Mebendazole	Mebendazole	5158	414	43	412	183
Mecasermin	Mecasermin	49	64	36	62	38
Meclizine	Meclizine	1118	13	1	13	3
Medroxyprogesterone	Medroxyprogesterone	1364	199113	81192	189693	55518
Mefenamic Acid	Mefenamic Acid	127	1806	417	1757	854
Mefloquine	Mefloquine	17726	984	191	962	706
Megestrol	Megestrol	719	98	31	88	30
Melatonin	Melatonin	248	10595	6057	10159	2295
Meloxicam	Meloxicam	6814	23	3	23	10
Memantine	Memantine	775	27	2	24	18
Menthol	Menthol	58	1007	125	964	260
Meperidine	Meperidine	2999	353	37	341	227
Mepolizumab	Mepolizumab	74	46	6	42	24
Mercaptopurine	Mercaptopurine	3637	12360	6556	11894	2843
Meropenem	Meropenem	23	509	109	497	122
Mesalamine	Mesalamine	3158	281	54	275	111
Mesna	Mesna	30	153	31	152	88
Mestranol	Mestranol	15	48	19	45	17
Metaxalone	Metaxalone	379	1	0	1	0
Meter, Peak Flow, Spirometry	Meter, Peak Flow, Spirometry	274	0	0	0	0
Metformin	Metformin	6176	630	273	607	305
Methadone	Methadone	306	663	98	657	187
Methazolamide	Methazolamide	26	1	0	1	0
Methenamine	Methenamine	29	53539	22922	51196	9864
Methenamine Hippurate	Methenamine Hippurate	32	23	11	23	2
Methimazole	Methimazole	1298	260	139	258	49
Methocarbamol	Methocarbamol	892	1629	394	1608	423
Methotrexate	Methotrexate	10084	5725	1435	5675	2326
Methscopolamine Bromide	Methscopolamine	47	2139	1045	2046	492
Methsuximide	Methsuximide	47	12	11	11	6
Methylcellulose	Methylcellulose	39	181	60	167	63
Methylcobalamin	Methylcobalamin	201	1231	921	1197	157
Methylene Blue	Methylene Blue	29	53621	22937	51270	9876
Methylparaben	Methylparaben	14	1603	714	1559	494
Methylphenidate	Methylphenidate	405971	60131	23812	57794	11925
Methylprednisolone	Methylprednisolone	29332	6200	1582	6149	2319
Methylprednisolone Sodium	Methylprednisolone	72	2845	741	2840	851
Metoclopramide	Metoclopramide	3043	2072	470	2015	993
Metolazone	Metolazone	68	2	1	2	2
Metoprolol	Metoprolol	986	252	81	246	102
Metronidazole	Metronidazole	22830	1397	129	1380	493
Mexiletine	Mexiletine	88	49	11	49	10
Mg Hydroxide	Mg Hydroxide	1515	20713	8275	19791	6249
Miconazole Nitrate	Miconazole	1402	2789	475	2754	689
Midazolam	Midazolam	4681	1987	294	1934	1355
Midodrine	Midodrine	347	32	6	28	25
Miglustat	Miglustat	20	81	13	80	27
Milk Protein	Milk Protein	14	1890	1273	1814	668

Mineral Oil	Mineral Oil	56	71	10	68	25
Minocycline	Minocycline	11363	380	40	375	94
Minoxidil	Minoxidil	62	363	90	356	96
Mirabegron	Mirabegron	255	19	3	17	13
Mirtazapine	Mirtazapine	8266	41	3	41	9
Misoprostol	Misoprostol	37	171	9	165	86
Modafinil	Modafinil	233	49	3	49	26
Mometasone Furoate	Mometasone	200518	167	17	158	136
Montelukast	Montelukast	686750	438	115	431	353
Morphine	Morphine	1154	483787	186843	465133	103500
Moxifloxacin	Moxifloxacin	132004	142	19	140	32
Mupirocin	Mupirocin	720916	118	2	112	58
Mycophenolate	Mycophenolate	192	19894	6228	19211	3720
Mycophenolate Mofetil	Mycophenolate Mofetil	2737	1209	416	1201	485
Na Bicarb	Na Bicarb	205	20750	8225	19834	6306
Na Cl	Na Cl	242	398	107	361	87
Na Fluoride	Na Fluoride	25469	1	0	1	0
Na Pho	Na Pho	29	6667	1754	6637	2186
Na Phos	Na Phos	603	6666	1753	6636	2186
Na Phos, Monoba	Na Phos, Monoba	146	0	0	0	0
Na Sulf	Na Sulf	71	0	0	0	0
Nabumetone	Nabumetone	388	6	0	6	4
Nadolol	Nadolol	1602	55	7	48	23
Naftifine	Naftifine	1088	9	1	9	6
Naloxone	Naloxone	307	427	64	420	122
Naltrexone	Naltrexone	513	613	196	595	179
Naphazoline	Naphazoline	45	13	1	13	3
Naproxen	Naproxen	25416	388	156	367	93
Naratriptan	Naratriptan	169	6	1	6	1
Natamycin	Natamycin	19	39	1	39	14
Nebivolol	Nebivolol	29	142	59	138	51
Nebulizer, Direct Patient Interface	Nebulizer, Direct Patient Interface	1382	0	0	0	0
Nedocromil	Nedocromil	91	124	23	116	107
Neomycin	Neomycin	47444	416	35	406	164
Neomycin Sulf	Neomycin Sulf	182720	27	1	26	9
Nepafenac	Nepafenac	45	1	0	1	0
Netarsudil	Netarsudil	58	7	0	7	3
Nevirapine	Nevirapine	42	11806	3443	11445	2492
Niacinamide	Niacinamide	81	14118	6615	13614	3258
Nifedipine	Nifedipine	152	586	276	534	204
Nitazoxanide	Nitazoxanide	2102	63	2	59	40
Nitisinone	Nitisinone	32	58	40	57	15
Nitrofurantoin	Nitrofurantoin	19871	238	9	237	50
Nitrofurantoin Monohydrate	Nitrofurantoin Monohydrate	5081	235	9	234	50
Nitroglycerin	Nitroglycerin	99	470183	181371	451835	101160
Nizatidine	Nizatidine	1854	12	3	12	8
Norelgestromin	Norelgestromin	408	5	3	4	4
Norethindrone	Norethindrone	3882	2219	1668	2097	529
Norethindrone Ace	Norethindrone Ace	2298	21	8	18	9
Norflurane	Norflurane	58	473	43	468	236
Norgestimate	Norgestimate	3927	8	4	7	5
Norgestrel	Norgestrel	717	236	30	219	106
Nortriptyline	Nortriptyline	1920	303	68	289	98
Nusinersen	Nusinersen	68	205	28	205	74

Nystatin	Nystatin	287647	1239	494	1180	287
Octreotide	Octreotide	29	391	106	376	83
Ofloxacin	Ofloxacin	390401	11001	5687	10636	2577
Olanzapine	Olanzapine	3564	296	47	290	118
Olive Oil	Olive Oil	15	157	65	153	104
Olmesartan Medoxomil	Olmesartan Medoxomil	15	177	70	170	55
Olodaterol	Olodaterol	12	1	0	1	0
Olopatadine	Olopatadine	104174	35	1	32	29
Omalizumab	Omalizumab	1064	476	112	453	274
Omega-3 Fatty Acids	Omega-3 Fatty Acids	46	1477	992	1434	660
Omega-3-Acid Ethyl Esters	Omega-3-Acid Ethyl Esters	204	265	162	253	126
Omeprazole	Omeprazole	68064	2176	528	2144	727
OnabotulinumtoxinA	OnabotulinumtoxinA	332	43	3	43	27
Ondansetron	Ondansetron	701313	424	42	421	303
Opium	Opium	21	13644	2891	13214	6318
Orchar	Orchar	40	60	24	54	6
Orphenadrine	Orphenadrine	58	1636	396	1615	423
Oseltamivir	Oseltamivir	832741	685	54	676	197
Ostomy Product	Ostomy Product	46	0	0	0	0
Oxandrolone	Oxandrolone	151	146	61	144	122
Oxaprozin	Oxaprozin	37	0	0	0	0
Oxcarbazepine	Oxcarbazepine	25560	332	127	322	158
Oxiconazole Nitrate	Oxiconazole	1819	2	0	2	2
Oxybutynin	Oxybutynin	20867	192	11	191	117
Oxycodone	Oxycodone	46543	3881	906	3794	1487
Oxymetazoline	Oxymetazoline	80	38072	11661	36692	14276
Oxytocin	Oxytocin	20	817	332	725	294
Ozenoxacin	Ozenoxacin	157	10	0	10	7
p-Phenolsulfonic Acid	p-Phenolsulfonic Acid	41	0	0	0	0
Paliperidone	Paliperidone	665	61	32	60	24
Palivizumab	Palivizumab	2576	371	31	364	183
Pantoprazole	Pantoprazole	6407	124	30	121	62
Paricalcitol	Paricalcitol	26	10	6	10	6
Paromomycin	Paromomycin	65	98	10	96	68
Paroxetine	Paroxetine	2615	146	28	140	47
Pazopanib	Pazopanib	34	23	7	21	8
PEG	PEG	47	4282	1333	4097	1344
PEG Electrolyte Lavage Solution	PEG Electrolyte Lavage Solution	514	0	0	0	0
Pegfilgrastim	Pegfilgrastim	399	36	21	36	27
Pegfilgrastim-bmez	Pegfilgrastim-bmez	46	31	16	31	22
Pegfilgrastim-cbqv	Pegfilgrastim-cbqv	15	31	16	31	22
Pegfilgrastim-jmdb	Pegfilgrastim-jmdb	34	31	16	31	22
Peginterferon Alfa-2B	Peginterferon Alfa-2B	12	22	8	22	17
Penciclovir	Penciclovir	685	2280	338	2248	769
Penicillin G	Penicillin G	27	10475	6810	9973	2042
Penicillin G Benzathine	Penicillin G Benzathine	376	214	26	214	77
Penicillin G Procaine	Penicillin G Procaine	47	56	8	56	24
Penicillin V	Penicillin V	74293	6393	1886	6184	1640
Pentamidine Isethionate	Pentamidine Isethionate	20	148	37	147	50
Pentosan Polysulfate	Pentosan Polysulfate	19	4	0	4	2
Pentoxifylline	Pentoxifylline	42	93	17	89	41
Perampanel	Perampanel	724	101	21	98	69
Permethrin	Permethrin	42897	316	64	291	148
Perphenazine	Perphenazine	54	19	3	19	10

Peru Balsam	Peru Balsam	11	44	0	44	2
Petrolatum	Petrolatum	219	432	48	399	186
Petrolatum, White	Petrolatum, White	1290	168	31	159	71
Phenazopyridine	Phenazopyridine	5031	18	9	18	6
Pheniramine	Pheniramine	21	155	28	152	87
Phenobarb	Phenobarb	226	1260	598	1221	464
Phenobarbital	Phenobarbital	1566	1829	811	1757	586
Phentermine	Phentermine	18	11	1	11	2
Phenyleph	Phenyleph	5891	1292	448	1220	468
Phenylephrine	Phenylephrine	198	18993	7673	17413	3943
Phenytoin	Phenytoin	394	2379	1159	2304	549
Phosphorus	Phosphorus	39	11184	6546	10712	2247
Phytonadione	Phytonadione	453	639	303	619	140
Pilocarpine	Pilocarpine	65	93	35	88	4
Pimecrolimus	Pimecrolimus	25398	106	9	105	65
Pimozide	Pimozide	210	131	26	126	42
Pioglitazone	Pioglitazone	25	168167	66396	162475	33317
Piperonyl Butoxide	Piperonyl Butoxide	14	41	4	38	24
Piroxicam	Piroxicam	162	32	5	32	19
Podofilox	Podofilox	2839	303	86	290	84
Podophyllum	Podophyllum	25	21	4	20	6
Policosanol	Policosanol	18	2	2	2	2
Polyethylene Glycol	Polyethylene Glycol	35	8301	3050	7989	1969
Polyethylene Glycol 3350	Polyethylene Glycol 3350	90501	61	14	61	50
Polymyxin B	Polymyxin B	455260	113	13	111	61
Polymyxin B Sulf	Polymyxin B Sulf	180984	16	0	16	12
Polyureaurethane	Polyureaurethane	94	0	0	0	0
Polyvinyl Alcohol	Polyvinyl Alcohol	34	38	2	37	6
Posaconazole	Posaconazole	153	145	39	143	43
Potassium Nitrate	Potassium	1943	96	8	94	30
Potassium	Potassium	2947	422563	167896	405645	91877
Potassium Bicarbonate	Potassium Bicarbonate	137	15	8	15	5
Potassium Iodide	Potassium Iodide	302	151891	66849	145173	29584
Pramipexole Dihydrochloride	Pramipexole	164	18	4	18	6
Pramoxine	Pramoxine	750	17	2	15	10
Pravastatin	Pravastatin	369	1690	433	1666	465
Praziquantel	Praziquantel	118	1107	178	1094	421
Prazosin	Prazosin	1084	1305	652	1101	200
Prednicarbate	Prednicarbate	519	11	0	11	7
Prednisolone	Prednisolone	511095	9082	2339	9022	3256
Prednisolone Sodium	Prednisolone	673260	6465	1619	6440	2133
Prednisone	Prednisone	267282	5714	1454	5688	2219
Pregabalin	Pregabalin	345	69	8	66	31
Prilocaine	Prilocaine	31815	312	16	286	214
Primaquine	Primaquine	22	278	76	270	140
Primidone	Primidone	80	149	83	142	52
Probenecid	Probenecid	29	75	38	71	25
Procarbazine	Procarbazine	14	726	94	713	213
Prochlorperazine	Prochlorperazine	1502	5486	1491	5319	1137
Progesterone	Progesterone	159	474363	183696	455904	102689
Progestin	Progestin	32	468942	181040	450688	101748
Proguanil	Proguanil	19248	179	41	176	98
Promethazine	Promethazine	93226	1907	424	1858	917
Propafenone	Propafenone	19	60	18	58	26

Propantheline Bromide	Propantheline	17	15	0	15	5
Proparacaine	Proparacaine	263	16	0	16	10
Propranolol	Propranolol	9641	1370	393	1310	525
Propylene Glycol	Propylene Glycol	24	1190	268	1164	525
Propylthiouracil	Propylthiouracil	17	4773	1309	4551	788
Protease	Protease	4546	457321	175037	439453	98724
Protriptyline	Protriptyline	14	3	0	3	1
Prucalopride	Prucalopride	50	9	3	6	8
PSE	PSE	538332	24004	6044	23580	6483
Pseudoephedrine	Pseudoephedrine	1417	48955	20794	46767	11503
Pump, Infusion	Pump, Infusion	106	991	467	912	433
Pyrantel Pamoate	Pyrantel	66	103	8	102	55
Pyrazinamide	Pyrazinamide	14	1352	213	1347	328
Pyridostigmine Bromide	Pyridostigmine	360	190	69	188	43
Pyridoxal Phos	Pyridoxal Phos	32	141	113	134	29
Pyridoxine	Pyridoxine	207	436251	168845	419592	94464
Pyril Mal	Pyril Mal	13	0	0	0	0
Pyrilamine	Pyrilamine	604	42	3	39	25
Quetiapine	Quetiapine	8631	220	37	214	96
Quinidine	Quinidine	91	102	21	96	20
Rabeprazole	Rabeprazole	409	100	19	96	58
Raltegravir	Raltegravir	171	587	254	570	426
Ramelteon	Ramelteon	73	9	0	7	6
Ramipril	Ramipril	11	45	10	42	29
Ranitidine	Ranitidine	105703	215	42	211	105
Reduced Diphtheria Toxoid	Reduced Diphtheria Toxoid	8937	53	10	51	25
Retapamulin	Retapamulin	738	17	0	15	13
Riboflavin	Riboflavin	54	50473	26239	48622	7435
Rifabutin	Rifabutin	40	45	7	44	18
Rifampin	Rifampin	1690	2301	320	2281	530
Rifapentine	Rifapentine	55	32	5	32	18
Rifaximin	Rifaximin	694	88	31	74	44
Rimantadine	Rimantadine	14	29	3	29	15
Risdiplam	Risdiplam	61	23	5	22	9
Risperidone	Risperidone	35990	757	128	739	381
Ritonavir	Ritonavir	209	592	266	577	428
Rivaroxaban	Rivaroxaban	86	59	18	59	21
Rizatriptan	Rizatriptan	14052	7	1	7	5
Romiplostim	Romiplostim	47	60	13	60	33
Ropinirole	Ropinirole	98	19	6	18	5
Rosuvastatin	Rosuvastatin	183	89	61	86	37
Rufinamide	Rufinamide	1262	70	19	69	48
Ruxolitinib	Ruxolitinib	63	84	19	83	34
Saccharin	Saccharin	95	73	22	69	32
Saccharomyces Boulardii	Saccharomyces Boulardii	33	54	3	51	46
Sacrosidase	Sacrosidase	238	5	2	5	2
Sacubitril	Sacubitril	22	7	5	7	4
Salicylic Acid	Salicylic Acid	4696	45744	16671	43940	11122
Saliva Substitutes	Saliva Substitutes	14	18	6	17	4
Salmeterol Xinafoate	Salmeterol Xinafoate	40253	174	27	167	151
Sapropterin Dihydrochloride	Sapropterin	770	74	53	71	36
Sarecycline	Sarecycline	390	1	0	1	1
Sargramostim	Sargramostim	162	514	346	489	160
Sch	Sch	12	266332	105752	257531	68706

Schizochytrium	Schizochytrium	488	8	2	8	7
Scop Hydr	Scop Hydr	226	0	0	0	0
Scopolamine	Scopolamine	3713	140	26	137	69
Selegiline	Selegiline	14	20	4	19	10
Selenium	Selenium	22	11283	6922	10822	2282
Selenium Sulfide	Selenium Sulfide	5912	16	1	16	9
Selexipag	Selexipag	33	14	3	14	2
Selumetinib	Selumetinib	39	40	8	38	21
Sennosides	Sennosides	404	44	3	44	20
Sennosides A and B	Sennosides A and B	18	44	3	44	20
Serdexmethylphenidate	Serdexmethylphenidate	76	4	0	4	4
Sertaconazole Nitrate	Sertaconazole	12	2	0	2	1
Sertraline	Sertraline	101328	280	31	264	116
Sevelamer	Sevelamer	228	27	20	27	12
Sildenafil	Sildenafil	1115	259	51	249	132
Silver Nitrate	Silver	98	93	8	92	28
Silver Sulfadiazine	Silver Sulfadiazine	29076	131	7	120	70
Simethicone	Simethicone	1832	236	61	223	175
Simvastatin	Simvastatin	285	802	309	743	292
Sinecatechins	Sinecatechins	890	4	0	4	2
Sirolimus	Sirolimus	992	795	244	779	283
Sitagliptin	Sitagliptin	22	17	7	15	9
Sodium	Sodium	43845	478076	184802	459657	102157
Sodium Bicarbonate	Sodium Bicarbonate	1655	35534	15758	34116	10209
Sodium Caprate	Sodium Caprate	12	1	1	1	1
Sodium Fluoride	Sodium Fluoride	213460	7438	2484	7198	2026
Sodium Oxybate	Sodium Oxybate	55	67	23	64	27
Sodium Phenylbutyrate	Sodium Phenylbutyrate	25	40	25	38	14
Sodium Picosulfate	Sodium Picosulfate	41	18	0	18	16
Sodium Polystyrene Sulfonate	Sodium Polystyrene Sulfonate	175	9	6	8	2
Sofosbuvir	Sofosbuvir	44	143	26	133	99
Solifenacin	Solifenacin	517	24	2	24	22
Somatropin, E-Coli Derived	Somatropin, E-Coli Derived	20869	221	89	208	178
Somatropin, Mammalian Derived	Somatropin, Mammalian Derived	139	120	48	118	92
Sorafenib	Sorafenib	27	326	53	262	149
Sorbitol	Sorbitol	296	405	176	387	127
Sotalol	Sotalol	243	1020	254	994	168
Soy Protein	Soy Protein	11	157	75	149	81
Spacer, Inhalation	Spacer, Inhalation	148927	413	71	392	318
Spinosad	Spinosad	15620	3	0	3	2
Spironolactone	Spironolactone	2302	886	323	854	245
Squaric Acid Dibutylester	Squaric Acid Dibutylester	70	33	0	33	16
Stannous Fluoride	Stannous Fluoride	63	34	0	34	26
Steviol Glycosides	Steviol Glycosides	14	15	3	14	3
Stiripentol	Stiripentol	43	58	23	57	40
Succimer	Succimer	76	1063	366	1030	146
Sucralfate	Sucralfate	8384	44	2	44	17
Sucrose	Sucrose	1213	34350	18370	33079	3586
Sulconazole Nitrate	Sulconazole	147	1	0	1	1
Sulfacetamide	Sulfacetamide	26355	21	0	21	10
Sulfacetamide Na	Sulfacetamide Na	14	9	0	9	4
Sulfadiazine	Sulfadiazine	25	270	42	256	114

Sulfamethoxazole	Sulfamethoxazole	378987	3731	405	3706	1001
Sulfasalazine	Sulfasalazine	1010	1143	751	1062	225
Sulfur	Sulfur	4047	232799	95996	222416	51977
Sulfuric Acid	Sulfuric Acid	42	4742	2263	4478	1474
Sulindac	Sulindac	114	14	1	14	4
Sumatriptan	Sumatriptan	11808	58	6	56	27
Suvorexant	Suvorexant	30	2	0	2	2
Tacrolimus	Tacrolimus	29509	2162	865	2142	860
Tadalafil	Tadalafil	239	1858	337	1775	276
Tamoxifen	Tamoxifen	42	112	16	107	33
Tamsulosin	Tamsulosin	1733	25	3	25	13
Tavaborole	Tavaborole	129	2	1	2	2
Tazarotene	Tazarotene	3614	42	0	39	29
Teduglutide	Teduglutide	32	14	4	13	10
Temazepam	Temazepam	77	27	5	26	12
Temozolomide	Temozolomide	120	324	47	320	137
Tenofovir Al	Tenofovir Al	78	30	11	30	27
Tenofovir Alafenamide	Tenofovir Alafenamide	96	351	118	340	228
Tenofovir Disoproxil	Tenofovir Disoproxil	109	1294	508	1246	861
Terazosin	Terazosin	517	5	0	5	4
Terbinafine	Terbinafine	8105	156	11	154	65
Terconazole	Terconazole	460	1	0	0	1
Testosterone	Testosterone	25	185124	67769	177202	46983
Testosterone Cypionate	Testosterone Cypionate	283	10	3	10	6
Testosterone Enanthate	Testosterone Enanthate	46	47	23	45	31
Tetanus Toxoid	Tetanus Toxoid	89	122572	57975	116723	23420
Tetrabenazine	Tetrabenazine	80	40	7	40	9
Tetracaine	Tetracaine	761	115	6	114	82
Tetracycline	Tetracycline	277	1175	87	1162	190
Tezacaftor	Tezacaftor	613	40	8	35	20
Thalidomide	Thalidomide	37	231	37	225	57
Theophylline	Theophylline	136	5571	2423	5376	1665
Thiamine	Thiamine	17	59657	28246	57214	8744
Thioctic	Thioctic	24	109	68	98	12
Thioctic Acid	Thioctic Acid	14	54	30	48	10
Thioguanine	Thioguanine	704	7825	4871	7505	1614
Thioridazine	Thioridazine	13	65	9	64	18
Thonzonium Brom	Thonzonium Brom	1736	0	0	0	0
Thonzylamine	Thonzylamine	126	0	0	0	0
Thyroid	Thyroid	939	211483	78376	204142	42803
Tiagabine	Tiagabine	25	43	12	41	23
Timolol	Timolol	5607	246	60	242	104
Tinidazole	Tinidazole	450	1959	173	1918	521
Tiopronin	Tiopronin	18	115	74	107	12
Tiotropium Bromide	Tiotropium	1629	22	7	21	16
Tizanidine	Tizanidine	987	18	1	18	7
Tobramycin	Tobramycin	149931	2118	763	2041	622
Tocilizumab	Tocilizumab	178	253	65	251	93
Tofacitinib	Tofacitinib	97	42	3	41	14
Tolterodine	Tolterodine	1709	44	6	43	36
Topiramate	Topiramate	14319	829	275	804	396
Tramadol	Tramadol	2572	2484	498	2411	1165
Trametinib Dimethyl Sulfoxide	Trametinib Dimethyl Sulfoxide	129	55	2	55	16
Tranexamic Acid	Tranexamic Acid	693	318	72	304	158
Travoprost	Travoprost	119	6	1	6	6

Trazodone	Trazodone	16117	42	5	42	14
Treprostinil	Treprostinil	82	10025	3863	9465	1815
Treprostinil Diolamine	Treprostinil Diolamine	25	57	15	57	34
Tretinoin	Tretinoin	57173	347433	137479	333331	84610
Triamcinolone Acetonide	Triamcinolone Acetonide	602486	539	49	532	208
Triamterene	Triamterene	11	759	270	740	219
Triazolam	Triazolam	1918	16	7	16	7
Trichloroacetic Acid	Trichloroacetic Acid	15	74	27	68	11
Trientine	Trientine	31	11313	4811	10871	3700
Trifarotene	Trifarotene	551	3	0	3	3
Trifluoperazine	Trifluoperazine	15	15	4	14	1
Trifluridine	Trifluridine	297	72	39	70	18
Trihexyphenidyl	Trihexyphenidyl	590	64	5	64	16
Trimethoprim	Trimethoprim	783048	4119	548	4082	1119
Triprolidine	Triprolidine	333	36509	17515	34686	8485
Triptorelin Pamoate	Triptorelin	448	221	103	221	179
Tropicamide	Tropicamide	206	103	3	94	65
Trospium	Trospium	178	4	0	4	4
Tuberculin	Tuberculin	11	2566	672	2534	393
Umeclidinium	Umeclidinium	38	1	0	0	1
Urea	Urea	1117	64632	32070	61592	12507
Uridine Triacetate	Uridine Triacetate	11	1	1	1	0
Ursodiol	Ursodiol	1719	11809	4949	11389	2938
Ustekinumab	Ustekinumab	256	91	17	91	39
Valacyclovir	Valacyclovir	7546	72	22	71	30
Valganciclovir	Valganciclovir	545	149	51	146	67
Valproate	Valproate	16	3423	1381	3331	1172
Valproic Acid	Valproic Acid	4239	3112	1302	3021	1085
Valsartan	Valsartan	42	175	69	168	72
Vancomycin	Vancomycin	1781	1776	417	1736	421
Varenicline	Varenicline	14	12	2	12	2
Vedolizumab	Vedolizumab	30	71	22	71	38
Vemurafenib	Vemurafenib	15	33	4	33	9
Venetoclax	Venetoclax	13	19	1	19	4
Venlafaxine	Venlafaxine	2210	96	18	92	35
Verapamil	Verapamil	453	252	39	245	59
Vigabatrin	Vigabatrin	1014	380	71	375	200
Vilanterol	Vilanterol	16	18	7	10	16
Vilanterol Trifenatate	Vilanterol	1310	1	0	0	1
Vilazodone	Vilazodone	148	8	2	8	4
Viloxazine	Viloxazine	682	20	1	19	16
Vincristine	Vincristine	24	343985	133652	331072	83649
Vita	Vita	1093	83271	36782	79374	16292
Vitami	Vitami	65	15921	10158	15487	3787
Vitamin	Vitamin	547	463233	175804	445410	102669
Vitamin A	Vitamin A	13317	413056	160022	396849	93433
Vitamin B Complex and Vitamin C	Vitamin B Complex and Vitamin C	26	1	1	1	0
Vitamin B1	Vitamin B1	538	46844	24755	45147	6416
Vitamin B12	Vitamin B12	214651	205339	76300	197268	39875
Vitamin B2	Vitamin B2	208364	50468	26236	48618	7435
Vitamin B3	Vitamin B3	208389	4794	1084	4676	1327
Vitamin B5	Vitamin B5	22	102	30	97	46
Vitamin B6	Vitamin B6	208398	51369	26779	49479	7676
Vitamin C	Vitamin C	220698	102299	46831	97697	18215

Vitamin D	Vitamin D	9863	252630	93929	242693	53196
Vitamin E	Vitamin E	61	38215	16497	36898	7390
Von Willebrand Factor	Von Willebrand Factor	176	2610	1434	2536	602
Voriconazole	Voriconazole	565	477	145	466	116
Vorinostat	Vorinostat	17	3009	628	2896	792
Vortioxetine Hydrobromide	Vortioxetine	110	6	2	5	5
Warfarin	Warfarin	906	673	128	665	150
Water	Water	795	119880	36399	114335	29817
Water, Sterile	Water, Sterile	1488	10284	3941	9699	1813
Wound Care Preparation	Wound Care Preparation	18	0	0	0	0
Xylitol	Xylitol	13	1173	397	1099	479
Zafirlukast	Zafirlukast	291	26	2	24	22
Zaleplon	Zaleplon	39	5	0	5	0
Zanamivir	Zanamivir	112	235	35	232	70
Zidovudine	Zidovudine	201	8327	2434	8125	3303
Zinc	Zinc	158	4610	3101	4427	1188
Zinc Oxide	Zinc Oxide	1483	72575	24225	69140	13244
Ziprasidone	Ziprasidone	2199	66	10	66	30
Zolmitriptan	Zolmitriptan	1291	17	2	15	14
Zolpidem	Zolpidem	232	2040	813	2013	77
Zonisamide	Zonisamide	4776	162	50	158	67

Pediatric (12-18 Years Old) Drug Publication Frequency

Origianl Drug Name	Cleaned Drug Name	peds: 12~18 frequency	All publication	PK publication	PE publication	CT publication
1,1,1,3,3-Pentafluoropropane	1,1,1,3,3-Pentafluoropropane	48	320	27	287	187
Abacavir	Abacavir	130	713	280	656	223
Abatacept	Abatacept	212	79	13	78	40
AbobotulinumtoxinA	AbobotulinumtoxinA	14	15	2	14	12
Acamprosate	Acamprosate	47	6	1	5	3
Acarbose	Acarbose	37	11	6	7	5
Acebutolol	Acebutolol	20	40	24	33	6
Acetaminophen	Acetaminophen	615581	46737	13087	43638	10356
Acetazolamide	Acetazolamide	5628	193	26	191	25
Acetic Acid	Acetic Acid	2808	37589	17746	34348	8373
Acetic Acid Glacial	Acetic Acid Glacial	13	1049	452	965	114
Acetone	Acetone	11	515	130	456	247
Acetylcysteine	Acetylcysteine	179	8620	3725	7863	2260
Acitretin	Acitretin	61	103	12	101	16
Acrivastine	Acrivastine	101	6	0	3	6
Acyclovir	Acyclovir	29504	534	74	513	180
Adalimumab	Adalimumab	7581	378	59	369	223
Adapalene	Adapalene	294910	684	260	628	178
Agalsidase Beta	Agalsidase Beta	26	14	8	14	5
AHF	AHF	150	1438	705	1364	436
AHF VIII Sucrose Formulated	AHF VIII Sucrose Formulated	79	0	0	0	0
Al Hydroxide	Al Hydroxide	1638	123	43	109	88
Albendazole	Albendazole	1629	824	199	762	327
Albumin-Free	Albumin-Free	284	26	17	26	22
Albuterol	Albuterol	707274	1292	115	941	987
Alcaftadine	Alcaftadine	293	1	0	1	1
Alclometasone Dipropionate	Alclometasone	6007	0	0	0	0

Alendronate	Alendronate	283	60	21	59	40
Alfuzosin	Alfuzosin	45	3	1	3	2
Allopurinol	Allopurinol	589	224	73	219	85
Almond Oil	Almond Oil	19	3	0	2	2
Almotriptan	Almotriptan	844	6	3	6	4
Aloe	Aloe	154	53145	19690	48687	13875
Aloe Vera	Aloe Vera	16	11793	3643	11010	1539
Alpha Carotene	Alpha Carotene	14	6080	3805	5543	1204
Alprazolam	Alprazolam	20496	45	15	32	7
Alteplase	Alteplase	18	21021	7759	19725	4784
Aluminum	Aluminum	22138	293576	116354	271674	61290
Aluminum Hydroxide	Aluminum Hydroxide	163	27383	14277	24753	4948
Amantadine	Amantadine	4118	95	6	92	42
Ambrisentan	Ambrisentan	97	7	2	5	6
Amcinonide	Amcinonide	20	0	0	0	0
Amikacin	Amikacin	29	407	94	382	154
Amiloride	Amiloride	244	531	169	486	147
Amino Acids	Amino Acids	231	293722	116399	271810	61301
Aminocaproic Acid	Aminocaproic Acid	712	96	27	92	33
Aminolevulinic Acid	Aminolevulinic Acid	40	35570	16247	32504	9101
Amiodarone	Amiodarone	50	158	35	150	48
Amitriptyline	Amitriptyline	42531	159	32	139	56
Amlodipine	Amlodipine	17	70	32	65	36
Amlodipine Besylate	Amlodipine Besylate	5563	70	32	65	36
Ammonium	Ammonium	7188	21488	10280	19648	4384
Amoxicillin	Amoxicillin	1487929	30016	11111	27514	9510
Amphetamine	Amphetamine	10034	156053	52775	142567	42618
Amphotericin B	Amphotericin B	17	62925	26902	57656	13954
Ampicillin	Ampicillin	8332	222839	77194	206916	56255
Amylase	Amylase	2602	712	469	647	109
Anakinra	Anakinra	127	248	148	235	61
Anastrozole	Anastrozole	3937	29	10	26	19
Anthralin	Anthralin	111	26	1	26	13
Antibacterial	Antibacterial	375	25649	6246	24145	5486
Antihemophilic Factor Plasma	Antihemophilic Factor Plasma	284	2545	1007	2400	643
Antihemophilic Factor VIII	Antihemophilic Factor VIII	51	1440	706	1366	438
Antihemophilic Factor VIII Fc Fusion Protein Recom	Antihemophilic Factor VIII Fc Fusion Protein Recom	86	0	0	0	0
Antihemophilic Factor VIII Pegylated	Antihemophilic Factor VIII Pegylated	57	0	0	0	0
Antipyrine	Antipyrine	325	3968	1714	3564	1093
APAP	APAP	1087	41456	12017	38783	8749
Apixaban	Apixaban	499	15	5	13	7
Apraclonidine	Apraclonidine	42	6	1	6	4
Apremilast	Apremilast	198	2	1	2	1
Aprepitant	Aprepitant	497	118	30	110	32
Arformoterol	Arformoterol	13	0	0	0	0
Arginine	Arginine	38	293722	116399	271810	61301
Aripiprazole	Aripiprazole	70567	280	42	270	133
Aripiprazole Lauroxil	Aripiprazole Lauroxil	35	1	0	1	0
Armodafinil	Armodafinil	500	48	2	47	24
Artemether	Artemether	53	326	72	309	221
Ascorbic Acid	Ascorbic Acid	166	27126	14825	24722	3793

Ascorbyl	Ascorbyl	153	930	544	863	199
Asenapine	Asenapine	1285	7	2	7	5
Asfotase Alfa	Asfotase Alfa	21	12	7	9	7
Aspirin	Aspirin	6243	30583	13105	27730	8170
Atazanavir	Atazanavir	15	370	92	351	117
Atenolol	Atenolol	6394	74	17	68	41
Atomoxetine	Atomoxetine	49641	393	30	376	244
Atorvastatin	Atorvastatin	3103	872	211	831	285
Atovaquone	Atovaquone	17239	65	11	63	28
Atropine	Atropine	5341	534	59	512	256
Atropine Sulf	Atropine Sulf	315	25	0	24	17
Avobenzone	Avobenzone	93	1	0	1	0
Azathioprine	Azathioprine	2516	2460	1069	2326	584
Azelaic Acid	Azelaic Acid	14884	16	2	12	13
Azelastine	Azelastine	55003	72	3	40	64
Azithromycin	Azithromycin	738835	617	75	567	299
Aztreonam	Aztreonam	315	102	24	89	51
Bacitracin	Bacitracin	3314	48	0	48	14
Bacitracin Zn	Bacitracin Zn	217	0	0	0	0
Baclofen	Baclofen	8591	237	12	229	94
Baloxavir Marboxil	Baloxavir	5512	17	2	16	13
Balsalazide Disodium	Balsalazide	694	3	1	3	3
Barium	Barium	15	145817	53244	134356	33359
Barrier Skin Protectant	Barrier Skin Protectant	1374	32	1	29	7
Beclomethasone Dipropionate	Beclomethasone	63424	322	51	199	300
Belimumab	Belimumab	31	10	2	10	2
Benazepril	Benazepril	194	6	0	5	6
Benralizumab	Benralizumab	123	18	2	12	14
Benzalkonium	Benzalkonium	14	180	16	157	87
Benzocaine	Benzocaine	353	4306	1749	3888	1177
Benzonatate	Benzonatate	152769	5	2	5	0
Benzoyl Peroxide	Benzoyl Peroxide	402865	200	13	176	99
Benztropine Mesylate	Benztropine	4745	13	0	13	4
Benzyl Alcohol	Benzyl Alcohol	305	3348	1197	2999	1219
Bepotastine Besilate	Bepotastine	1152	7	0	5	7
Besifloxacin	Besifloxacin	924	10	0	9	10
Beta Carotene	Beta Carotene	14	39413	19942	35838	8251
Betaine	Betaine	193	817	540	743	187
Betaine Anhydrous	Betaine Anhydrous	37	113	80	100	27
Betamethasone Dipropionate	Betamethasone	43859	24	1	23	19
Betamethasone Valerate	Betamethasone	6818	32	0	28	24
Betaxolol	Betaxolol	90	13	7	13	6
Bethanechol	Bethanechol	476	10	0	10	1
Bi Subcitrate K	Bi Subcitrate K	85	0	0	0	0
Bicalutamide	Bicalutamide	95	2	0	2	0
Bicarbonates	Bicarbonates	27	20536	9308	18621	6080
Bictegravir	Bictegravir	110	6	3	5	4
Bif	Bif	244	558	165	511	175
Bifidobacterium breve	Bifidobacterium breve	246	11	3	10	10
Bifidobacterium infantis	Bifidobacterium infantis	254	11256	3457	10733	1812
Bifidobacterium lactis	Bifidobacterium lactis	164	32	3	28	32
Bimatoprost	Bimatoprost	233	7	0	6	6
Bioflavonoid	Bioflavonoid	169	948	551	879	207
Biotin	Biotin	1154	1891	1330	1688	388

Bisacodyl	Bisacodyl	550	8428	3326	7801	1736
Bismuth Tribromophenate	Bismuth	20	0	0	0	0
Bisoprolol	Bisoprolol	193	14	1	13	7
Bleomycin	Bleomycin	11	978	81	961	218
Boron	Boron	38	227	89	213	70
Bosentan	Bosentan	41	67	17	63	44
Brexpiprazole	Brexpiprazole	1269	1	1	0	0
Brimonidine	Brimonidine	1498	12	1	12	7
Brinzolamide	Brinzolamide	227	4	0	4	2
Brivaracetam	Brivaracetam	501	25	7	20	19
Bromfenac	Bromfenac	238	3	0	3	2
Bromocriptine Mesylate	Bromocriptine	137	278	148	230	50
Brompheniramine Mal	Brompheniramine Mal	160499	5	0	4	5
Budesonide	Budesonide	56130	667	124	480	560
Bumetanide	Bumetanide	80	20	6	18	9
Buprenorphine	Buprenorphine	240	148	15	125	46
Bupropion	Bupropion	79839	108	15	93	33
buPROPion hydrobromide	buPROPion	43	1	1	1	0
Burosumab-twza	Burosumab-twza	47	14	8	14	10
Buspirone	Buspirone	44260	22	2	21	9
Butalbital	Butalbital	13842	38750	11118	36433	7831
Butenafine	Butenafine	44	1	0	1	1
Butoconazole Nitrate	Butoconazole	98	0	0	0	0
Butorphanol	Butorphanol	23	19	2	18	14
Butylated Hydroxytoluene	Butylated Hydroxytoluene	22	24	13	22	6
C1 Esterase Inhibitor	C1 Esterase Inhibitor	66	216	134	192	49
C30-45 Alkyl Cetearyl Dim	C30-45 Alkyl Cetearyl Dim	18	0	0	0	0
Ca	Ca	2661	292116	115586	270303	61033
Ca As	Ca As	80	122	58	116	21
Ca Ascorbate	Ca Ascorbate	319	1	0	1	1
Ca Cl	Ca Cl	44	44	12	40	9
Ca Oxybate	Ca Oxybate	21	0	0	0	0
Ca Pantothenate	Ca Pantothenate	544	0	0	0	0
Cabergoline	Cabergoline	588	84	42	70	8
Cabozantinib	Cabozantinib	17	7	3	7	1
Caff	Caff	316	2256	538	2109	643
Caffeine	Caffeine	13354	61941	20839	57940	12170
Calcipotriene	Calcipotriene	3641	44	2	44	27
Calcitonin	Calcitonin	71	3496	2870	2964	374
Calcitriol	Calcitriol	1751	778	484	727	230
Calcium	Calcium	704	293051	116080	271216	61242
Canagliflozin	Canagliflozin	52	3	2	2	2
Canakinumab	Canakinumab	148	103	21	103	55
Candesartan Cilexetil	Candesartan Cilexetil	143	9	4	9	6
Candida Albicans Antigen	Candida Albicans Antigen	22	449	117	418	73
Cannabidiol	Cannabidiol	827	177	63	169	87
Capsaicin	Capsaicin	32	153	89	141	12
Captopril	Captopril	45	262447	102975	244615	55374
Carbamazepine	Carbamazepine	2962	1338	685	1226	512
Carbamide Peroxide	Carbamide Peroxide	75	21	1	19	6
Carbidopa	Carbidopa	489	63	25	60	21

Carbinoxamine	Carbinoxamine	1983	2	1	2	1
Carboxymethylcellulose	Carboxymethylcellulose	108	43	17	34	27
Cariprazine	Cariprazine	1276	2	1	2	0
Carisoprodol	Carisoprodol	685	8358	3838	7751	1571
Carvedilol	Carvedilol	793	332	79	317	49
Cefaclor	Cefaclor	227	3018	1162	2815	724
Cefadroxil	Cefadroxil	17644	68	24	66	33
Cefazolin	Cefazolin	16	271	65	251	101
Cefdinir	Cefdinir	259574	53	26	49	28
Cefepime	Cefepime	21	113	22	101	49
Cefixime	Cefixime	3058	112	22	111	61
Cefpodoxime Proxetil	Cefpodoxime Proxetil	1310	83	40	78	58
Cefprozil	Cefprozil	18936	26	7	26	16
Ceftazidime	Ceftazidime	18	470	118	450	184
Ceftibuten	Ceftibuten	34	17	4	16	12
Ceftriaxone	Ceftriaxone	495	798	91	766	253
Cefuroxime Axetil	Cefuroxime Axetil	36960	237	24	228	63
Celecoxib	Celecoxib	6105	48	7	44	26
Cellulose	Cellulose	118	1648	445	1424	632
Cenobamate	Cenobamate	76	2	2	1	1
Cephalexin	Cephalexin	331949	164	30	158	82
Certolizumab Pegol	Certolizumab Pegol	65	26	4	23	12
Cetirizine	Cetirizine	26660	118	9	87	87
Cetrorelix	Cetrorelix	17	8	3	6	6
Cevimeline	Cevimeline	13	0	0	0	0
Chlophedianol	Chlophedianol	470	0	0	0	0
Chlorcyclizine	Chlorcyclizine	148	8	0	7	4
Chlordiazepoxide	Chlordiazepoxide	458	872	393	757	259
Chlorhexidine	Chlorhexidine	174616	302	6	272	208
Chloride	Chloride	68	239996	95025	221567	52125
Chloroquine	Chloroquine	1126	1119	213	1081	414
Chlorothiazide	Chlorothiazide	135	81	36	72	33
Chloroxylenol	Chloroxylenol	11	3	1	2	0
Chlorpheniramine	Chlorpheniramine	30	11537	5762	10235	1453
Chlorpheniramine Polistirex	Chlorpheniramine	4541	0	0	0	0
Chlorpheniramine Mal	Chlorpheniramine Mal	623	18	0	13	13
Chlorpromazine	Chlorpromazine	1260	182	64	163	47
Chlorthalidone	Chlorthalidone	280	82	18	76	45
Chlorzoxazone	Chlorzoxazone	689	887	204	805	344
Cho	Cho	14	208872	81692	195919	48658
Cholecal	Cholecal	14	640	549	539	298
Cholecalc	Cholecalc	92	640	549	539	298
Cholecalcif	Cholecalcif	27	638	547	538	298
Cholecalcife	Cholecalcife	16	638	547	538	298
Cholecalciferol	Cholecalciferol	5769	135844	49002	126377	27495
Cholestyramine	Cholestyramine	910	66	42	63	20
Choline	Choline	53	138500	50800	127150	36040
Chorionic Gonadotropin	Chorionic Gonadotropin	33	2222	1381	2042	324
Ciclesonide	Ciclesonide	2635	50	9	26	48
Ciclopirox	Ciclopirox	12382	15	0	13	8
Ciclopirox Olamine	Ciclopirox	3978	15	0	13	8
Cidofovir	Cidofovir	12	136	21	129	52
Cilostazol	Cilostazol	16	3	1	3	1
Cimetidine	Cimetidine	4417	132	45	128	58
Cinacalcet	Cinacalcet	18	43	23	41	11

Ciprofloxacin	Ciprofloxacin	148129	1152	248	1083	251
Citalopram Hydrobromide	Citalopram	48989	186	35	165	70
Citric Acid	Citric Acid	938	11564	4903	10255	3379
Clarithromycin	Clarithromycin	26749	35103	13439	32808	5211
Clascoterone	Clascoterone	319	5	1	4	5
Clavulanate	Clavulanate	487765	1291	127	1250	505
Clemastine	Clemastine	128	896	201	811	354
Clidinium Bromide	Clidinium	324	8	4	6	2
Clindamycin	Clindamycin	681012	649	42	579	257
Clindamycin Palmitate	Clindamycin	4015	648	42	578	257
Clioquinol	Clioquinol	132	18	0	18	5
Clobazam	Clobazam	3795	38936	12671	36181	8414
Clobetasol	Clobetasol	44570	66	3	62	43
Clocortolone Pivalate	Clocortolone	1855	3	0	2	3
Clomiphene	Clomiphene	56	209	80	176	48
Clomipramine	Clomipramine	1680	86	25	75	29
Clonazepam	Clonazepam	23149	216	68	206	61
Clonidine	Clonidine	70669	470	237	453	217
Clopidogrel Hydrogen	Clopidogrel Hydrogen	285	44	8	42	17
Clorazepate Dipotassium	Clorazepate	505	9	3	9	3
Clotrimazole	Clotrimazole	34680	88	1	63	63
Clozapine	Clozapine	399	196	50	171	37
Coagulation Factor IX	Coagulation Factor IX	65	48171	17508	44494	12797
Coagulation Factor VIIa	Coagulation Factor VIIa	36	278	85	270	119
Coal Tar	Coal Tar	14	90072	22946	84583	35700
Cobamamide	Cobamamide	342	743	606	621	84
Cobicistat	Cobicistat	131	14	9	7	7
Codeine	Codeine	138917	2138	515	1990	644
Codeine Polistirex	Codeine	42	0	0	0	0
Codeine Phos	Codeine Phos	2221	9	2	8	4
Coenzyme Q10	Coenzyme Q10	99	188	130	175	59
Colchicine	Colchicine	944	300	61	289	65
Colesevelam	Colesevelam	269	4	1	2	3
Colestipol	Colestipol	267	16	14	16	15
Colistimethate	Colistimethate	88	17	4	16	10
Colistin Sulf	Colistin Sulf	883	1	0	1	0
Collagenase	Collagenase	267	40334	15574	37119	8685
Collodion	Collodion	25	7	5	7	2
Colloidal Sulfur	Colloidal Sulfur	2581	108	14	108	4
Condom	Condom	11	1409	20	1377	68
Conjugated Estrogens	Conjugated Estrogens	1047	2566	1556	2047	536
Copper	Copper	207	202426	77652	187584	41351
Cosyntropin	Cosyntropin	20	159	109	136	58
Cr	Cr	603	220158	92558	202935	45858
Crisaborole	Crisaborole	11423	15	2	15	14
Crizotinib	Crizotinib	12	35	8	34	11
Cromolyn	Cromolyn	2566	433	74	368	320
Crotamiton	Crotamiton	29	6	0	6	3
Cu	Cu	4179	199560	75923	184945	40645
Cu Sulf	Cu Sulf	45	0	0	0	0
Curcumin	Curcumin	346	20	3	17	11
Cyanocobalamin	Cyanocobalamin	1473	27427	15054	25140	3985
Cyclobenzaprine	Cyclobenzaprine	56495	7	2	6	1
Cyclopentolate	Cyclopentolate	1499	67	1	64	40
Cyclophosphamide	Cyclophosphamide	51	224329	89281	209793	50208

Cyclosporine	Cyclosporine	1444	4670	1824	4456	1410
Cyproheptadine	Cyproheptadine	38509	71	9	57	44
Cysteamine	Cysteamine	43	116586	60203	105298	28591
Cysteamine Bitartrate	Cysteamine	66	75	33	72	24
Cytarabine	Cytarabine	33	1485	243	1449	741
Dabigatran Etexilate Mesylate	Dabigatran Etexilate	14	20	8	18	9
Dabrafenib Mesylate	Dabrafenib	19	9	2	9	6
Danazol	Danazol	26	115	38	103	41
Dantrolene	Dantrolene	125	55	9	55	15
Dapagliflozin Propanediol	Dapagliflozin Propanediol	63	10	8	9	5
Dapsone	Dapsone	69540	15371	3898	14678	2297
Darbepoetin Alfa	Darbepoetin Alfa	80	44	28	43	22
Darifenacin Hydrobromide	Darifenacin	39	0	0	0	0
Darunavir	Darunavir	13	52	29	41	32
Darunavir Ethanolate	Darunavir Ethanolate	16	52	29	41	32
Dasatinib	Dasatinib	84	48	10	48	20
Deferasirox	Deferasirox	265	171	126	170	115
Deferiprone	Deferiprone	35	156	104	155	85
Deflazacort	Deflazacort	238	56	12	56	34
Denosumab	Denosumab	13	54	12	53	10
Dermatophagoides Farinae Ext	Dermatophagoides Farinae Ext	39	7	0	6	5
Dermatophagoides Pter	Dermatophagoides Pter	39	442	229	390	145
Desipramine	Desipramine	184	86518	26598	79565	36126
Desloratadine	Desloratadine	5210	41	2	24	35
Desmopressin	Desmopressin	19517	471	147	462	236
Desogestrel	Desogestrel	47330	471	82	365	285
Desonide	Desonide	22615	667	124	480	567
Desoximetasone	Desoximetasone	6700	8	0	8	5
Desvenlafaxine	Desvenlafaxine	10682	30	13	24	9
Deutetrabenazine	Deutetrabenazine	12	5	0	5	5
Dexamethasone	Dexamethasone	166809	261944	102669	244179	55254
Dexamethasone Sodium	Dexamethasone	1784	19	4	18	15
Dexbrompheniramine	Dexbrompheniramine	301	10	0	10	1
Dexchlorpheniramine	Dexchlorpheniramine	16	8	2	6	5
Dexchlorpheniramine Mal	Dexchlorpheniramine Mal	442	3	0	2	3
Dexlansoprazole	Dexlansoprazole	882	4	1	4	3
Dexmethylphenidate	Dexmethylphenidate	106274	53	7	48	34
Dextroamphetamine	Dextroamphetamine	8146	347	29	324	135
Dextromethorphan Hydrobromide	Dextromethorphan	367	1767	547	1548	682
Dextromethorphan Polistirex	Dextromethorphan	28	65	27	50	19
Dextrose	Dextrose	231	18429	10809	16440	4492
Diazepam	Diazepam	45873	62749	28171	57871	8656
Diazoxide	Diazoxide	29	57	26	54	20
Dichloralphenazone	Dichloralphenazone	657	880	200	799	341
Diclofenac	Diclofenac	39437	1087	233	981	427
Diclofenac Epolamine	Diclofenac Epolamine	339	25	8	24	7
Dicloxacillin	Dicloxacillin	1283	22	6	22	7
Dicyclomine	Dicyclomine	36920	9	1	9	2
Dienogest	Dienogest	816	79	13	45	68

Diethylpropion	Diethylpropion	50	1849	671	1681	638
Diflorasone Diacetate	Diflorasone	320	2	0	2	1
Diflunisal	Diflunisal	979	4	0	3	2
Difluprednate	Difluprednate	879	2	0	2	2
Digoxin	Digoxin	682	197	90	192	54
Dihydrocodeine Bitartrate	Dihydrocodeine	28	1287	329	1183	396
Dihydroergotamine Mesylate	Dihydroergotamine	457	1	0	1	1
Diltiazem	Diltiazem	357	49	14	41	21
Dimethicone	Dimethicone	17	16	1	16	14
Dimethyl	Dimethyl	66	293722	116399	271810	61301
Dimethyl Sulfoxide	Dimethyl Sulfoxide	14	113	15	111	26
Diphenhydram	Diphenhydram	1638	146	17	142	58
diphenhydrAMINE	diphenhydrAMINE	946	5186	1520	4719	1681
Diphenoxylate	Diphenoxylate	3938	12	1	12	7
Diphtheria Toxoid	Diphtheria Toxoid	77	20503	6976	19229	3926
Dipyridamole	Dipyridamole	43	911	201	876	290
Disodiu	Disodiu	58	130	31	115	82
Disopyramide	Disopyramide	17	30	5	29	8
Disulfiram	Disulfiram	52	13	4	11	4
Divalproex	Divalproex	21066	1926	928	1786	755
DM Hydrobrom	DM Hydrobrom	202515	0	0	0	0
Docosahe	Docosahe	149	410	322	368	146
Docosahexa	Docosahexa	87	409	321	367	146
Docosahexaenoic A	Docosahexaenoic A	282	389	302	350	140
Docosahexaenoic Aci	Docosahexaenoic Aci	193	385	300	346	140
Docosahexaenoic Acid	Docosahexaenoic Acid	310	1602	816	1475	383
Docosanol	Docosanol	22	0	0	0	0
Docusate	Docusate	927	94831	31982	87318	39610
Dolutegravir	Dolutegravir	287	76	20	70	41
Donepezil	Donepezil	71	18	1	16	11
Dornase Alfa	Dornase Alfa	1832	97	9	93	61
Dorzolamide	Dorzolamide	1409	11	0	11	5
Doxazosin Mesylate	Doxazosin	370	23	3	22	10
Doxepin	Doxepin	3170	16	4	15	3
Doxycycline	Doxycycline	91920	488	52	444	158
Doxycycline Hyclate	Doxycycline Hyclate	272837	475	49	431	157
Doxylamine	Doxylamine	578	48	21	42	17
Dronabinol	Dronabinol	635	3137	551	2936	476
Drospirenone	Drospirenone	73745	164	15	91	126
Droxidopa	Droxidopa	15	31	16	30	1
DSS	DSS	443	532	194	488	107
Dulaglutide	Dulaglutide	228	3	1	3	1
Duloxetine	Duloxetine	23923	32	5	29	16
Dupilumab	Dupilumab	4784	65	7	55	39
Echothiophate Iodide	Echothiophate Iodide	21	1	0	1	1
Econazole Nitrate	Econazole	10297	3	0	2	2
Eculizumab	Eculizumab	16	120	33	116	38
Efavirenz	Efavirenz	40	230	109	205	145
Efinaconazole	Efinaconazole	1074	3	1	3	2
Eflornithine	Eflornithine	33	30	8	27	15
Eicosap	Eicosap	49	250	196	219	99
Eicosapentaenoic Acid	Eicosapentaenoic Acid	122	40174	15422	36956	8794
Elagolix	Elagolix	166	1	0	1	1

Eletriptan Hydrobromide	Eletriptan	1864	1	0	1	1
Elexacaftor	Elexacaftor	626	21	2	18	6
Eliglustat	Eliglustat	12	3	1	2	2
Eltrombopag Olamine	Eltrombopag	236	52	12	52	29
Eluxadoline	Eluxadoline	139	0	0	0	0
Elvitegravir	Elvitegravir	115	22	11	15	12
Emicizumab-kxwh	Emicizumab-kxwh	111	26	11	24	14
Emollient	Emollient	170	7234	3849	6584	1740
Empagliflozin	Empagliflozin	57	7	3	6	5
Emtricitabine	Emtricitabine	1039	394	175	336	195
Enalapril	Enalapril	3392	129	47	123	80
Enoxaparin	Enoxaparin	2272	129	50	126	54
Entecavir	Entecavir	48	38	19	35	19
Epinastine	Epinastine	1628	4	0	4	3
Epinephrine	Epinephrine	159915	2321	995	2127	657
Eplerenone	Eplerenone	155	8	2	7	5
Epoetin Alfa	Epoetin Alfa	125	583	431	527	206
Epoetin Alfa-epbx	Epoetin Alfa-epbx	14	0	0	0	0
Erenumab-aooe	Erenumab-aooe	431	3	1	2	2
Ergocalciferol	Ergocalciferol	40625	4647	3715	4023	967
Ergotamine	Ergotamine	62	458	132	431	67
Ertapenem	Ertapenem	14	34	5	30	11
Erythromycin	Erythromycin	70048	41203	15738	38378	6518
Erythromycin Ethylsuccinate	Erythromycin Ethylsuccinate	1281	5455	2008	4997	1038
Escitalopram Oxalate	Escitalopram Oxalate	173013	79	18	68	29
Eslicarbazepine	Eslicarbazepine	305	332	115	288	90
Esomeprazole	Esomeprazole	11908	153	28	137	84
Estetrol	Estetrol	31	161	18	94	121
Estradiol	Estradiol	6974	293551	116339	271644	61299
Estradiol Valerate	Estradiol	874	3583	2097	2665	1312
Estradiol Cypionate	Estradiol Cypionate	16	16	3	12	13
Estradiol V	Estradiol V	816	86	35	57	57
Estrogen	Estrogen	4393	293600	116354	271692	61298
Eszopiclone	Eszopiclone	840	2	0	1	2
Etanercept	Etanercept	2276	350	63	342	211
Ethambutol	Ethambutol	45	32566	10680	30369	5138
Ethanol	Ethanol	65	49000	18635	45788	10508
Ethinyl	Ethinyl	1126	20389	7279	18662	5996
Ethinyl Estrad	Ethinyl Estrad	1588	1212	325	898	835
Ethinyl Estradiol	Ethinyl Estradiol	809499	1437	404	1077	944
Ethosuximide	Ethosuximide	2852	119	41	117	43
Ethyl	Ethyl	84	286263	112840	264784	60301
Ethynodiol Diacetate	Ethynodiol	4025	35	13	33	22
Etodolac	Etodolac	7106	6	1	5	3
Etonogestrel	Etonogestrel	17171	158	30	136	83
Etoposide	Etoposide	36	5247	1130	4983	1712
Everolimus	Everolimus	256	121	53	117	72
Exenatide	Exenatide	48	23	7	19	18
Ezetimibe	Ezetimibe	361	62	36	57	31
Factor IX Albumin Fusion Protein	Factor IX Albumin Fusion Protein	31	4	2	4	3
Factor IX Fc Fusion Protein	Factor IX Fc Fusion Protein	34	9	6	9	6
Famciclovir	Famciclovir	1434	19	5	17	11

Famotidine	Famotidine	29896	516	117	462	286
Fe	Fe	2502	285267	112759	263887	60667
Fe Pentacarbonyl	Fe	27	0	0	0	0
Fe Polysaccharid	Fe Polysaccharid	14	16347	9055	15085	1515
Fe Polysaccharide	Fe Polysaccharide	48	16347	9055	15085	1515
Felbamate	Felbamate	522	669	171	605	135
Female Condom	Female Condom	653	13	0	12	1
Fenfluramine	Fenfluramine	22	63	21	60	41
Fenofibrate	Fenofibrate	633	15	9	13	8
Fenofibric Acid	Fenofibric Acid	27	0	0	0	0
Fenoprofen	Fenoprofen	246	2	1	2	0
Fentanyl	Fentanyl	127	1087	175	973	623
Ferrous	Ferrous	4491	22840	12916	20845	3174
Ferrous Asparto G	Ferrous Asparto G	27	0	0	0	0
Ferrous Bisglycin	Ferrous Bisglycin	1761	4	2	3	4
Ferrous Fum	Ferrous Fum	156654	16	8	12	11
Fesoterodine	Fesoterodine	84	30	17	23	20
Fexofenadine	Fexofenadine	2928	45	1	34	36
Fidaxomicin	Fidaxomicin	117	6	2	6	3
Filgrastim	Filgrastim	158	901	416	866	417
Filgrastim-aafi	Filgrastim-aafi	27	0	0	0	0
Filgrastim-sndz	Filgrastim-sndz	90	0	0	0	0
Finasteride	Finasteride	246	14	3	10	7
Fingolimod	Fingolimod	88	34	7	32	11
Fish Oil	Fish Oil	20	679	433	598	292
Flavoring Aid	Flavoring Aid	53	115	61	108	74
Flavoxate	Flavoxate	61	5	0	5	1
Flecainide	Flecainide	311	65	17	64	27
Fluconazole	Fluconazole	114690	416	58	385	112
Fludrocortisone	Fludrocortisone	7297	102	43	101	17
Flunisolide	Flunisolide	886	46	6	27	44
Fluocinolone Acetonide	Fluocinolone Acetonide	20691	77	4	59	66
Fluocinonide	Fluocinonide	25984	13	0	13	11
Fluoride	Fluoride	145	126952	51885	116938	27115
Fluorometholone	Fluorometholone	3320	15	1	14	9
Fluorouracil	Fluorouracil	3505	405	90	381	91
Fluoxetine	Fluoxetine	234847	735	121	661	313
Fluphenazine	Fluphenazine	236	16	3	15	2
Flurandrenolide	Flurandrenolide	1953	2	0	2	1
Flurazepam	Flurazepam	11	3	2	1	1
Flurbiprofen	Flurbiprofen	991	18	4	17	13
Fluticasone	Fluticasone	454941	679	107	447	604
Fluticasone Furoate	Fluticasone	10885	48	9	22	45
Fluvoxamine	Fluvoxamine	8452	67	13	63	29
Folate Combinat	Folate Combinat	4393	2	0	2	1
Folic	Folic	46	14388	7477	13191	3306
Folic A	Folic A	245	9242	5007	8511	1878
Folic Ac	Folic Ac	39	1821	1037	1652	410
Folic Acid	Folic Acid	24306	14383	7474	13186	3306
Follicle Stimulating Hormone	Follicle Stimulating Hormone	16	3839	2540	3084	805
Follitropin Alfa	Follitropin Alfa	23	170	54	149	62
Fondaparinux	Fondaparinux	19	12	4	12	7
Formaldehyde	Formaldehyde	323	2220	950	2032	490
Formoterol	Formoterol	39424	464	131	315	273

Fosfomycin Tromethamine	Fosfomycin Tromethamine	64	101	23	89	27
Fremanezumab-vfrm	Fremanezumab-vfrm	165	1	0	1	0
Frovatriptan	Frovatriptan	573	3	1	1	2
Furosemide	Furosemide	1841	305	105	286	100
Gabapentin	Gabapentin	28485	181	32	167	65
Gabapentin Enacarbil	Gabapentin Enacarbil	67	0	0	0	0
Galantamine Hydrobromide	Galantamine	46	6	1	6	2
Galcanezumab-gnlm	Galcanezumab-gnlm	277	2	0	1	1
Ganciclovir	Ganciclovir	460	387	131	369	137
Gatifloxacin	Gatifloxacin	635	44	7	38	19
Gemfibrozil	Gemfibrozil	315	64	15	61	21
Gentamicin	Gentamicin	16599	862	193	804	242
GG	GG	598	116465	48971	106939	22569
Glatiramer	Glatiramer	93	70	4	69	22
Glimepiride	Glimepiride	140	4	3	3	1
Glipizide	Glipizide	121	7	6	7	0
Glucagon	Glucagon	26586	714	527	622	205
Glucose Meter	Glucose Meter	4081	51	38	35	14
Glutamine	Glutamine	58	266781	106002	246585	55575
Glyburide	Glyburide	136	39	18	39	10
Glycerin	Glycerin	61	286865	113364	265302	60008
Glycerol Phenylbutyrate	Glycerol Phenylbutyrate	34	26	20	25	9
Glycolic Acid	Glycolic Acid	20	88	22	71	31
Glycopyrrolate	Glycopyrrolate	10408	56	8	50	38
Glycopyrronium	Glycopyrronium	2994	55	8	49	38
Golimumab	Golimumab	60	27	9	25	18
Gramicidin	Gramicidin	329	43	10	38	17
Granisetron	Granisetron	566	46	14	45	39
Griseofulvin	Griseofulvin	4510	68	3	68	28
Griseofulvin, Ultramicrocrystalline	Griseofulvin, Ultramicrocrystalline	1854	68	3	68	28
Guaifenesin	Guaifenesin	28674	11178	4211	10186	2888
Guanfacine	Guanfacine	102483	89	12	84	58
Guselkumab	Guselkumab	16	0	0	0	0
Halcinonide	Halcinonide	687	12	0	12	11
Halobetasol	Halobetasol	3269	3	0	3	2
Haloperidol	Haloperidol	1356	261	55	236	56
Haloperidol Decanoate	Haloperidol Decanoate	18	1	1	1	0
HC	HC	74654	16373	8374	14833	2793
HC Ace	HC Ace	904	0	0	0	0
Heparin	Heparin	123	44331	20786	40383	10710
Histrelin	Histrelin	142	8	1	8	4
Homatropine Hydrobromide	Homatropine	91	0	0	0	0
Homatropine Methylbromide	Homatropine Methylbromide	5383	3	1	3	3
Hyaluronate	Hyaluronate	124	694	340	606	148
Hyaluronidase	Hyaluronidase	15	70	42	66	11
Hydralazine	Hydralazine	155	86	17	84	38
Hydrochlorothiazide	Hydrochlorothiazide	3147	437	153	399	177
Hydrocod Bit	Hydrocod Bit	31	0	0	0	0
Hydrocodone Bitartrate	Hydrocodone	407479	1691	374	1574	575
Hydrocodone Polistirex	Hydrocodone	4499	0	0	0	0
Hydrocortisone	Hydrocortisone	84439	8570	3845	7854	2752

Hydrocortisone Probutate	Hydrocortisone	33	0	0	0	0
Hydrocortisone Sodium	Hydrocortisone	1262	2872	1995	2497	639
Hydrocortisone Valerate	Hydrocortisone	7169	1	0	1	1
Hydrocortisone Butyrate	Hydrocortisone Butyrate	3551	2872	1995	2497	639
Hydromorphone	Hydromorphone	1687	68	10	60	31
Hydroquinone	Hydroquinone	316	2802	543	2482	1167
Hydroxocobalam	Hydroxocobalam	24	20	13	19	2
Hydroxocobalamin	Hydroxocobalamin	35	24	16	23	3
Hydroxychloroquine	Hydroxychloroquine	4625	232	37	222	48
Hydroxyurea	Hydroxyurea	1347	25478	14975	22720	5735
Hydroxyzine	Hydroxyzine	104851	47	6	39	28
Hydroxyzine Pamoate	Hydroxyzine	42822	5	0	4	3
Hyoscyamine	Hyoscyamine	25086	29327	12562	27036	6486
Hyoscyamine Sulf	Hyoscyamine Sulf	515	0	0	0	0
Hypochlorous Acid	Hypochlorous Acid	242	68	6	62	20
Hypromellose	Hypromellose	29	33	16	29	9
Ibuprofen	Ibuprofen	397582	38326	15450	35297	5872
Icatibant	Icatibant	18	7	4	7	2
Icosapent Ethyl	Icosapent Ethyl	104	2	0	2	0
IF	IF	20	237547	97340	218715	50599
Iloperidone	Iloperidone	150	0	0	0	0
Imatinib Mesylate	Imatinib	75	385	68	363	112
Imiglucerase	Imiglucerase	16	49	34	48	27
Imipramine	Imipramine	3126	348	166	312	116
Imipramine Pamoate	Imipramine	124	0	0	0	0
Imiquimod	Imiquimod	13340	54	1	47	25
Immune Globulin	Immune Globulin	689	252902	105958	232959	54210
Immune Globulin-klhw	Immune Globulin-klhw	15	19	18	14	2
Indapamide	Indapamide	13	0	0	0	0
Indomethacin	Indomethacin	4632	636	146	604	131
Infliximab	Infliximab	491	960	312	897	408
Infliximab-dyyb	Infliximab-dyyb	24	805	206	769	394
Infusion Pump, Insulin	Infusion Pump, Insulin	1273	470	287	403	217
Infusion Pump, Parenteral	Infusion Pump, Parenteral	440	0	0	0	0
Insuli	Insuli	16	14172	10661	12754	3059
Insulin Aspart	Insulin Aspart	25711	2676	1998	2427	474
Insulin Aspart Protamine	Insulin Aspart Protamine	154	2674	1997	2425	473
Insulin Degludec	Insulin Degludec	4025	20	13	18	17
Insulin Detemir	Insulin Detemir	3260	38	30	36	31
Insulin Glargine	Insulin Glargine	22013	92	67	85	76
Insulin Glulisine	Insulin Glulisine	329	6	5	5	5
Insulin Human Inhaled	Insulin Human Inhaled	39	0	0	0	0
Insulin Human Isophane	Insulin Human Isophane	447	3	2	2	3
Insulin Human Regular	Insulin Human Regular	198	14165	10655	12747	3059
Insulin Lispro	Insulin Lispro	21187	63	43	56	48
Insulin Lispro Protamine	Insulin Lispro Protamine	133	57	39	50	45
Insulin Lispro-aabc	Insulin Lispro-aabc	27	0	0	0	0
Interferon Beta-1A	Interferon Beta-1A	41	39	2	38	21
Interferon Gamma-1B	Interferon Gamma-1B	21	5	2	4	4
Inulin	Inulin	17	211	162	199	54
Iodine	Iodine	98	104558	46182	96288	19469
Iodoquinol	Iodoquinol	416	5	0	5	2
Iohexol	Iohexol	11	162	70	158	38
Ipratropium Bromide	Ipratropium	22588	121	5	104	98

Irbesartan	Irbesartan	123	15	6	12	12
Irinotecan	Irinotecan	18	1280	258	1239	591
Iron	Iron	207	289159	114512	267507	60990
Iron Polysaccharide	Iron Polysaccharide	200	16347	9055	15085	1515
Iron Sucrose	Iron Sucrose	27	16339	9055	15077	1515
Isavuconazonium	Isavuconazonium	20	2	1	2	2
Isometheptene Mucate	Isometheptene Mucate	772	1287	329	1183	396
Isoniazid	Isoniazid	786	27470	10392	24743	9284
Isopropyl Alcohol	Isopropyl Alcohol	1200	9944	2309	9450	1092
Isotretinoin	Isotretinoin	133096	274	45	261	78
Isradipine	Isradipine	153	6	2	6	4
Itraconazole	Itraconazole	1323	537	132	489	150
Ivabradine	Ivabradine	229	8	3	8	5
Ivacaftor	Ivacaftor	1497	135	18	121	57
Ivermectin	Ivermectin	11729	902	66	874	381
Ixekizumab	Ixekizumab	114	3	0	3	1
K Cl	K Cl	792	362	98	335	96
K Oxybate	K Oxybate	21	0	0	0	0
K Phos	K Phos	534	5	2	5	2
Ketamine	Ketamine	30	574	54	548	291
Ketoconazole	Ketoconazole	128244	199	33	175	80
Ketoprofen	Ketoprofen	230	54	9	46	33
Ketorolac Tromethamine	Ketorolac Tromethamine	28453	151	14	125	99
Ketotifen	Ketotifen	706	105	16	88	80
L-Methylfolate	L-Methylfolate	935	6	2	4	5
Labetalol	Labetalol	561	118	42	109	49
Lacosamide	Lacosamide	2868	66	28	60	40
Lactic Acid	Lactic Acid	44	1813	1216	1615	241
Lactobacillus Acidophilus	Lactobacillus Acidophilus	192	6101	3788	5554	1244
Lactobacillus Bulgaricus	Lactobacillus Bulgaricus	15	2	0	2	1
Lactobacillus c	Lactobacillus c	131	68	21	48	37
Lactobacillus casei	Lactobacillus casei	34	2728	1340	2504	805
Lactobacillus rhamnosus GG	Lactobacillus rhamnosus GG	41	151	35	141	63
Lactulose	Lactulose	4035	186	115	184	66
Lamivudine	Lamivudine	164	635	281	594	396
Lamotrigine	Lamotrigine	54815	499	162	459	207
Lanadelumab-flyo	Lanadelumab-flyo	21	2	1	2	2
Lancet	Lancet	13156	23	7	20	6
Lansoprazole	Lansoprazole	17211	75	22	69	50
Lasmiditan	Lasmiditan	16	1	1	1	1
Latanoprost	Latanoprost	1541	37	1	32	30
Lauric Acid	Lauric Acid	35	112348	43405	103571	24556
Lecithin	Lecithin	11	3272	1603	2922	827
Ledipasvir	Ledipasvir	17	38	3	38	28
Leflunomide	Leflunomide	524	79	16	78	22
Lenalidomide	Lenalidomide	12	24	4	22	10
Letermovir	Letermovir	20	7	3	6	3
Letrozole	Letrozole	1712	73	25	65	57
Leucovorin	Leucovorin	1691	382	163	372	151
Leuprolide	Leuprolide	1054	136	50	105	70
Levalbuterol	Levalbuterol	26918	20	2	13	20
Levetiracetam	Levetiracetam	22201	427	117	406	232
Levocarnitine	Levocarnitine	2370	553	442	509	142

Levocetirizine Dihydrochloride	Levocetirizine	36832	26	1	14	20
Levodopa	Levodopa	456	460	276	438	96
Levofloxacin	Levofloxacin	13013	260	31	243	61
Levomefolate	Levomefolate	1616	163	16	96	124
Levomefolate Ca	Levomefolate Ca	4476	3	2	1	3
Levomilnacipran	Levomilnacipran	214	0	0	0	0
Levonorgestrel	Levonorgestrel	103087	1059	156	863	563
Levothyroxine	Levothyroxine	58921	10317	6866	9157	1103
Lidocaine	Lidocaine	57601	737	71	660	414
Lifitegrast	Lifitegrast	205	1	0	1	0
Linaclotide	Linaclotide	1935	0	0	0	0
Linagliptin	Linagliptin	16	6	1	6	2
Lindane	Lindane	193	137	109	122	13
Linezolid	Linezolid	495	141	26	124	38
Liothyronine	Liothyronine	1189	10738	6899	9558	1256
Lipase	Lipase	2609	290102	115833	268287	60611
Liraglutide	Liraglutide	718	37	11	35	15
Lisdexamfetamine Dimesylate	Lisdexamfetamine Dimesylate	211769	68	2	64	38
Lisinopril	Lisinopril	12268	102	46	92	44
Lithium	Lithium	12061	410	155	375	95
Lodoxamide Tromethamine	Lodoxamide Tromethamine	34	6	0	5	6
Lomustine	Lomustine	20	228	28	225	87
Loperamide	Loperamide	1692	67	12	61	23
Lopinavir	Lopinavir	31	186	100	173	116
Loratadine	Loratadine	11817	134	7	89	118
Lorazepam	Lorazepam	27920	152	41	137	54
Lorcaserin	Lorcaserin	30	1	0	0	1
Losartan	Losartan	3205	137	49	123	79
Loteprednol Etabonate	Loteprednol	7954	8	0	6	5
Lovastatin	Lovastatin	135	36	14	34	18
Loxapine	Loxapine	150	9	4	9	1
Lubiprostone	Lubiprostone	1248	4	0	4	3
Lubricant	Lubricant	2485	4689	1427	4414	1072
Luliconazole	Luliconazole	561	3	0	2	2
Lumacaftor	Lumacaftor	485	49	9	47	18
Lumefantrine	Lumefantrine	53	334	75	317	222
Lurasidone	Lurasidone	10007	20	2	18	12
Luteinizing Hormone	Luteinizing Hormone	16	5758	3477	4848	1138
Macitentan	Macitentan	30	7	3	4	6
Mafenide	Mafenide	13	7	1	7	2
Magnesium	Magnesium	2283	111150	49329	101299	31510
Magnesium Hydroxide	Magnesium Hydroxide	179	27463	14324	24829	4997
Magnesium Oxide	Magnesium Oxide	714	17948	8813	16076	4728
Malathion	Malathion	2563	52	17	46	16
Mebendazole	Mebendazole	845	225	19	223	97
Mecasermin	Mecasermin	47	40	26	37	27
Meclizine	Meclizine	6536	9	0	8	1
Meclofenamate	Meclofenamate	114	7	1	2	6
Medroxyprogesterone	Medroxyprogesterone	51431	126162	51848	114915	32791
Mefenamic Acid	Mefenamic Acid	2894	909	205	819	353
Mefloquine	Mefloquine	4977	549	130	503	373
Megestrol	Megestrol	552	82	26	67	46
Melatonin	Melatonin	310	6518	4017	5935	1304

Meloxicam	Meloxicam	59253	23	2	20	14
Memantine	Memantine	948	17	1	11	8
Menthol	Menthol	43	681	74	639	140
Meperidine	Meperidine	1128	212	26	189	134
Mepolizumab	Mepolizumab	151	36	3	27	21
Mercaptopurine	Mercaptopurine	2437	8502	4755	7796	2032
Meropenem	Meropenem	17	233	60	212	72
Mesalamine	Mesalamine	7461	227	49	218	85
Mesna	Mesna	25	129	28	128	74
Mestranol	Mestranol	365	130	60	115	58
Metaxalone	Metaxalone	4393	0	0	0	0
Meter, Peak Flow, Spirometry	Meter, Peak Flow, Spirometry	105	0	0	0	0
Metformin	Metformin	30209	643	265	536	370
Methadone	Methadone	241	365	69	346	88
Methamphetamine	Methamphetamine	21	129772	42697	118903	35040
Methazolamide	Methazolamide	61	0	0	0	0
Methenamine	Methenamine	200	28904	12532	26627	6265
Methenamine Hippurate	Methenamine Hippurate	69	15	7	14	2
Methenamine Mandelate	Methenamine Mandelate	11	1	0	0	1
Methimazole	Methimazole	3170	221	127	194	36
Methocarbamol	Methocarbamol	13940	811	174	776	247
Methotrexate	Methotrexate	10012	4119	1018	4043	1736
Methscopolamine Bromide	Methscopolamine	225	1407	695	1252	305
Methsuximide	Methsuximide	49	12	10	11	5
Methylcobalamin	Methylcobalamin	168	734	599	612	81
Methyldopa	Methyldopa	14	375	140	332	121
Methylene Blue	Methylene Blue	195	28940	12543	26659	6271
Methylergonovine	Methylergonovine	119	14	1	13	4
Methylparaben	Methylparaben	15	858	389	815	257
Methylphenidate	Methylphenidate	300562	36087	14332	33955	7508
Methylprednisolone	Methylprednisolone	198810	3509	1008	3351	1413
Methylprednisolone Sodium	Methylprednisolone	93	1538	413	1485	583
Metoclopramide	Metoclopramide	7642	1106	267	992	442
Metolazone	Metolazone	44	2	1	2	2
Metoprolol	Metoprolol	4899	154	49	142	71
Metronidazole	Metronidazole	63927	945	80	848	401
Mexiletine	Mexiletine	162	31	9	31	9
Mg Hydroxide	Mg Hydroxide	1638	11548	4879	10233	3460
Mg Oxybate	Mg Oxybate	21	52	17	44	17
Mg Sulf	Mg Sulf	80	165	90	135	38
Miconazole Nitrate	Miconazole	181	1815	362	1673	576
Midazolam	Midazolam	3829	645	122	605	375
Midodrine	Midodrine	2746	22	4	20	15
Milk Protein	Milk Protein	22	801	628	739	240
Milnacipran	Milnacipran	39	3	0	3	1
Mineral Oil	Mineral Oil	16	22	5	20	9
Minocycline	Minocycline	201087	264	26	252	63
Minoxidil	Minoxidil	107	219	53	211	67
Mirabegron	Mirabegron	431	11	1	10	10
Mirtazapine	Mirtazapine	19781	39	5	37	9
Misoprostol	Misoprostol	5612	490	28	457	343
Modafinil	Modafinil	1222	48	2	47	24

Mometasone Furoate	Mometasone	91880	131	8	73	120
Montelukast	Montelukast	282258	327	55	228	282
Morphine	Morphine	1214	293718	116398	271806	61301
Moxifloxacin	Moxifloxacin	22229	112	16	95	37
Mupirocin	Mupirocin	242173	70	0	65	42
Mycophenolate	Mycophenolate	456	11752	3835	10984	2243
Mycophenolate Mofetil	Mycophenolate Mofetil	3105	835	296	802	365
Na Bicarb	Na Bicarb	634	11587	4845	10277	3476
Na Cl	Na Cl	810	156	50	144	37
Na Fluoride	Na Fluoride	21	0	0	0	0
Na Oxybate	Na Oxybate	21	0	0	0	0
Na Pho	Na Pho	195	3795	1054	3650	1455
Na Phos	Na Phos	634	3795	1054	3650	1455
Na Phos, Mo	Na Phos, Mo	34	0	0	0	0
Na Phos, Monoba	Na Phos, Monoba	108	0	0	0	0
Na Sulf	Na Sulf	275	0	0	0	0
Nabumetone	Nabumetone	3346	6	0	6	4
Nadolol	Nadolol	2197	25	3	24	10
Nafarelin	Nafarelin	24	23	10	13	11
Naftifine	Naftifine	1517	6	1	6	6
Naloxegol	Naloxegol	39	0	0	0	0
Naloxone	Naloxone	1121	297	53	260	92
Naltrexone	Naltrexone	2749	376	122	347	106
Naphazoline	Naphazoline	33	5	0	3	4
Naproxen	Naproxen	151644	276	99	248	92
Naratriptan	Naratriptan	1615	5	1	5	1
Natamycin	Natamycin	25	33	1	32	17
Nebivolol	Nebivolol	182	76	34	66	32
Nebulizer, Direct Patient Interface	Nebulizer, Direct Patient Interface	152	0	0	0	0
Nedocromil	Nedocromil	66	106	12	73	99
Nefazodone	Nefazodone	36	13	4	12	4
Neomycin	Neomycin	24737	263	17	253	121
Neomycin Sulf	Neomycin Sulf	75535	14	0	14	2
Nepafenac	Nepafenac	87	0	0	0	0
Netarsudil	Netarsudil	50	3	0	3	2
Niacin	Niacin	101	23397	12810	21587	3036
Niacinamide	Niacinamide	1644	7827	4179	7234	1577
Nicotine	Nicotine	764	4247	852	4116	534
Nicotine Polacrilex	Nicotine Polacrilex	232	2003	320	1951	73
Nifedipine	Nifedipine	1085	448	195	389	187
Nimodipine	Nimodipine	25	26	1	26	7
Nitazoxanide	Nitazoxanide	839	32	1	29	22
Nitrofurantoin	Nitrofurantoin	68074	149	5	145	32
Nitrofurantoin Monohydrate	Nitrofurantoin Monohydrate	62692	147	5	143	32
Nitroglycerin	Nitroglycerin	465	286850	113354	265287	60000
Nizatidine	Nizatidine	254	9	1	9	4
Norelgestromin	Norelgestromin	20018	31	8	17	25
Norethindrone	Norethindrone	148550	3720	2124	2787	1375
Norethindrone Ace	Norethindrone Ace	157981	85	20	69	56
Norflurane	Norflurane	48	320	27	287	187
Norgestimate	Norgestimate	246636	81	13	59	48
Norgestrel	Norgestrel	21168	1113	184	905	614
Nortriptyline	Nortriptyline	7355	230	57	202	81
Nusinersen	Nusinersen	36	55	10	55	22

Nutriceutical	Nutriceutical	16	11	4	10	7
Nystatin	Nystatin	35083	753	308	664	224
Octinoxate	Octinoxate	93	14	3	12	1
Octisalate	Octisalate	93	0	0	0	0
Octreotide	Octreotide	11	199	75	180	44
Ofloxacin	Ofloxacin	79705	6296	3379	5830	1170
Olanzapine	Olanzapine	11794	309	60	279	122
Olmesartan Medoxomil	Olmesartan Medoxomil	66	99	42	88	35
Olodaterol	Olodaterol	25	0	0	0	0
Olopatadine	Olopatadine	45852	29	1	21	26
Omalizumab	Omalizumab	2150	343	87	293	226
Omega-3 Fatty Acids	Omega-3 Fatty Acids	183	672	458	590	268
Omega-3-Acid Ethyl Esters	Omega-3-Acid Ethyl Esters	754	163	97	132	84
Omeprazole	Omeprazole	95563	1141	257	1083	431
OnabotulinumtoxinA	OnabotulinumtoxinA	1125	24	3	24	18
Ondansetron	Ondansetron	404312	243	25	217	181
Orchar	Orchar	57	35	11	29	4
Orphenadrine	Orphenadrine	1386	811	176	775	247
Oseltamivir	Oseltamivir	287095	452	41	437	125
Ostomy Product	Ostomy Product	11	0	0	0	0
Oxandrolone	Oxandrolone	294	104	41	103	88
Oxaprozin	Oxaprozin	278	1	0	1	1
Oxazepam	Oxazepam	39	51	10	41	27
Oxcarbazepine	Oxcarbazepine	24823	245	110	229	120
Oxiconazole Nitrate	Oxiconazole	1486	2	0	2	2
Oxybutynin	Oxybutynin	9245	111	10	108	73
Oxycodone	Oxycodone	145807	1989	425	1832	719
Oxymetazoline	Oxymetazoline	655	24010	7711	22110	8442
Oxyquinoline	Oxyquinoline	13	3401	2278	2954	742
Ozenoxacin	Ozenoxacin	125	6	0	6	5
p-Phenolsulfonic Acid	p-Phenolsulfonic Acid	47	0	0	0	0
Paliperidone	Paliperidone	1898	72	38	61	25
Pantoprazole	Pantoprazole	24367	70	23	66	27
Paricalcitol	Paricalcitol	30	7	4	7	5
Paromomycin	Paromomycin	53	69	7	66	54
Paroxetine	Paroxetine	13520	148	31	133	45
Paroxetine Mesylate	Paroxetine	20	0	0	0	0
Patiromer	Patiromer	13	0	0	0	0
Pazopanib	Pazopanib	63	17	5	17	5
PEG	PEG	124	2449	851	2209	837
PEG Electrolyte Lavage Solution	PEG Electrolyte Lavage Solution	1217	0	0	0	0
Pegfilgrastim	Pegfilgrastim	361	30	18	29	25
Pegfilgrastim-bmez	Pegfilgrastim-bmez	30	24	12	24	20
Pegfilgrastim-cbqv	Pegfilgrastim-cbqv	21	24	12	24	20
Pegfilgrastim-jmdb	Pegfilgrastim-jmdb	37	24	12	24	20
Peginterferon Beta-1a	Peginterferon Beta-1a	16	0	0	0	0
Pegvaliase-pqpz	Pegvaliase-pqpz	17	4	3	1	0
Penciclovir	Penciclovir	836	720	134	699	182
Penicillin G	Penicillin G	12	7059	4733	6307	1577
Penicillin G Benzathine	Penicillin G Benzathine	148	135	19	128	53
Penicillin G Procaine	Penicillin G Procaine	27	33	5	26	14
Penicillin V	Penicillin V	92326	4369	1144	4002	962
Pentazocine	Pentazocine	35	1092	223	996	396
Pentosan Polysulfate	Pentosan Polysulfate	123	3	0	3	2

Pentoxifylline	Pentoxifylline	146	66	11	62	31
Perampanel	Perampanel	725	77	16	63	56
Permethrin	Permethrin	17833	128	23	114	68
Perphenazine	Perphenazine	269	21	10	14	3
Petrolatum	Petrolatum	21	136	18	124	54
Petrolatum, White	Petrolatum, White	51	84	13	77	39
Phenazopyridine	Phenazopyridine	22940	18	8	18	6
Phendimetrazine	Phendimetrazine	23	1	0	1	0
Pheniramine	Pheniramine	19	92	20	81	51
Phenobarb	Phenobarb	315	724	410	653	263
Phenobarbital	Phenobarbital	708	1105	559	999	340
Phenol	Phenol	12	96389	33978	89073	20816
Phentermine	Phentermine	608	8	1	7	4
Phenyleph	Phenyleph	4326	926	332	800	360
Phenylephrine	Phenylephrine	152	11643	4925	10100	2395
Phenytoin	Phenytoin	345	1551	883	1377	390
Phosphate	Phosphate	27	135853	56007	125609	25876
Phosphorus	Phosphorus	23	6849	4259	6256	1303
Phytonadione	Phytonadione	470	339	171	317	91
Pilocarpine	Pilocarpine	102	55	20	50	4
Pimecrolimus	Pimecrolimus	15571	57	3	56	46
Pimozide	Pimozide	402	88	19	85	25
Pindolol	Pindolol	103	31	17	30	6
Pioglitazone	Pioglitazone	139	111626	43720	104590	20358
Piperacillin	Piperacillin	16	332	90	307	140
Piroxicam	Piroxicam	976	26	3	23	17
Pitolisant	Pitolisant	12	6	1	6	4
Plecanatide	Plecanatide	93	0	0	0	0
Podofilox	Podofilox	756	192	55	181	61
Polyethylene G	Polyethylene G	33	578	241	523	312
Polyethylene Glycol	Polyethylene Glycol	18	5546	2165	5178	1398
Polyethylene Glycol 3350	Polyethylene Glycol 3350	28046	28	6	28	25
Polymyxin B	Polymyxin B	80635	74	6	69	46
Polymyxin B Sulf	Polymyxin B Sulf	74652	7	0	7	6
Polyureaurethane	Polyureaurethane	140	0	0	0	0
Polyvinyl Alcohol	Polyvinyl Alcohol	13	31	2	30	6
Ponatinib	Ponatinib	11	7	0	7	3
Posaconazole	Posaconazole	107	107	43	88	32
Potassium	Potassium	4772	257444	104184	237934	54262
Potassium Bitartrate	Potassium	11	0	0	0	0
Potassium Nitrate	Potassium	5655	60	6	57	19
Potassium Bicarbonate	Potassium Bicarbonate	49	6	3	6	3
Potassium Iodide	Potassium Iodide	95	95245	41412	87825	18138
Pramipexole Dihydrochloride	Pramipexole	279	7	1	7	4
Pramlintide	Pramlintide	11	8	6	5	8
Pramoxine	Pramoxine	929	10	2	9	6
Pravastatin	Pravastatin	734	855	203	818	279
Praziquantel	Praziquantel	96	768	134	759	303
Prazosin	Prazosin	9247	654	352	541	77
Prednicarbate	Prednicarbate	219	8	0	8	5
Prednisolone	Prednisolone	27307	5218	1479	5006	2039
Prednisolone Sodium	Prednisolone	17895	3676	972	3547	1427
Prednisone	Prednisone	449733	3945	1012	3835	1647
Pregabalin	Pregabalin	1815	57	6	47	27

Prenata	Prenata	54	5586	1956	5453	555
Prilocaine	Prilocaine	12128	143	8	126	97
Primaquine	Primaquine	30	233	63	214	110
Primidone	Primidone	235	109	75	93	34
Probenecid	Probenecid	34	54	27	42	20
Prochlorperazine	Prochlorperazine	5721	3166	960	2986	738
Progesterone	Progesterone	3024	289039	114502	267555	60886
Progestin	Progestin	4393	285594	112704	264603	60327
Proguanil	Proguanil	16919	106	29	103	56
Promethazine	Promethazine	139505	941	207	859	362
Propafenone	Propafenone	25	39	10	38	16
Propantheline Bromide	Propantheline	119	15	0	15	4
Proparacaine	Proparacaine	50	10	0	10	7
Propranolol	Propranolol	30865	594	251	538	207
Propylthiouracil	Propylthiouracil	59	3125	871	2909	536
Protease	Protease	2602	279478	109232	258712	58703
Protriptyline	Protriptyline	75	5	1	4	1
Prucalopride	Prucalopride	123	4	0	3	4
PSE	PSE	162799	16387	4272	15734	4698
Pseudoephedrine	Pseudoephedrine	4063	31613	13469	28784	7180
Pump, Infusion	Pump, Infusion	206	779	374	694	350
Pyrazinamide	Pyrazinamide	25	923	153	878	216
Pyridostigmine Bromide	Pyridostigmine	617	122	53	118	28
Pyridoxal Phos	Pyridoxal Phos	66	95	85	79	17
Pyridoxine	Pyridoxine	1028	265589	104833	247340	55783
Pyrilamine	Pyrilamine	264	29	0	22	19
Quetiapine	Quetiapine	34358	234	43	212	104
Quinapril	Quinapril	14	76	34	67	34
Quinidine	Quinidine	149	64	16	58	11
Quinine	Quinine	13	566	238	490	185
Rabeprazole	Rabeprazole	613	53	15	50	24
Raloxifene	Raloxifene	27	69	11	64	2
Raltegravir	Raltegravir	341	342	184	311	223
Ramelteon	Ramelteon	233	5	0	4	3
Ramipril	Ramipril	51	32	7	30	24
Ranitidine	Ranitidine	34304	130	24	123	65
Reduced Diphtheria Toxoid	Reduced Diphtheria Toxoid	4482	61	8	57	31
Regorafenib	Regorafenib	16	5	3	4	1
Repaglinide	Repaglinide	22	15	5	12	5
Retapamulin	Retapamulin	431	13	0	12	10
Rho(D) Immune Globulin	Rho(D) Immune Globulin	13	143	103	133	71
Ribavirin	Ribavirin	12	334	107	311	140
Riboflavin	Riboflavin	146	26546	14286	24402	3856
Rifabutin	Rifabutin	72	16	6	15	10
Rifampin	Rifampin	1779	1474	214	1396	347
Rifapentine	Rifapentine	78	23	4	21	14
Rifaximin	Rifaximin	1809	37	10	33	23
Rilpivirine	Rilpivirine	39	25	13	21	13
Riluzole	Riluzole	16	7	2	6	6
Rimantadine	Rimantadine	19	25	1	24	12
Rimegepant	Rimegepant	235	1	1	1	1
Risankizumab-rzaa	Risankizumab-rzaa	26	0	0	0	0
Risdiplam	Risdiplam	23	4	2	3	2
Risedronate	Risedronate	11	6066	3783	5533	1211
Risperidone	Risperidone	33849	621	131	583	290

Ritonavir	Ritonavir	60	342	192	311	219
Rituximab	Rituximab	11	895	274	866	320
Rivaroxaban	Rivaroxaban	878	32	11	30	8
Rizatriptan	Rizatriptan	34180	9	1	7	7
Romiplostim	Romiplostim	31	34	11	34	25
Ropinirole	Ropinirole	362	7	3	6	4
Rosuvastatin	Rosuvastatin	652	64	37	59	32
Rufinamide	Rufinamide	783	44	16	42	37
Ruxolitinib	Ruxolitinib	103	34	11	33	21
Saccharin	Saccharin	11	48	15	45	19
Saccharomyces Boulardii	Saccharomyces Boulardii	12	10	0	10	8
Sacrosidase	Sacrosidase	230	3	1	3	1
Sacubitril	Sacubitril	29	3	2	3	2
Salicylic Acid	Salicylic Acid	3718	26237	9280	24165	5847
Salmeterol Xinafoate	Salmeterol Xinafoate	34243	240	17	135	220
Salsalate	Salsalate	27	0	0	0	0
Sapropterin Dihydrochloride	Sapropterin	297	46	34	45	20
Sarecycline	Sarecycline	6135	1	0	1	1
Sargramostim	Sargramostim	22	361	223	314	153
Sch	Sch	38	156878	62525	150165	38365
Schi	Schi	27	3155	1125	2861	597
Schizochytrium	Schizochytrium	1291	4	1	4	3
Scop Hydr	Scop Hydr	315	0	0	0	0
Scopolamine	Scopolamine	10686	97	12	87	50
Secnidazole	Secnidazole	85	9	0	5	7
Secukinumab	Secukinumab	90	9	0	8	6
Segesterone	Segesterone	196	3	0	2	3
Selegiline	Selegiline	42	12	2	11	7
Selenium	Selenium	555	6912	4496	6291	1325
Selenium Sulfide	Selenium Sulfide	9361	7	1	7	4
Selexipag	Selexipag	38	5	0	5	0
Selumetinib	Selumetinib	43	16	6	15	11
Semaglutide	Semaglutide	222	135478	60164	124018	35492
Sennosides	Sennosides	237	20	3	20	9
Sennosides A and B	Sennosides A and B	26	20	3	20	9
Serdexmethylphenidate	Serdexmethylphenidate	49	0	0	0	0
Sertaconazole Nitrate	Sertaconazole	15	2	0	1	2
Sertraline	Sertraline	282480	261	30	236	108
Sevelamer	Sevelamer	220	14	11	14	8
Sildenafil	Sildenafil	312	117	21	109	77
Silodosin	Silodosin	27	2	0	2	1
Silver	Silver	11	290037	115290	268305	60719
Silver Nitrate	Silver	43	57	6	55	17
Silver Sulfadiazine	Silver Sulfadiazine	16005	79	3	72	46
Simethicone	Simethicone	1822	137	44	104	106
Simvastatin	Simvastatin	914	424	176	396	156
Sinecatechins	Sinecatechins	288	2	0	2	2
Sirolimus	Sirolimus	773	416	169	394	191
Sitagliptin	Sitagliptin	214	21	9	16	10
Sodium	Sodium	6897	290601	115180	268945	60565
Sodium Bicarbonate	Sodium Bicarbonate	319	20446	9236	18546	6067
Sodium Fluoride	Sodium Fluoride	72191	3956	1520	3620	1076
Sodium Oxybate	Sodium Oxybate	304	53	18	45	18
Sodium Picosulfate	Sodium Picosulfate	445	9	0	9	9

Sodium Polystyrene Sulfonate	Sodium Polystyrene Sulfonate	83	2	2	1	1
Sofosbuvir	Sofosbuvir	29	55	10	55	39
Solifenacin	Solifenacin	545	12	1	12	12
Solriamfetol	Solriamfetol	27	0	0	0	0
Somatropin, E-Coli Derived	Somatropin, E-Coli Derived	21755	90	30	80	74
Somatropin, Mammalian Derived	Somatropin, Mammalian Derived	147	44	15	43	35
Sorafenib	Sorafenib	62	56	14	55	21
Sorbitol	Sorbitol	47	223	113	202	58
Sotalol	Sotalol	156	503	118	489	88
Soy Protein	Soy Protein	17	47	23	38	23
Spacer, Inhalation	Spacer, Inhalation	26267	324	40	259	270
Spinosad	Spinosad	3561	2	0	2	2
Spironolactone	Spironolactone	40633	476	169	432	167
Squaric Acid Dibutylester	Squaric Acid Dibutylester	23	30	0	30	15
Stannous Fluoride	Stannous Fluoride	14	26	0	25	19
Stiripentol	Stiripentol	26	37	16	36	27
Succimer	Succimer	19	469	153	450	59
Succinic Acid	Succinic Acid	36	3148	1536	2897	461
Sucralfate	Sucralfate	16278	24	0	23	8
Sucrose	Sucrose	99	16962	9239	15670	1648
Sulconazole Nitrate	Sulconazole	316	1	0	1	1
Sulfacetamide	Sulfacetamide	65510	10	0	10	7
Sulfacetamide Na	Sulfacetamide Na	182	3	0	3	2
Sulfadiazine	Sulfadiazine	19	145	13	137	68
Sulfamethoxazole	Sulfamethoxazole	248724	2028	233	1959	510
Sulfasalazine	Sulfasalazine	1528	606	378	548	142
Sulfur	Sulfur	43686	147383	61504	134939	31869
Sulfuric Acid	Sulfuric Acid	47	3249	1628	2768	956
Sulindac	Sulindac	547	13	0	13	5
Sumatriptan	Sumatriptan	53836	67	10	54	40
Suvorexant	Suvorexant	138	2	0	2	1
Tacrolimus	Tacrolimus	16344	1465	644	1388	634
Tadalafil	Tadalafil	221	1161	218	1100	176
Tafluprost	Tafluprost	14	1	0	1	0
Tamoxifen	Tamoxifen	268	90	14	77	37
Tamsulosin	Tamsulosin	5868	27	4	21	18
Tapentadol	Tapentadol	136	10	2	10	3
Tavaborole	Tavaborole	344	2	1	2	2
Tazarotene	Tazarotene	46356	34	0	29	30
Tazobactam	Tazobactam	16	231	60	213	90
Tbo-Filgrastim	Tbo-Filgrastim	13	1	1	1	1
Tedizolid	Tedizolid	57	7	3	4	5
Telmisartan	Telmisartan	41	76	35	68	32
Temazepam	Temazepam	754	23	4	20	7
Temozolomide	Temozolomide	130	228	34	227	119
Tenofovir Al	Tenofovir Al	107	17	7	13	12
Tenofovir Alafenamide	Tenofovir Alafenamide	224	283	122	218	163
Tenofovir Disoproxil	Tenofovir Disoproxil	702	791	364	694	496
Tenofovir Disoproxil Fum	Tenofovir Disoproxil Fum	29	93	55	64	69
Terazosin	Terazosin	185	4	0	4	2
Terbinafine	Terbinafine	14305	77	5	74	39

Terbutaline	Terbutaline	16	245	31	180	206
Terconazole	Terconazole	4667	6	0	2	6
Teriparatide	Teriparatide	15	18	13	18	6
Testosterone	Testosterone	803	116499	43962	106793	28935
Testosterone Cypionate	Testosterone Cypionate	5653	14	5	12	7
Testosterone Enanthate	Testosterone Enanthate	456	49	25	42	28
Tetanus Toxoid	Tetanus Toxoid	71	75241	36427	68608	13706
Tetrabenazine	Tetrabenazine	109	26	5	26	9
Tetracaine	Tetracaine	331	63	5	61	47
Tetracycline	Tetracycline	1874	736	40	712	138
Tezacaftor	Tezacaftor	861	28	2	21	10
Thalidomide	Thalidomide	12	145	28	143	40
Theophylline	Theophylline	128	3570	1490	3102	1339
Thiamine	Thiamine	20	32918	15615	30236	4622
Thioctic	Thioctic	610	55	31	47	11
Thioctic Acid	Thioctic Acid	11	34	18	28	11
Thioguanine	Thioguanine	146	5598	3656	5013	1172
Thioridazine	Thioridazine	28	56	16	46	15
Thiothixene	Thiothixene	24	4	0	4	1
Thonzonium Brom	Thonzonium Brom	883	0	0	0	0
Thonzylamine	Thonzylamine	14	0	0	0	0
Thyroid	Thyroid	2651	135103	49780	126300	26248
Thyrotropin Alfa	Thyrotropin Alfa	21	570	363	487	84
Tiagabine	Tiagabine	56	26	7	26	18
Ticagrelor	Ticagrelor	13	8	4	4	6
Timolol	Timolol	2595	118	33	107	60
Tinidazole	Tinidazole	1608	1257	132	1185	359
Tiopronin	Tiopronin	33	51	30	47	7
Tiotropium Bromide	Tiotropium	2186	21	7	15	17
Tizanidine	Tizanidine	8440	8	0	7	7
Tobramycin	Tobramycin	50527	1383	517	1303	424
Tocilizumab	Tocilizumab	251	135	29	130	61
Tofacitinib	Tofacitinib	319	19	1	18	5
Tolmetin	Tolmetin	14	39	5	34	31
Tolterodine	Tolterodine	900	23	3	23	19
Topiramate	Topiramate	40424	534	183	497	267
Torsemide	Torsemide	27	133	38	113	48
Tramadol	Tramadol	38663	1215	244	1095	448
Trametinib Dimethyl Sulfoxide	Trametinib Dimethyl Sulfoxide	55	18	1	18	10
Tranexamic Acid	Tranexamic Acid	3568	202	38	182	92
Travoprost	Travoprost	209	6	1	6	6
Trazodone	Trazodone	62842	32	4	31	12
Treprostinil	Treprostinil	40	4851	1940	4455	805
Treprostinil Diolamine	Treprostinil Diolamine	15	33	9	32	24
Tretinoin	Tretinoin	351001	214435	86465	197093	51461
Triamcinolone Acetonide	Triamcinolone Acetonide	259950	343	29	309	174
Triamterene	Triamterene	178	364	135	338	127
Triazolam	Triazolam	11516	10	4	9	1
Trientine	Trientine	70	5203	2338	4731	1367
Trifarotene	Trifarotene	4728	4	0	3	4
Trifluoperazine	Trifluoperazine	42	11	3	10	1
Trifluridine	Trifluridine	297	39	16	35	11
Trihexyphenidyl	Trihexyphenidyl	411	38	5	36	10
Trimethobenzamide	Trimethobenzamide	117	27	21	26	1

Trimethoprim	Trimethoprim	305137	2234	304	2158	587
Triprolidine	Triprolidine	117	24143	11371	21850	5384
Triptorelin Pamoate	Triptorelin	81	89	39	74	63
Tropicamide	Tropicamide	75	48	3	43	31
Trospium	Trospium	123	2	0	2	2
Tuberculin	Tuberculin	35	1550	394	1511	206
Ubrogepant	Ubrogepant	180	0	0	0	0
Ulipristal	Ulipristal	566	7677	2474	7214	1341
Umeclidinium	Umeclidinium	253	3	0	0	3
Umeclidinium Bromide	Umeclidinium	33	0	0	0	0
Urea	Urea	1829	37516	19680	33930	7236
Ursodiol	Ursodiol	1798	7238	3192	6832	2093
Ustekinumab	Ustekinumab	1363	51	8	51	24
Valacyclovir	Valacyclovir	37921	42	12	38	20
Valbenazine	Valbenazine	14	1	0	1	1
Valganciclovir	Valganciclovir	437	68	30	67	32
Valproic Acid	Valproic Acid	1827	1925	928	1785	755
Valsartan	Valsartan	215	91	38	81	46
Vancomycin	Vancomycin	1842	952	254	888	276
Varenicline	Varenicline	99	14	2	9	6
Vedolizumab	Vedolizumab	81	38	14	35	20
Vemurafenib	Vemurafenib	11	11	2	11	4
Venlafaxine	Venlafaxine	27144	111	19	97	44
Verapamil	Verapamil	2111	167	32	159	46
Vigabatrin	Vigabatrin	94	163	36	156	99
Vilanterol	Vilanterol	159	21	4	6	19
Vilanterol Trifenatate	Vilanterol	5789	1	0	0	1
Vilazodone	Vilazodone	2019	5	1	5	3
Viloxazine	Viloxazine	444	14	1	12	12
Vitami	Vitami	54	8927	6230	7944	1845
Vitamin	Vitamin	209	281912	109448	260907	60997
Vitamin A	Vitamin A	19	251801	99610	232446	55715
Vitamin B	Vitamin B	43	32949	15572	30527	7040
Vitamin B Complex	Vitamin B Complex	11	3491	1887	3190	849
Vitamin B Complex and Vitamin C	Vitamin B Complex and Vitamin C	20	1	1	1	0
Vitamin B1	Vitamin B1	22	24093	13386	22174	3085
Vitamin B12	Vitamin B12	10118	125877	46518	116250	24421
Vitamin B2	Vitamin B2	6162	26542	14284	24399	3856
Vitamin B3	Vitamin B3	6762	1988	476	1922	433
Vitamin B5	Vitamin B5	58	54	18	48	23
Vitamin B6	Vitamin B6	7136	27078	14680	24863	3981
Vitamin C	Vitamin C	11231	57559	27092	52837	9753
Vitamin D	Vitamin D	223	149754	56656	138953	30154
Vitamin E	Vitamin E	110	25292	10492	23542	4002
Von Willebrand Factor	Von Willebrand Factor	154	1833	1013	1740	452
Voriconazole	Voriconazole	381	279	88	252	80
Vortioxetine Hydrobromide	Vortioxetine	2469	7	2	6	5
Voxelotor	Voxelotor	55	8	4	5	4
Warfarin	Warfarin	1035	469	78	458	113
Water	Water	78	75356	22329	69344	18364
Water, Sterile	Water, Sterile	423	4976	1969	4573	804
Wound and	Wound and	15	773	223	709	119
Wound Care Preparation	Wound Care Preparation	21	0	0	0	0
Xylitol	Xylitol	12	619	265	541	164

Zafirlukast	Zafirlukast	354	38	2	21	35
Zaleplon	Zaleplon	409	6	1	5	2
Zanamivir	Zanamivir	82	141	22	135	47
Zidovudine	Zidovudine	32	4670	1378	4434	1696
Zileuton	Zileuton	25	6	1	2	6
Zinc	Zinc	1148	2191	1686	1953	374
Zinc Oxide	Zinc Oxide	59	46409	15552	43084	7947
Ziprasidone	Ziprasidone	6152	63	9	61	32
Zolmitriptan	Zolmitriptan	4203	17	2	15	13
Zolpidem	Zolpidem	2785	917	350	892	41
Zonisamide	Zonisamide	5697	107	44	101	50

Supplementary Table S7. MarketScan drugs with no publications in pregnancy, postpartum, 0-1 year, 1-12 years, and 12-18 years.

Drugs with No Pregnancy Publications						
**Original Drug Name**	**Cleaned Drug Name**	**pregnancy frequency**	**all publication**	**pk publication**	**pe publication**	**ct publication**
Loteprednol Etabonate	Loteprednol	532	0	0	0	0
Linaclotide	Linaclotide	481	0	0	0	0
Desoximetasone	Desoximetasone	421	0	0	0	0
Eletriptan Hydrobromide	Eletriptan	309	0	0	0	0
Eszopiclone	Eszopiclone	280	0	0	0	0
Zolmitriptan	Zolmitriptan	183	0	0	0	0
Alclometasone Dipropionate	Alclometasone	178	0	0	0	0
Lifitegrast	Lifitegrast	158	0	0	0	0
Efinaconazole	Efinaconazole	130	0	0	0	0
Difluprednate	Difluprednate	120	0	0	0	0
Oxiconazole Nitrate	Oxiconazole	109	0	0	0	0
Colestipol	Colestipol	104	0	0	0	0
Cariprazine	Cariprazine	73	0	0	0	0
Clocortolone Pivalate	Clocortolone	70	0	0	0	0
Alcaftadine	Alcaftadine	68	0	0	0	0
Epinastine	Epinastine	64	0	0	0	0
Brexpiprazole	Brexpiprazole	61	0	0	0	0
Na Sulf	sodium sulfate	58	0	0	0	0
Tavaborole	Tavaborole	52	0	0	0	0
Frovatriptan	Frovatriptan	46	0	0	0	0
Colistin Sulf	colistin	45	0	0	0	0
Thonzonium Brom	Thonzonium	45	0	0	0	0
Diflorasone Diacetate	Diflorasone	34	0	0	0	0
Suvorexant	Suvorexant	34	0	0	0	0
Eluxadoline	Eluxadoline	32	0	0	0	0
Plecanatide	Plecanatide	32	0	0	0	0
Levomilnacipran	Levomilnacipran	31	0	0	0	0
Dihydroergotamine Mesylate	Dihydroergotamine	26	0	0	0	0
Linagliptin	Linagliptin	26	0	0	0	0
Phendimetrazine	Phendimetrazine	20	0	0	0	0
Naloxegol	Naloxegol	17	0	0	0	0
Flavoxate	Flavoxate	14	0	0	0	0
Ubrogepant	Ubrogepant	14	0	0	0	0
Cevimeline	Cevimeline	11	0	0	0	0
**Drugs with No Postpartum Publications**						
**Original Drug Name**	**Cleaned Drug Name**	**postpartum frequency**	**all publication**	**pk publication**	**pe publication**	**ct publication**
Norgestimate	Norgestimate	26433	0	0	0	0
Benzonatate	Benzonatate	7937	0	0	0	0
Meloxicam	Meloxicam	3703	0	0	0	0
Norelgestromin	Norelgestromin	3592	0	0	0	0
Terconazole	Terconazole	3122	0	0	0	0
Mometasone Furoate	Mometasone	2465	0	0	0	0
Azelastine	Azelastine	2457	0	0	0	0
Azelaic Acid	Azelaic Acid	2295	0	0	0	0

Fluocinonide	Fluocinonide	1902	0	0	0	0
Tizanidine	Tizanidine	1478	0	0	0	0
Olopatadine	Olopatadine	1353	0	0	0	0
Phentermine	Phentermine	1189	0	0	0	0
Sulfacetamide	Sulfacetamide	1039	0	0	0	0
Ciclopirox	Ciclopirox	909	0	0	0	0
Loteprednol Etabonate	Loteprednol	657	0	0	0	0
Linaclotide	Linaclotide	479	0	0	0	0
Calcipotriene	Calcipotriene	448	0	0	0	0
Pimecrolimus	Pimecrolimus	443	0	0	0	0
Varenicline	Varenicline	410	0	0	0	0
Desoximetasone	Desoximetasone	407	0	0	0	0
Econazole Nitrate	econazole	385	0	0	0	0
Metaxalone	Metaxalone	376	0	0	0	0
Triazolam	Triazolam	372	0	0	0	0
Dexlansoprazole	Dexlansoprazole	339	0	0	0	0
Levalbuterol	Levalbuterol	310	0	0	0	0
Etodolac	Etodolac	308	0	0	0	0
Halobetasol	Halobetasol	294	0	0	0	0
Nabumetone	Nabumetone	280	0	0	0	0
Crisaborole	Crisaborole	271	0	0	0	0
Eszopiclone	Eszopiclone	262	0	0	0	0
Fluorometholone	Fluorometholone	260	0	0	0	0
Tazarotene	Tazarotene	237	0	0	0	0
Efinaconazole	Efinaconazole	209	0	0	0	0
Glycopyrrolate	Glycopyrrolate	207	0	0	0	0
Lifitegrast	Lifitegrast	182	0	0	0	0
Dulaglutide	Dulaglutide	175	0	0	0	0
Alclometasone Dipropionate	Alclometasone	174	0	0	0	0
Selenium Sulfide	Selenium Sulfide	168	0	0	0	0
Latanoprost	Latanoprost	140	0	0	0	0
Na Sulf	sodium sulfate	140	0	0	0	0
Difluprednate	Difluprednate	131	0	0	0	0
Brimonidine	Brimonidine	130	0	0	0	0
Rifaximin	Rifaximin	118	0	0	0	0
Naftifine	Naftifine	117	0	0	0	0
Brexpiprazole	Brexpiprazole	115	0	0	0	0
Flurandrenolide	Flurandrenolide	114	0	0	0	0
Colestipol	Colestipol	110	0	0	0	0
Cariprazine	Cariprazine	107	0	0	0	0
Ziprasidone	Ziprasidone	103	0	0	0	0
Empagliflozin	Empagliflozin	102	0	0	0	0
Gatifloxacin	Gatifloxacin	96	0	0	0	0
Glimepiride	Glimepiride	95	0	0	0	0
Tiotropium Bromide	Tiotropium	93	0	0	0	0
Colesevelam	Colesevelam	91	0	0	0	0
Guanfacine	Guanfacine	89	0	0	0	0
Balsalazide Disodium	Balsalazide	87	0	0	0	0
Benztropine Mesylate	Benztropine	85	0	0	0	0
Baloxavir Marboxil	Baloxavir	82	0	0	0	0
Butoconazole Nitrate	Butoconazole	79	0	0	0	0
Oxiconazole Nitrate	Oxiconazole	77	0	0	0	0
Besifloxacin	Besifloxacin	75	0	0	0	0

Segesterone	Segesterone	75	0	0	0	0
Irbesartan	Irbesartan	74	0	0	0	0
Pramipexole Dihydrochloride	pramipexole	72	0	0	0	0
Cetrorelix	Cetrorelix	70	0	0	0	0
Clocortolone Pivalate	Clocortolone	67	0	0	0	0
Apremilast	Apremilast	63	0	0	0	0
Pentosan Polysulfate	Pentosan Polysulfate	62	0	0	0	0
Ganirelix	Ganirelix	59	0	0	0	0
Lubiprostone	Lubiprostone	59	0	0	0	0
Tavaborole	Tavaborole	56	0	0	0	0
Bimatoprost	Bimatoprost	55	0	0	0	0
Secnidazole	Secnidazole	52	0	0	0	0
Bepotastine Besilate	Bepotastine	51	0	0	0	0
Frovatriptan	Frovatriptan	50	0	0	0	0
Bethanechol	Bethanechol	49	0	0	0	0
Ciclesonide	Ciclesonide	49	0	0	0	0
Halcinonide	Halcinonide	48	0	0	0	0
Tolterodine	Tolterodine	47	0	0	0	0
Carbinoxamine	Carbinoxamine	41	0	0	0	0
Solifenacin	Solifenacin	41	0	0	0	0
Icosapent Ethyl	Icosapent Ethyl	41	0	0	0	0
Luliconazole	Luliconazole	40	0	0	0	0
Colistin Sulf	colistin	38	0	0	0	0
Fidaxomicin	Fidaxomicin	38	0	0	0	0
Thonzonium Brom	Thonzonium	38	0	0	0	0
Canagliflozin	Canagliflozin	37	0	0	0	0
Clorazepate Dipotassium	Clorazepate	37	0	0	0	0
Bromfenac	Bromfenac	35	0	0	0	0
Diflunisal	Diflunisal	35	0	0	0	0
Flunisolide	Flunisolide	35	0	0	0	0
Mirabegron	Mirabegron	34	0	0	0	0
Suvorexant	Suvorexant	34	0	0	0	0
Dihydroergotamine Mesylate	Dihydroergotamine	33	0	0	0	0
Lovastatin	Lovastatin	32	0	0	0	0
Diflorasone Diacetate	Diflorasone	31	0	0	0	0
Iodoquinol	Iodoquinol	30	0	0	0	0
Lorcaserin	Lorcaserin	29	0	0	0	0
Plecanatide	Plecanatide	29	0	0	0	0
Elagolix	Elagolix	28	0	0	0	0
Phendimetrazine	Phendimetrazine	28	0	0	0	0
Travoprost	Travoprost	28	0	0	0	0
Alcaftadine	Alcaftadine	27	0	0	0	0
Oxaprozin	Oxaprozin	27	0	0	0	0
Exenatide	Exenatide	26	0	0	0	0
Levomilnacipran	Levomilnacipran	25	0	0	0	0
Eluxadoline	Eluxadoline	23	0	0	0	0
Guselkumab	Guselkumab	23	0	0	0	0
Indapamide	Indapamide	23	0	0	0	0
Insulin Glulisine	Insulin Glulisine	23	0	0	0	0
Memantine	Memantine	21	0	0	0	0
Trifarotene	Trifarotene	20	0	0	0	0
Umeclidinium	Umeclidinium	20	0	0	0	0
Zafirlukast	Zafirlukast	20	0	0	0	0

Acarbose	Acarbose	19	0	0	0	0
Asenapine	Asenapine	18	0	0	0	0
Capecitabine	Capecitabine	18	0	0	0	0
Chlophedianol	Chlophedianol	18	0	0	0	0
Flavoxate	Flavoxate	18	0	0	0	0
Sevelamer	Sevelamer	18	0	0	0	0
Griseofulvin	Griseofulvin	15	0	0	0	0
Teriflunomide	Teriflunomide	15	0	0	0	0
Sulconazole Nitrate	Sulconazole	15	0	0	0	0
Ingenol Mebutate	Ingenol Mebutate	15	0	0	0	0
Brinzolamide	Brinzolamide	14	0	0	0	0
Risankizumab-rzaa	Risankizumab	14	0	0	0	0
Retapamulin	Retapamulin	13	0	0	0	0
Vilanterol	Vilanterol	13	0	0	0	0
Apraclonidine	Apraclonidine	12	0	0	0	0
Flibanserin	Flibanserin	12	0	0	0	0
Linagliptin	Linagliptin	12	0	0	0	0
Naloxegol	Naloxegol	12	0	0	0	0
Nepafenac	Nepafenac	12	0	0	0	0
Felodipine	Felodipine	11	0	0	0	0

Drugs with No Pediatric 0-1 Year Publications

Original Drug Name	Cleaned Drug Name	peds: 0~1 frequency	all publication	pk publication	pe publication	ct publication
Olopatadine	Olopatadine	358	0	0	0	0
Oxiconazole Nitrate	Oxiconazole	199	0	0	0	0
Flurandrenolide	Flurandrenolide	76	0	0	0	0
Thonzonium Brom	Thonzonium	35	0	0	0	0
Naftifine	Naftifine	21	0	0	0	0
Epoetin Alfa-epbx	Epoetin Alfa-epbx	16	0	0	0	0
Chlophedianol	Chlophedianol	13	0	0	0	0
Terconazole	Terconazole	12	0	0	0	0

Drugs with No Pediatric 1-12 Years Publications

Original Drug Name	Cleaned Drug Name	peds: 1~12 frequency	all publication	pk publication	pe publication	ct publication
Thonzonium Brom	Thonzonium	1736	0	0	0	0
Chlophedianol	Chlophedianol	1146	0	0	0	0
Na Phos, Monoba	Monobasic Sodium Phosphate	146	0	0	0	0
Thonzylamine	Thonzylamine	126	0	0	0	0
Antihemophilic Factor VIII Fc Fusion Protein Recom	Recombinant Factor VIII Fc Fusion Protein	97	0	0	0	0
Na Sulf	sodium sulfate	71	0	0	0	0
p-Phenolsulfonic Acid	p-Phenolsulfonic Acid	41	0	0	0	0
Oxaprozin	Oxaprozin	37	0	0	0	0
Darifenacin Hydrobromide	Darifenacin	35	0	0	0	0
Amcinonide	Amcinonide	28	0	0	0	0
Docosanol	Docosanol	21	0	0	0	0
C30-45 Alkyl Cetearyl Dim	C30-45 Alkyl Cetearyl Dimethicone	17	0	0	0	0
Levomilnacipran	Levomilnacipran	13	0	0	0	0

Drugs with No Pediatric 12-18 Years Publications

Original Drug Name	Cleaned Drug Name	peds: 12~18 frequency	all publication	pk publication	pe publication	ct publication

Alclometasone Dipropionate	Alclometasone	6007	0	0	0	0
Metaxalone	Metaxalone	4393	0	0	0	0
Linaclotide	Linaclotide	1935	0	0	0	0
Thonzonium Brom	Thonzonium	883	0	0	0	0
Na Sulf	sodium sulfate	275	0	0	0	0
Levomilnacipran	Levomilnacipran	214	0	0	0	0
Ubrogepant	Ubrogepant	180	0	0	0	0
Iloperidone	Iloperidone	150	0	0	0	0
Eluxadoline	Eluxadoline	139	0	0	0	0
Na Phos, Monoba	Monobasic Sodium Phosphate	108	0	0	0	0
Butoconazole Nitrate	Butoconazole	98	0	0	0	0
Octisalate	Octisalate	93	0	0	0	0
Plecanatide	Plecanatide	93	0	0	0	0
Nepafenac	Nepafenac	87	0	0	0	0
Antihemophilic Factor VIII Fc Fusion Protein Recom	Recombinant Factor VIII Fc Fusion Protein	86	0	0	0	0
Gabapentin Enacarbil	Gabapentin Enacarbil	67	0	0	0	0
Methazolamide	Methazolamide	61	0	0	0	0
Serdexmethylphenidate	Serdexmethylphenidate	49	0	0	0	0
p-Phenolsulfonic Acid	p-Phenolsulfonic Acid	47	0	0	0	0
Naloxegol	Naloxegol	39	0	0	0	0
Na Phos, Mo	Sodium Phosphate	34	0	0	0	0
Salsalate	Salsalate	27	0	0	0	0
Solriamfetol	Solriamfetol	27	0	0	0	0
Risankizumab-rzaa	Risankizumab	26	0	0	0	0
Olodaterol	Olodaterol	25	0	0	0	0
Guselkumab	Guselkumab	16	0	0	0	0
Peginterferon Beta-1a	Peginterferon Beta-1a	16	0	0	0	0
Thonzylamine	Thonzylamine	14	0	0	0	0
Arformoterol	Arformoterol	13	0	0	0	0
Cevimeline	Cevimeline	13	0	0	0	0
Indapamide	Indapamide	13	0	0	0	0
Patiromer	Patiromer	13	0	0	0	0

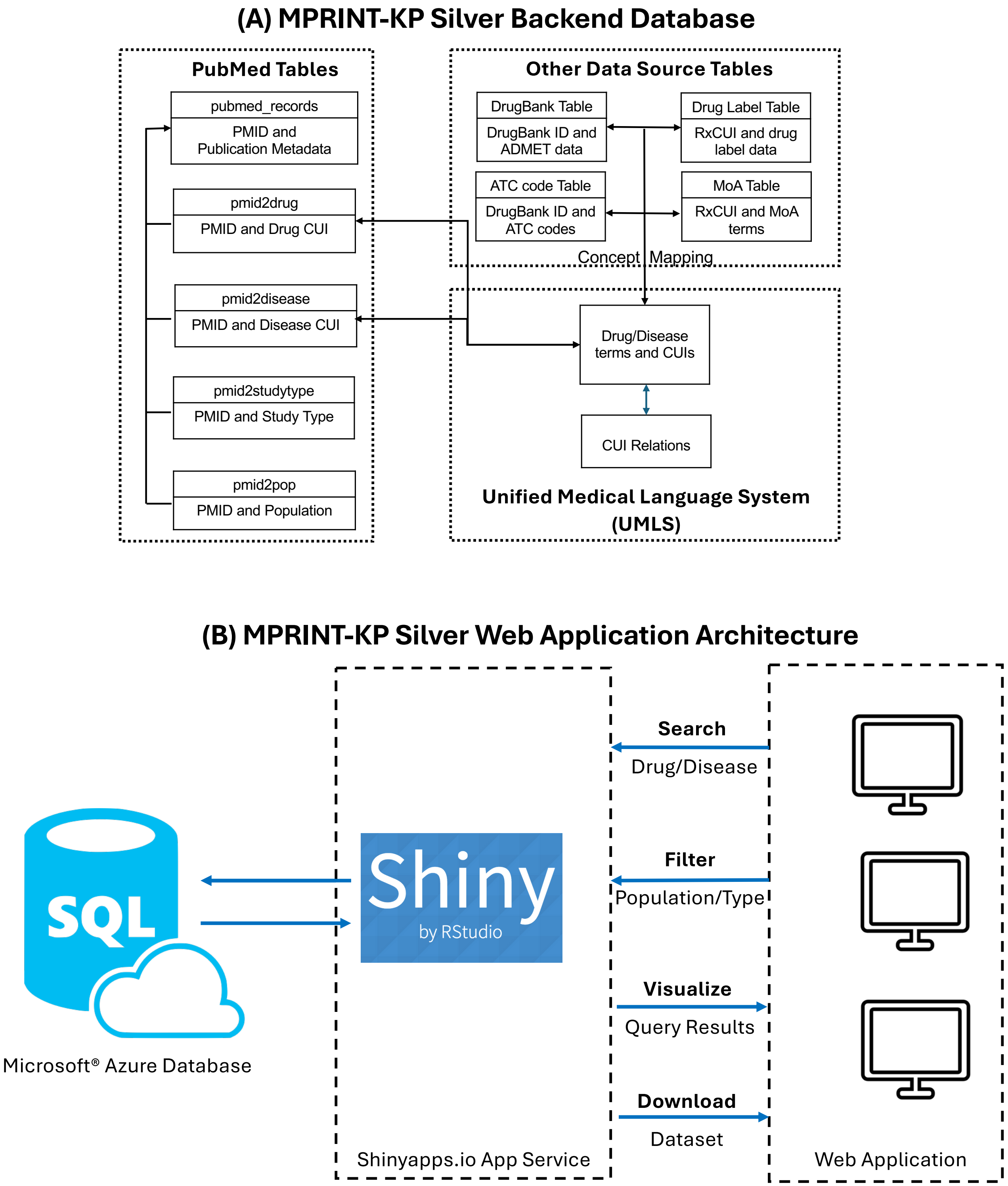

Supplementary Figure S1. MPRINT-KP Silver Database. (A) Silver backend database, ADMET: Absorption, Distribution, Metabolism, and Transportation; ATC: Anatomical Therapeutic Chemical; MoA: Mechanism of Action. (B) Web application architecture.

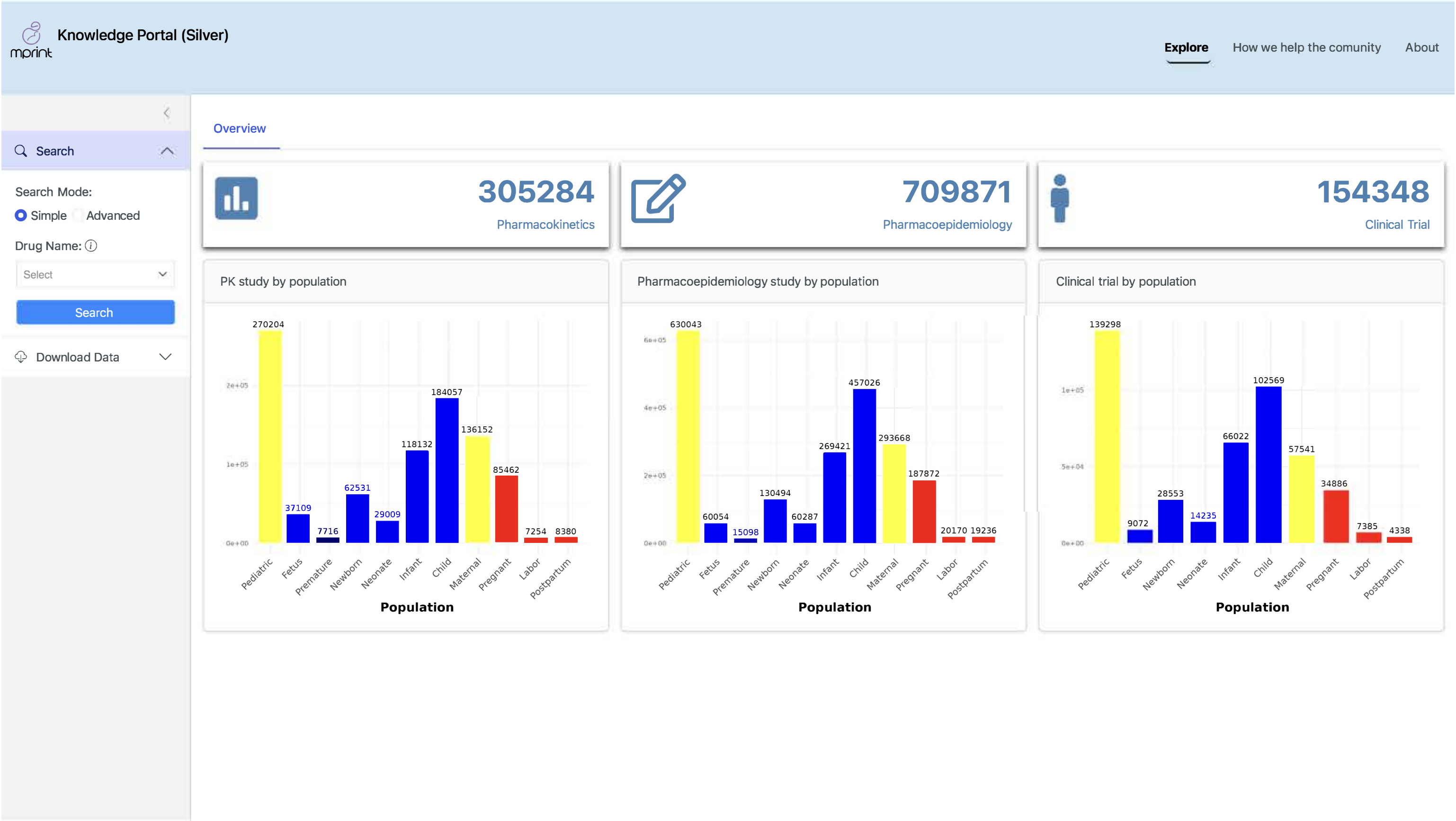

Supplementary Figure S2. Screenshots of MPRINT-KP Silver Schemes.

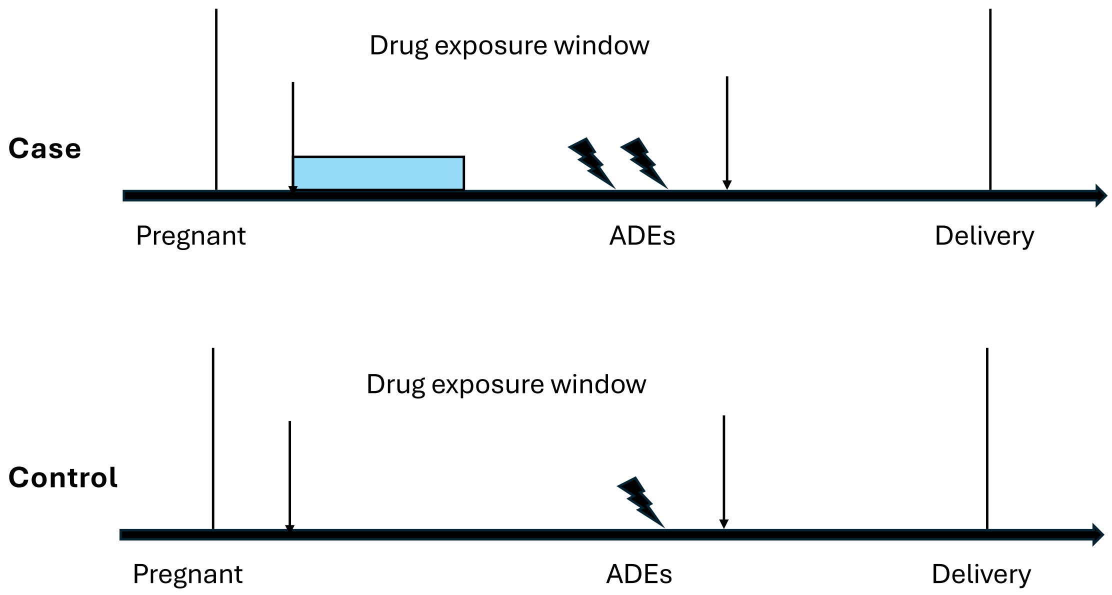

Supplementary Figure S3. MarketScan-based pharmaco-epidemiology nested case control study design.

Supplementary Note S1. MPRINT-KP Silver Database pharmacological knowledge.

MPRINT-KP Silver integrates pharmacological knowledge from two other sources: DrugBank[1] and United States Food and Drug Administration (FDA) Drug Labels[2]. This information was organized into tables dedicated to specific knowledge domains: *atc*, *moa*, *admet*, and *spl*. The *atc* table links drug concepts identified by their Unified Medical Language System (UMLS) Concept Unique Identifier (CUI)[3] to their corresponding Anatomical Therapeutic Chemical (ATC)[4] codes, which provide a standardized classification of drugs based on their therapeutic use and site of action. The *moa* table directly captures the Mechanism of Action (MoA) information, which shows how drug exert their effects at a molecular or cellular level, from DailyMed[2]. The *admet* table stores data on drug absorption, distribution, metabolism, and excretion and transport from DrugBank, including parameters such as half-life, clearance, and volume of distribution. Finally, the *spl* table contains structured product labeling information extracted from pharmaceutical product labels, specifically focusing on sections relevant to maternal and pediatric use, such as "Use in Pregnancy," "Use in Lactation," and "Pediatric Use." The linkage among these tables and the core publication table is through UMLS CUI.

MPRINT-KP Silver adapted UMLS terminology as its core framework to ensure consistency and interoperability. The *concept* table serves as a central repository for biomedical concepts, in which each concept and its corresponding synonyms are uniquely identified by its cui. This table stores the concept names and the general concept type (e.g., "Disease," "Drug," "MoA"). Using UMLS CUIs as primary keys establishes an unambiguous and standardized representation of biomedical entities and facilitates cross-database analyses.

The relation table, *rel*, in MPRINT-KP Silver plays a crucial role in organizing and structuring biomedical concepts. It establishes hierarchical relationships between various medical terms. By capturing these relationships, MPRINT-KP Silver leverages the structured information provided by the *rel* table to offer advanced search functionalities, considering the hierarchical relationships between concepts. For instance, when users use “hypertension” as the search term, studies involving all subtypes, such as chronic hypertension and pre-eclampsia, will be included in the search result.

Supplementary Note S2. MPRINT-KP Silver User Interface

The MPRINT-KP Silver utilizes a web portal built within the R Shiny framework[5] to allow users to navigate and search the knowledgebase (Web application can be accessed via https://www.mprint.org/knowledgebase. The dataset can be downloaded via https://github.com/langli-lab/mprint-kp-data). **Supplementary Figure S2**, MPRINT-KP Silver Schemes, illustrates the basic features of this web application. Users can search for a drug of interest, a disease of interest, or a combination of a drug and a disease from controlled lexicons via a type-ahead feature. The web portal will return all papers matching the search criteria in the Publication tab. Details of the matched PubMed abstracts, including their titles, publication year, study types, studied populations, drugs and diseases are provided. Users can filter publications by the study types and populations. External links to PubMed for each publication are also provided so that users can easily find their full text of interest. In addition, custom sorting of the results is performed by clicking the column name of the results table.

If users include a drug in their search, an individual Drug tab presents pharmacological information about the drug of interest. Users can also identify drug classifications including ATC codes, MoAs and ADMET. In particular, the web portal provides relevant information on the maternal and pediatric use of drug products from drug labels.

Reference

1. Online D. DrugBank Online. DrugBank. Accessed May 5, 2025. https://go.drugbank.com/

2. Medicine USNLo. DailyMed: Drug Label Information. National Institutes of Health (NIH). Accessed May 5, 2025. https://www.dailymed.nlm.nih.gov/dailymed/

3. Medicine USNLo. Unified Medical Language System (UMLS) Metathesaurus. National Institutes of Health (NIH). Accessed May 5, 2025. https://www.nlm.nih.gov/research/umls/index.html

4. Methodology WCCfDS. ATC/DDD Index 2025. WHO. Accessed May 5, 2025. https://atcddd.fhi.no/atc_ddd_index/

5. Posit P. Shiny: Web Application Framework for R. Previously RStudio Inc. Accessed May 5, 2025. https://shiny.posit.co/
